# Clinical Presentation, Investigation Findings, and Treatment Outcomes of Spontaneous Intracranial Hypotension Syndrome: A Systematic Review and Meta-analysis

**DOI:** 10.1001/jamaneurol.2020.4799

**Published:** 2021-01-04

**Authors:** Linda D’Antona, Melida Andrea Jaime Merchan, Anna Vassiliou, Laurence Dale Watkins, Indran Davagnanam, Ahmed Kassem Toma, Manjit Singh Matharu

**Affiliations:** 1 Victor Horsley Department of Neurosurgery, National Hospital for Neurology and Neurosurgery, London, United Kingdom; 2 UCL Queen Square Institute of Neurology, London, United Kingdom; 3 Department of Neuroradiology, National Hospital for Neurology and Neurosurgery, London, United Kingdom; 4 Headache and Facial Pain Group, National Hospital for Neurology and Neurosurgery, London, United Kingdom

## Abstract

**Importance:**

Spontaneous intracranial hypotension (SIH) is a highly disabling but often misdiagnosed disorder. The best management options for patients with SIH are still uncertain.

**Objective:**

To provide an objective summary of the available evidence on the clinical presentation, investigations findings, and treatment outcomes for SIH.

**Data Sources:**

Preferred Reporting Items for Systematic Reviews and Meta-analyses (PRISMA) reporting guideline–compliant systematic review and meta-analysis of the literature on SIH. Three databases were searched from inception to April 30, 2020: PubMed/MEDLINE, Embase, and Cochrane. The following search terms were used in each database: *spontaneous intracranial hypotension*, *low CSF syndrome*, *low CSF pressure syndrome*, *low CSF volume syndrome*, *intracranial hypotension*, *low CSF pressure*, *low CSF volume*, *CSF hypovolemia*, *CSF hypovolaemia*, *spontaneous spinal CSF leak*, *spinal CSF leak*, and *CSF leak syndrome*.

**Study Selection:**

Original studies in English language reporting 10 or more patients with SIH were selected by consensus.

**Data Extraction and Synthesis:**

Data on clinical presentation, investigations findings, and treatment outcomes were collected and summarized by multiple observers. Random-effect meta-analyses were used to calculate pooled estimates of means and proportions.

**Main Outcomes and Measures:**

The predetermined main outcomes were the pooled estimate proportions of symptoms of SIH, imaging findings (brain and spinal imaging), and treatment outcomes (conservative, epidural blood patches, and surgical).

**Results:**

Of 6878 articles, 144 met the selection criteria and reported on average 53 patients with SIH each (range, 10-568 patients). The most common symptoms were orthostatic headache (92% [95% CI, 87%-96%]), nausea (54% [95% CI, 46%-62%]), and neck pain/stiffness (43% [95% CI, 32%-53%]). Brain magnetic resonance imaging was the most sensitive investigation, with diffuse pachymeningeal enhancement identified in 73% (95% CI, 67%-80%) of patients. Brain magnetic resonance imaging findings were normal in 19% (95% CI, 13%-24%) of patients. Spinal neuroimaging identified extradural cerebrospinal fluid in 48% to 76% of patients. Digital subtraction myelography and magnetic resonance myelography with intrathecal gadolinium had high sensitivity in identifying the exact leak site. Lumbar puncture opening pressures were low, normal (60-200 mm H_2_O), and high in 67% (95% CI, 54%-80%), 32% (95% CI, 20%-44%), and 3% (95% CI, 1%-6%), respectively. Conservative treatment was effective in 28% (95% CI, 18%-37%) of patients and a single epidural blood patch was successful in 64% (95% CI, 56%-72%). Large epidural blood patches (>20 mL) had better success rates than small epidural blood patches (77% [95% CI, 63%-91%] and 66% [95% CI, 55%-77%], respectively).

**Conclusions and Relevance:**

Spontaneous intracranial hypotension should not be excluded on the basis of a nonorthostatic headache, normal neuroimaging findings, or normal lumbar puncture opening pressure. Despite the heterogeneous nature of the studies available in the literature and the lack of controlled interventional studies, this systematic review offers a comprehensive and objective summary of the evidence on SIH that could be useful in guiding clinical practice and future research.

## Key Points

**Question:**

What are the clinical presentation, investigation findings, and treatment outcomes of spontaneous intracranial hypotension?

**Findings:**

This systematic review and meta-analysis of 144 articles provides a summary of the evidence on spontaneous intracranial hypotension and demonstrates that a significant minority of patients may have nonorthostatic headache, normal lumbar punctures, or normal imaging results. Treatment with 1 epidural blood patch is often successful, with large-volume blood patches giving better outcomes.

**Meaning:**

A diagnosis of spontaneous intracranial hypotension should not be excluded based on the absence of one of its typical features; large epidural blood patches should be attempted if conservative treatment has failed.

## Teaser

This systematic review and meta-analysis provides an objective summary of the available evidence on the clinical presentation, investigations findings, and treatment outcomes for spontaneous intracranial hypotension.

## Introduction

The term *spontaneous intracranial hypotension* (SIH) defines a clinical condition characterized by debilitating postural headaches secondary to spontaneous spinal cerebrospinal fluid (CSF) leak and/or CSF hypotension. According to the *International Classification of Headache Disorders* (*ICHD*), third edition, SIH is diagnosed when headache has developed spontaneously and in temporal relation to a CSF leak (evident on imaging) and/or CSF hypotension (lumbar puncture opening pressure <60 mm CSF).^1^

Spontaneous intracranial hypotension is a highly misdiagnosed and underdiagnosed disorder.^2^ Estimates suggest that SIH is not uncommon with an annual incidence of 5 per 100 000 individuals every year, half the incidence of subarachnoid hemorrhage.^3^ Despite the lack of objective evidence on the effect of SIH on patients’ quality of life, the orthostatic headache typical of this condition makes SIH debilitating, affecting patients during their most active hours. The exact pathogenetic mechanism of SIH is unknown, and this lack of knowledge has led to a series of misconceptions.^4^ Moreover, the *ICHD* diagnostic criteria for SIH have changed significantly throughout the last few decades, and alternative diagnostic criteria have been proposed.^5,6^ These factors have probably contributed to the current uncertainty on how to reliably diagnose SIH and effectively treat these patients.

Despite the increasing number of publications on SIH throughout the last 2 decades, this is the first comprehensive systematic review on this condition, to our knowledge. The aim of this study is to summarize the available evidence on clinical presentation, diagnostic investigations, and treatment outcomes for SIH. Specific questions addressed in this systematic review are: (1) What are the signs and symptoms of SIH and how frequently do they occur? (2) What is the sensitivity of brain magnetic resonance imaging (MRI), spinal imaging, and lumbar puncture opening pressures in detecting signs of SIH? (3) What is the most sensitive spinal imaging technique to detect CSF leaks? (4) What are the outcomes of conservative treatment and epidural blood patches (EBP) in patients with SIH? (5) What is the most efficient EBP technique in SIH (nontargeted vs targeted, small- vs large-volume patches)?

## Methods

This is a systematic review and meta-analysis of the clinical presentation, investigations findings, and treatment outcomes of SIH. This study is compliant with the Preferred Reporting Items for Systematic Reviews and Meta-analyses (PRISMA) reporting guideline and is registered on PROSPERO (CRD42019147300).^7^

### Search Strategy

Three electronic databases were searched for studies on SIH or spontaneous CSF leaks (PubMed/MEDLINE, Embase, and Cochrane). The search did not have a start date limit and was last updated on April 30, 2020. The following search terms were used in each database: *spontaneous intracranial hypotension*, *low CSF syndrome*, *low CSF pressure syndrome*, *low CSF volume syndrome*, *intracranial hypotension*, *low CSF pressure*, *low CSF volume*, *CSF hypovolemia*, *CSF hypovolaemia*, *spontaneous spinal CSF leak*, *spinal CSF leak*, and *CSF leak syndrome*.

### Selection Criteria

The inclusion criteria were (1) topic SIH or spontaneous CSF leaks, (2) English language, (3) original study, and (4) reporting at least 10 patients. Articles reporting intracranial hypotension or CSF leaks secondary to other causes (traumatic or iatrogenic) were excluded. Articles reporting a mixed population of patients affected by spontaneous and secondary leaks were included if they were compliant with the inclusion criteria and it was possible to clearly distinguish the characteristics of the spontaneous CSF leak group. Because of the comprehensive nature of the systematic review and the large volume of articles obtained from the search, it was not possible to include articles written in languages other than English. Articles published ahead of print and any study design were considered. Reference lists of the selected articles were screened. Case reports and small case series (reporting less than 10 patients) were screened for unusual findings before exclusion. Search and screening were performed by L.D. and revised by M.S.M.; conflicts on inclusion of data were resolved by consensus with a third author (A.K.T. or I.D.).

The risk of bias of the selected studies was assessed through the National Institutes of Health Quality Assessment Tool for Case Series Studies.^8^ Articles were rated as good, fair, or poor by 2 independent assessors (L.D. and M.A.J.M.). Disagreements were settled through discussion between the 2 authors. To prevent bias due to duplicated information, only the biggest case series per author/research group (highest number of patients) was included in each analysis.

### Data Extraction

The selected articles were assessed to identify the presence of information on each of the following domains: study design, demographic characteristics, risk factors, clinical presentation, brain MRI, spinal imaging, CSF leak location, CSF pressure, treatments, and outcomes (eTable 1 in the Supplement for detailed list of variables). Both summary data and patient-level data were extracted from published reports. The data extraction was performed by L.D. and revised by M.A.J.M., A.V., M.S.M. (all domains), and I.D. (imaging findings).

### Statistical Analysis

Meta-analyses with the commands *metaprop* and *metan* of the software Stata version 15.0 (StataCorp) were used to calculate pooled estimates of proportions (95% CI) and pooled estimates of means (95% CI) of demographic characteristics (age and sex), clinical presentation, investigations findings, and treatment outcomes.^9^ All summary means and proportions included in the results are pooled estimates obtained with meta-analyses. Specific inclusion criteria for each meta-analysis are detailed in eTable 2 in the Supplement. The variability within studies and between studies was assessed with the *I^2^* estimate of heterogeneity. Given the heterogeneity of the selected studies, a random-effects analysis was chosen for all meta-analyses. Microsoft Excel (version 16.25 for macOS) and Stata (version 15.0; StataCorp) were used for the data collection and statistical analysis.

## Results

The screening and selection of articles is described in eFigure 1 in the Supplement. One hundred forty-four articles reporting a mean (range) of 53 (10-568) patients with SIH each met the selection criteria. eTable 3 in the Supplement provides a complete list of the selected studies and information on their inclusion/exclusion from the meta-analyses. Forest plots of all the meta-analyses are available in eFigures 2-20 in the Supplement.

Of 144 articles, none were controlled interventional studies, 90 (62.5%) were retrospective, 21 (14.6%) were prospective, and the remaining articles (33 [22.9%]) did not clearly specify the type of data collection. The *ICHD* diagnostic criteria were used to diagnose SIH in 49 articles (34%) (*ICHD-II*, 33 articles [22.9%]; *ICHD-III beta*, 7 articles [4.9%]; *ICHD-III*, 9 articles [6.3%]).^1,10,11^ The 2008 and 2011 Schievink diagnostic criteria were used in 17 articles (11.8%), other criteria were used in 31 articles (21.5%) and the diagnostic criteria were not clearly specified in the remaining 47 (32.6%).^5,6^ The selected articles were rated as fair (93 [64.6%]) or good (51 [35.4%]) quality according to 2 independent assessors using the National Institutes of Health Quality Assessment Tool for Case Series Studies.^8^

### Clinical Presentation

The mean age of patients was 42.5 years (95% CI, 41.1-43.9; *I^2^* = 79.3%) with a range of 2 to 88 years.^12^ The proportion of female individuals was 63% (95% CI, 60%-66%; *I^2^* = 52.4). Connective tissue disorders, spinal pathologies (ie, osteophytes, disc prolapse, and discogenic micro spurs) and bariatric surgery were identified as risk factors for SIH and reported by several authors.^13,14,15,16,17,18,19,20,21,22,23^

**Figure 1.**
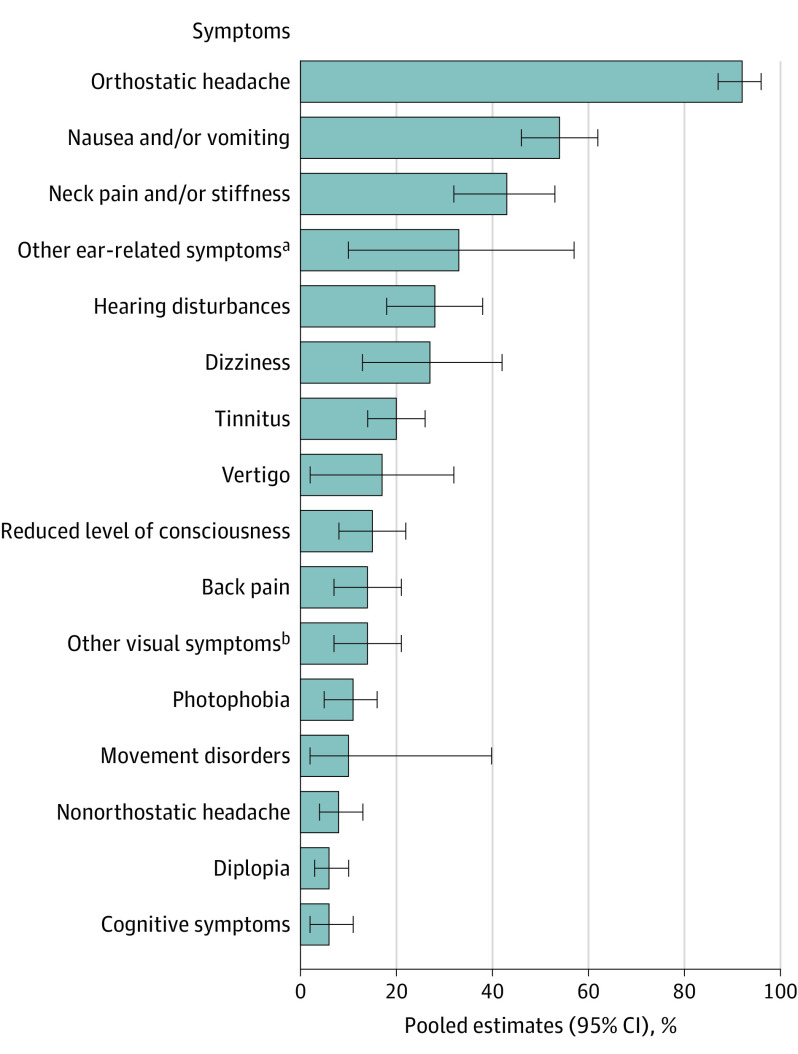
Signs and Symptoms of Spontaneous Intracranial Hypotension Percentages indicate the pooled estimates of proportions. ^a^Other ear-related symptoms included aural fullness, hyperacusis, or unspecified. ^b^Other visual symptoms included blurred vision, nystagmus, and/or visual loss.

Table 1 and Figure 1 show a summary of the clinical characteristics of 1694 patients with SIH (33 articles). The duration of symptoms at the time of diagnosis and/or treatment was variable ranging from 1 day to 19.7 years,^24,25^ with a pooled estimated mean of 31.7 days (95% CI, 24.8-38.5; *I^2^* = 97.4%). Headache was the most frequent symptom, being present in 97% (95% CI, 94%-99%; *I^2^* = 52.2%) of patients and was most commonly orthostatic (92%; 95% CI, 87%-96%; *I^2^* = 80.9); however, 3% (95% CI, 1%-6%; *I^2^* = 52.2%) of patients did not report any headache. The headache location was most commonly diffuse, occipital, or frontal.^15,26,27,28,29^ Table 1 shows all the other signs and symptoms reported and their pooled estimates of proportions.

**Table 1.** Clinical Presentation of Patients With Spontaneous Intracranial Hypotension

Characteristic^a^	Patients, No. (%)	Pooled estimates of proportions (95% CI)
Headache (33 articles, 1694 patients)		
Headache	1671 (98.6)	97 (94-99)
No headache	23 (1.4)	3 (1-6)
Orthostatic headache (among patients with headache)	1632 (97.7)	92 (87-96)
Nonorthostatic headache (among patients with headache)	39 (2.3)	8 (4-13)
Headache location (5 articles, 234 patients)		
Diffuse/holocranial	72 (30.8)	30 (13-46)
Occipital	65 (27.8)	33 (19-46)
Frontal	54 (23.1)	21 (10-32)
Fronto-occipital	9 (3.8)	11 (4-18)
Temporal	6 (2.6)	8 (2-13)
Undefined	28 (12.0)	NA
Other signs/symptoms (32 articles, 1531 patients)		
Nausea/vomiting	775 (50.6)	54 (46-62)
Neck pain/stiffness	507 (33.1)	43 (32-53)
Tinnitus	295 (19.3)	20 (14-26)
Dizziness	216 (14.1)	27 (13-42)
Hearing disturbances	163 (10.7)	28 (18-38)
Photophobia	70 (4.6)	11 (5-16)
Other visual symptoms^b^	63 (4.1)	14 (7-21)
Diplopia	60 (3.9)	6 (3-10)
Vertigo	58 (3.8)	17 (2-32)
Back pain	49 (3.2)	14 (7-21)
Cognitive symptoms^c^	40 (2.6)	6 (2-11)
Other ear-related symptoms^d^	38 (2.5)	33 (10-57)
Reduced level of consciousness	27 (1.8)	15 (8-22)
Movement disorders^e^	18 (1.2)	10 (2-40)

Abbreviation: NA, not applicable.

^a^ Less commonly reported symptoms were dysgeusia, sleepiness, cranial nerve palsy (unspecified), fever, radicular symptoms, galactorrhea, incontinence, fatigue, vocal tics, convulsions, facial spasms/numbness/pain, and dysphagia.

^b^ Other visual symptoms included blurred vision, nystagmus, and/or visual loss.

^c^ Cognitive symptoms included cognitive impairment, behavioral changes, memory, and/or slow thinking.

^d^ Other ear-related symptoms included aural fullness, hyperacusis, or unspecified.

^e^ Movement disorders included gait disorder, ataxia, dysarthria, tremor, bradykinesia, and/or poor balance.

### Assessment and Diagnosis

**Figure 2.**
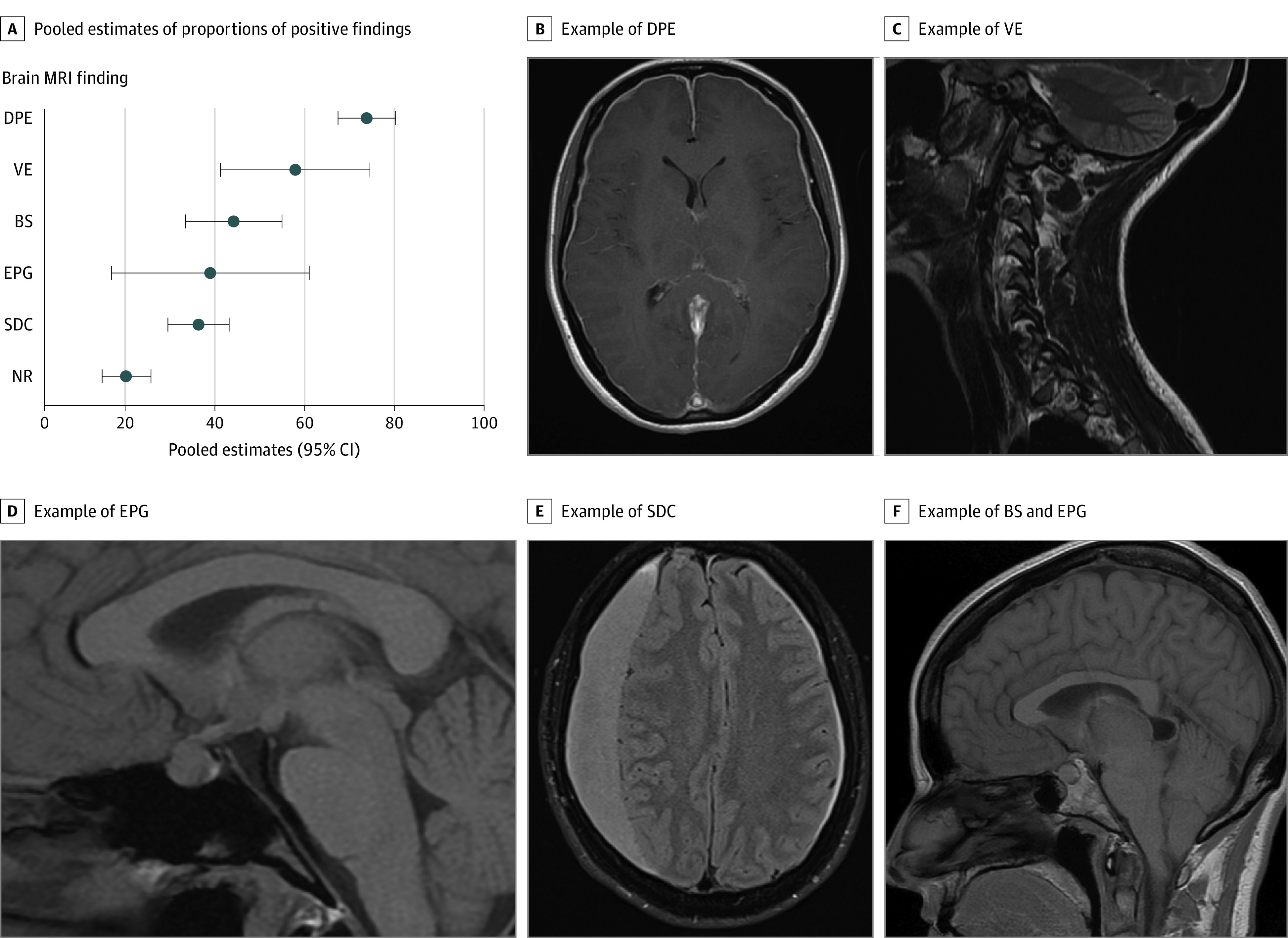
Brain Magnetic Resonance Imaging (MRI) Findings of Spontaneous Intracranial Hypotension A, Pooled estimates of proportions of positive findings in spontaneous intracranial hypotension. B, Example of diffuse pachymeningeal enhancement (DPE) in T1-weighted axial MRI sequence. C, Example of venous engorgement (VE, transverse sinus venous distension sign) in T2-weighted sagittal MRI sequence. D, Example of enlarged pituitary gland (EPG) in T1-weighted sagittal MRI sequence. E, Example of subdural collection (SDC) in T2-weighted axial MRI sequence. F, Example of brain sagging (BS) and enlarged pituitary gland (EPG) in T1-weighted sagittal MRI sequence. NR indicates normal.

Thirty-eight articles were selected for the description of the brain MRI findings of 2078 patients diagnosed as having SIH: 73% (95% CI, 67%-80%; *I^2^* = 90.9%) showed diffuse gadolinium pachymeningeal enhancement, 35% (95% CI, 28%-42%; *I^2^* = 88.5%) showed subdural collections, 43% (95% CI, 32%-54%; *I^2^* = 95.8%) showed brain sagging, 57% (95% CI, 40%-74%; *I^2^* = 94.8%) showed signs of venous engorgement, and 38% (95% CI, 15%-60%; *I^2^* = 99.2%) showed pituitary gland enlargement. Brain MRI results were normal in 19% (95% CI, 13-24; *I^2^* = 59.3) of patients. Figure 2 shows a summary of the main brain MRI findings.

**Figure 3.**
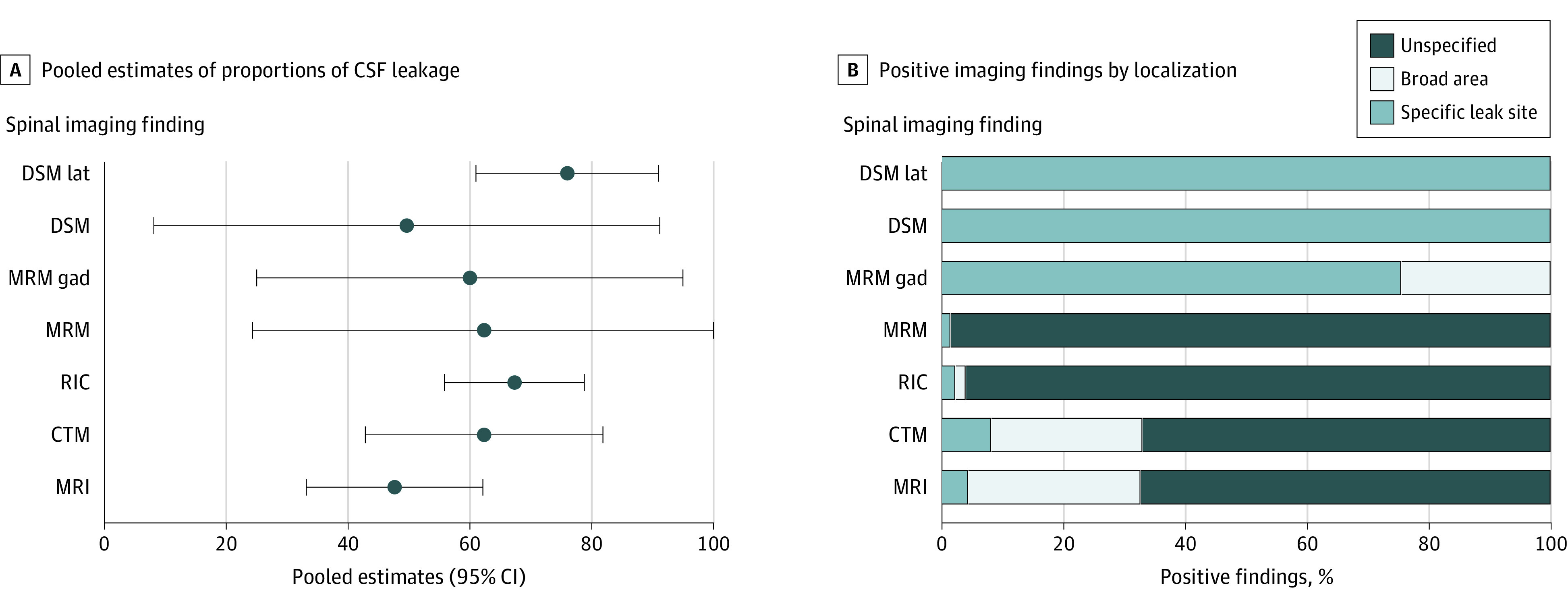
Spinal Imaging Findings A, Pooled estimates of proportions (95% CI) of cerebrospinal fluid (CSF) leakage found in spinal imaging investigations. B, Positive imaging findings stratified by type of CSF leak localization: specific leak site, broad area, and unspecified. CTM indicates computed tomography myelography; DSM, digital subtraction myelography; DSM lat, digital subtraction myelography in lateral decubitus position; MRI, magnetic resonance imaging; MRM, magnetic resonance myelography; MRM gad, magnetic resonance myelography with intrathecal gadolinium; RIC, radionuclide cisternography.

The sensitivity of spinal investigations for identifying CSF leaks (defined as detection of extradural CSF) was analyzed for spinal MRI, computed tomography myelography, radionuclide cisternography, magnetic resonance (MR) myelography (with and without intrathecal gadolinium), and digital subtraction myelography (DSM). Presence of extradural CSF was detected in 48% to 76% of cases (Figure 3A). The techniques able to identify the specific leak site most frequently were the MR myelography with intrathecal gadolinium and the DSM; however, only 4 studies (2.8%) and 3 studies (2.1%), respectively, underwent these types of spinal investigation. Among the other spinal investigations described, dynamic CT myelography was reported to be useful for the detection the exact leak site in fast CSF leaks, but none of these studies met the inclusion criteria for meta-analysis.^17,30,31^

Twenty-eight articles describing 1523 leaks were selected to summarize the location of spinal CSF leaks. The most common leak location was the thoracic spine (41%; 95% CI, 29%-52%; *I^2^* = 97.3%) followed by the cervicothoracic junction (25%; 95% CI, 17%-32%; *I^2^* = 88.0%), the cervical spine (14%; 95% CI, 10%-17%; *I^2^* = 70.7%), and the lumbar spine (12%; 95% CI, 8%-16%; *I^2^* = 82.5%). Leaks were reported to be multiple in 24% (95% CI, 15%-33%; *I^2^* = 88.7%) of cases.

Twenty-one articles, including 738 patients, were selected to analyze the findings of lumbar puncture opening pressure: 67% (95% CI, 54%-80%; *I^2^* = 94.7%) of patients had low pressure (<60 mm H_2_O), 32% (95% CI, 20%-44%; *I^2^* = 94.3%) had normal pressure (60-200 mm H_2_O), and 3% (95% CI, 0%-6%; *I^2^* = 43.4%) had high pressure (>200 mm H_2_O). The highest reported opening pressure was 228 mm H_2_O.^32^

### Treatment

Conservative treatment was attempted in 881 patients for a period ranging from 7 to 9 weeks. This most commonly consisted of bed rest and hydration (Table 2). Authors reported a successful conservative treatment (resolution of symptoms with no further intervention needed) in 28% (95% CI, 18%-37%; *I^2^* = 91.4%) of patients.

**Table 2.** Treatment of Spontaneous Intracranial Hypotension and Outcomes

Treatment^a^	Patients, No. (%)	Pooled estimates of proportions (95% CI)	*I^2^*
Conservative treatment (17 articles, 748 patients)			
Effective	183 (24.5)	28 (18-37)	91.5
Ineffective	565 (75.5)	72 (63-82)	91.5
Type of conservative treatment			
Bed rest	658 (88.0)	NA	NA
Hydration	621 (83.0)	NA	NA
Analgesia	205 (27.4)	NA	NA
Steroids	30 (4.0)	NA	NA
Caffeine	2 (0.3)	NA	NA
EBP success rate			
First EBP (33 articles, 1758 patients)	1062 (60.4)	64 (56-72)	93.0
Nontargeted EBP (10 articles, 264 patients)	177 (67.1)	69 (61-76)	34.9
Targeted EBP (14 articles, 816 patients)	544 (66.7)	70 (59-80)	90.5
Small EBP, <20 mL (12 articles, 680 patients)	466 (68.5)	66 (55-77)	90.3
Large EBP, ≥20 mL (4 articles, 169 patients)	139 (82.3)	77 (63-91)	69.2

Abbreviations: EBP, epidural blood patch; NA, not applicable.

^a^ Other less common treatments included surgical repair of cerebrospinal fluid defects, surgical repair of meningeal diverticula, surgical repair of cerebrospinal fluid venous fistulas, evacuation of subdural collections, and dural reduction surgery.

Epidural blood patches were the treatment most commonly offered to patients failing conservative treatment, and the first EBP was reported to be successful (clinical improvement without need for further intervention) in 64% (95% CI, 56%-72%; *I^2^* = 93.0) of patients (Table 2). An analysis of the outcomes of EBPs stratified by targeted and nontargeted EBPs demonstrated similar proportions of successful results for the targeted and nontargeted techniques. Large EBPs (>20 mL) had a higher success rate than small EBPs (eFigure 21 in the Supplement). No serious adverse events were reported after EBP treatments. The minor transient adverse events included back pain, radicular pain, tinnitus, paraesthesia, numbness, bradycardia, and dizziness.^33,34,35^ Spread of autologous blood in the subarachnoid space has been reported as a complication of EBP occurring in 8.5% of procedures.^36^ This event has not been associated with any neurologic sequela and has been reported to cause the following transient (mostly intraprocedural) symptoms: palpitation, nausea, and headache.^34,36^

Surgical repair of dural defects, meningeal diverticula, or CSF-venous fistulas was less frequently performed.^37,38,39,40^ Wang et al^40^ recently reported objective headache severity improvement in a group of 20 patients treated with surgical ligation of CSF-venous fistulas. Dural reduction surgery was not performed in any of the selected articles but was proposed as a potential surgical treatment for SIH by Schievink et al^41^ in 2009. The incidence of rebound intracranial hypertension after treatment of SIH (EBP, percutaneous, or microsurgical treatment) has been reported to be between 7% and 27.4%.^42,43^

## Discussion

This systematic review and meta-analysis provides a comprehensive summary of the available evidence on demographics, clinical presentation, investigation findings, and treatment outcomes in patients with SIH. This review highlighted a certain variability in the clinical presentation of SIH. Starting with the demographic characteristics, SIH can occur at any age (range, 2-88 years) and in both sexes with a predilection for female individuals (63%). The variability of SIH is also demonstrated by the great diversity of signs and symptoms at presentation (Table 1). As expected, headache is the most common symptom. However, the orthostatic headache, once believed to be an essential characteristic of SIH, is not invariably present. In this review, 8% of patients had a nonorthostatic headache and 3% did not experience headaches. These percentages are likely to be underestimations as most authors used the *ICHD-2* diagnostic criteria that include the presence of orthostatic headache as an essential criterion.^10^ Therefore, a diagnosis of SIH should not be excluded based on the absence of orthostatic headache. The most recent versions of the SIH diagnostic criteria (*ICHD-3 beta* and *ICHD-3*) do not use this criterion; therefore, future studies could clarify what is the true frequency of nonorthostatic headache in SIH.^1,11^ The more detailed headache description offered by some authors demonstrated a common pattern in the SIH headache phenotype: it is frequently occipital, frontal, or diffuse (Table 1). The occipital location may be one possible pointer toward this condition.

Among all investigations examined in this review, brain MRI was the most sensitive in detecting signs of SIH. In particular, diffuse pachymeningeal enhancement was detected in 73% of patients with SIH. Brain MRI has also the advantage of being readily available, noninvasive, and easy to perform. Brain MRI can be particularly useful in confirming diagnosis of SIH in patients with normal CSF pressure and CSF leaks that are difficult to identify with spinal imaging. However, it should be borne in mind that 19% of patients with SIH have normal brain MRI findings; therefore, a normal brain MRI result does not exclude SIH. While useful for confirming a diagnosis of SIH, brain MRIs do not give any information regarding the CSF leak location and need to be followed up by spinal investigations if targeted treatment is planned. Considering the general availability, the lack of radiation, the sensitivity, and the experience built over the last few decades, brain MRI with intravenous contrast should be offered as initial imaging test for the investigation of SIH.

One of the challenges in the management of patients with SIH comes from the inability to clearly identify a CSF leak with the currently available spinal investigation methods. In a significant proportion of patients who have a convincing clinical history for SIH, a CSF leak cannot be demonstrated radiologically. According to the results of this systematic review, spinal imaging techniques (spinal MRI, computed tomography myelograms, radionuclide cisternography, MR myelogram, and DSM) can identify evidence of extradural CSF leak in only 48% to 67% of patients (Figure 2A). Moreover, when a leak is identified with these techniques, its exact location can often remain unknown (Figure 2B). Digital subtraction myelography and MR myelography with the unconventional use of intrathecal gadolinium had the highest sensitivity for identifying the exact leak site (100% and 75.5%, respectively). However, the number of cases investigated with these techniques and reported in the literature is very small (133 and 87 patients, respectively). Most importantly, MR myelography with intrathecal gadolinium is not commonly available, intrathecal gadolinium has been reported to induce neurotoxicity (especially at higher doses), and more recent evidence (published after the systematic review period) suggest nonsuperiority of this technique compared to MR myelography without intrathecal gadolinium.^44,45^ Dynamic computed tomography myelography has been reported to facilitate the localization of fast CSF leak; however, further studies will be needed to confirm its role in the diagnosis of SIH.^17,30,31^ In view of the availability, safety, and the sensitivity (comparable with other spinal investigations), spinal MRI with contrast should probably be preferred as first step spinal imaging to other more invasive spinal investigations involving the need for spinal punctures and/or the exposure to high doses of radiation. Digital subtraction myelography could instead play an important role in the identification of the exact leak site and guide targeted treatment; however, larger studies confirming the utility of this investigation are required.

Low lumbar puncture CSF pressure (<60 mm H_2_0) was initially considered an essential feature of SIH (giving the name to this condition) and has been part of the *ICHD* diagnostic criteria since 2004. As initially reported by Mokri et al^46^ and later confirmed by Kranz et al,^32^ this systematic review also confirms that this finding is inconsistent and that many patients with SIH have normal (and occasionally high) lumbar puncture opening pressure. Our results show that CSF pressure is normal in 32% of patients with SIH. It should be noted that the presence of low CSF opening pressure in many diagnostic criteria for SIH could have led clinicians to exclude this diagnosis in patients with normal pressure. Therefore, the number of patients with normal or high pressure may actually be much higher than currently reported. The reasons why 32% of patients with SIH have normal CSF opening pressure on lumbar puncture might be related to the inadequate methods of measurement or to the actual absence of a low CSF pressure state. Lumbar puncture opening pressure is a snapshot method of measurement, is not reflective of the intracranial pressure in the upright position, and does not offer any information regarding the CSF dynamics during a postural change. The correlation between SIH and connective tissue disorders supports the hypothesis of a dural compliance disorder as the main cause for this syndrome.^16,20,22^ The finding of a high CSF pressure in 3% of patients also raises the possibility that some of these patients might actually be affected by idiopathic intracranial hypertension with a paradoxical presentation, although the only mildly elevated CSF pressures points away from this notion. Alternatively, the presence of an initially raised CSF pressure could predispose the patient to the onset of a spontaneous CSF leak at a weak point of the dura. Lumbar punctures have good sensitivity (67%) and can support the diagnosis of SIH; however, a normal opening pressure does not exclude this disorder.

A significant proportion of patients (28%) successfully respond to conservative treatment measures (Table 2). Based on this finding, it would be beneficial to attempt conservative treatment before EBP in every patient with SIH, but further studies will need to clarify the best type and duration of conservative treatment. One EBP was effective in 64% of patients (Table 2). Improvement after EBP was one of the diagnostic criteria included in the *ICHD-II* classification; therefore, the proportion of successful outcomes reported in this systematic review could be an overestimate. According to the literature, EBPs also have a very safe profile, with only transient minor complications. When comparing different EBP techniques, large EBPs (>20 mL) gave successful outcomes in a higher proportion of patients than small EBPs (Figure 3). This finding is in line with the results of a previous study by Wu et al.^24^ On the other hand, the use of targeted EBPs gave similar success rates compared with nontargeted EBPs. Randomized clinical trials will be required to confirm the superiority of large EBPs and investigate the potential difference between targeted and nontargeted EBPs. Based on the results of this review, large nontargeted EBP could be attempted in patients with SIH who do not improve with conservative treatment.

The results of this study suggest that the absence of orthostatic headache, normal imaging findings, or normal lumbar puncture opening pressures can occur in SIH; therefore, this diagnosis cannot be excluded in patients who do not present with all the typical features of this disorder. We propose that brain MRI and spine MRI with contrast could be performed as first-line investigations in patients with clinical suspicion of SIH. While a lumbar puncture could be offered to patients with a clinical picture suggestive of SIH but inconclusive first-line imaging, it needs to be undertaken with caution bearing in mind that the sensitivity of this investigation is relatively low (67%) and there is a risk of worsening SIH. Treatment with EBPs could be attempted early, even if the exact leak location is unknown. Second-line spinal imaging (eg, DSM or MR myelography with intrathecal gadolinium) could be offered to patients who do not respond to EBP and require targeted treatment (EBP or surgical).

### Limitations

The limitations of this systematic review are related to the heterogeneous nature of the SIH studies available in the literature, the lack of randomized clinical trials, and the lack of continuity in the diagnostic criteria used throughout the past decades. This heterogeneity is clearly reflected in the results of the various meta-analyses often showing an *I^2^* more than 75% and was addressed through the use of random effect analyses. Future research should aim at investigating the exact etiology of this condition, as well as improving the diagnostic and treatment techniques for SIH through large randomized clinical studies. Despite its limitations, this study offers a comprehensive and objective summary of the evidence on SIH that could be useful in guiding clinical practice and future research.

## Conclusions

This systematic review and meta-analysis summarizes the clinical presentation, investigation findings, and treatment outcomes of SIH based on the reports of 144 articles. Absence of orthostatic headache, normal imaging findings, or normal lumbar puncture opening pressure should not exclude a diagnosis of SIH. A single EBP was successful in 64% of patients. While this meta-analysis suggests that large EBPs have successful outcomes in a higher proportion of patients compared with small EBPs, this requires further validation. Large randomized clinical trials will be required to define the best management for SIH.

## Notes

Supplement. eTable 1. Variables included in the systematic review eTable 2. Inclusion criteria for meta-analyses eTable 3. List of selected studies and inclusion for meta-analyses eFigure 1. Flowchart of the selection process for articles eFigure 2. Age meta-analysis (mean years) eFigure 3. Sex meta-analysis (proportion of female patients) eFigure 4. Symptoms duration meta-analysis (mean days) eFigure 5. Meta-analysis of proportions of patients with and without headache eFigure 6. Meta-analysis of proportions of patients with and without orthostatic headache eFigure 7. Meta-analysis of proportions of headache location eFigure 8. Meta-analyses of proportions of signs and symptoms eFigure 9. Meta-analyses of proportions of brain MRI findings eFigure 10. Meta-analysis of proportions of positive findings in spinal MRI eFigure 11. Meta-analysis of proportions of positive findings in CT myelography (CTM) eFigure 12. Meta-analysis of proportions of positive findings in MR myelography eFigure 13. Meta-analysis of proportions of positive findings in MR myelography with intrathecal gadolinium eFigure 14. Meta-analysis of proportions of positive findings in radionuclide cisternography eFigure 15. Meta-analysis of proportions of positive findings in digital subtraction myelography eFigure 16. Meta-analysis of proportions of positive findings in digital subtraction myelography performed in lateral decubitus position eFigure 17. Meta-analysis of proportions of leak location eFigure 18. Meta-analysis of proportions of lumbar puncture opening pressure eFigure 19. Meta-analysis of proportions of successful and unsuccessful conservative treatment eFigure 20. Meta-analysis of proportions of successful EBP eFigure 21. Epidural Blood Patches (EBPs) outcomes. Pooled estimates of proportions (95% CI) of successful EBP treatment stratified by EBP technique (non-targeted/targeted, small/large) eReferences
