# Supplementary material for: Clinical Presentation, Investigation Findings, and Treatment Outcomes of Spontaneous Intracranial Hypotension Syndrome: A Systematic Review and Meta-analysis
Source: JAMA Neurol. 2021 Jan 4;78(3):1–9. doi: 10.1001/jamaneurol.2020.4799 (PMC7783594; doi:10.1001/jamaneurol.2020.4799)
Supplement: Supplement. — eTable 1. Variables included in the systematic review eTable 2. Inclusion criteria for meta-analyses eTable 3. List of selected studies and inclusion for meta-analyses eFigure 1. Flowchart of the selection process for articles eFigure 2. Age meta-analysis (mean years) eFigure 3. Sex meta-analysis (proportion of female patients) eFigure 4. Symptoms duration meta-analysis (mean days) eFigure 5. Meta-analysis of proportions of patients with and without headache eFigure 6. Meta-analysis of proportions of patients with and without orthostatic headache eFigure 7. Meta-analysis of proportions of headache location eFigure 8. Meta-analyses of proportions of signs and symptoms eFigure 9. Meta-analyses of proportions of brain MRI findings eFigure 10. Meta-analysis of proportions of positive findings in spinal MRI eFigure 11. Meta-analysis of proportions of positive findings in CT myelography (CTM) eFigure 12. Meta-analysis of proportions of positive findings in MR myelography eFigure 13. Meta-analysis of proportions of positive findings in MR myelography with intrathecal gadolinium eFigure 14. Meta-analysis of proportions of positive findings in radionuclide cisternography eFigure 15. Meta-analysis of proportions of positive findings in digital subtraction myelography eFigure 16. Meta-analysis of proportions of positive findings in digital subtraction myelography performed in lateral decubitus position eFigure 17. Meta-analysis of proportions of leak location eFigure 18. Meta-analysis of proportions of lumbar puncture opening pressure eFigure 19. Meta-analysis of proportions of successful and unsuccessful conservative treatment eFigure 20. Meta-analysis of proportions of successful EBP eFigure 21. Epidural Blood Patches (EBPs) outcomes. Pooled estimates of proportions (95% CI) of successful EBP treatment stratified by EBP technique (non-targeted/targeted, small/large) eReferences [file jamaneurol-e204799-s001.pdf]

Supplemental Online Content

D'Antona L, Jaime Merchan MA, Vassiliou A, et al. Clinical presentation, investigation findings, and treatment outcomes of spontaneous intracranial hypotension syndrome: a systematic review and meta-analysis. *JAMA Neurol*. Published online January 4, 2021. doi:10.1001/jamaneurol.2020.4799

eTable 1. Variables included in the systematic review
eTable 2. Inclusion criteria for meta-analyses
eTable 3. List of selected studies and inclusion for meta-analyses
eFigure 1. Flowchart of the selection process for articles
eFigure 2. Age meta-analysis (mean years)
eFigure 3. Sex meta-analysis (proportion of female patients)
eFigure 4. Symptoms duration meta-analysis (mean days)
eFigure 5. Meta-analysis of proportions of patients with and without headache
eFigure 6. Meta-analysis of proportions of patients with and without orthostatic headache
eFigure 7. Meta-analysis of proportions of headache location
eFigure 8. Meta-analyses of proportions of signs and symptoms
eFigure 9. Meta-analyses of proportions of brain MRI findings
eFigure 10. Meta-analysis of proportions of positive findings in spinal MRI
eFigure 11. Meta-analysis of proportions of positive findings in CT myelography (CTM)
eFigure 12. Meta-analysis of proportions of positive findings in MR myelography
eFigure 13. Meta-analysis of proportions of positive findings in MR myelography with intrathecal gadolinium
eFigure 14. Meta-analysis of proportions of positive findings in radionuclide cisternography
eFigure 15. Meta-analysis of proportions of positive findings in digital subtraction myelography
eFigure 16. Meta-analysis of proportions of positive findings in digital subtraction myelography performed in lateral decubitus position
eFigure 17. Meta-analysis of proportions of leak location
eFigure 18. Meta-analysis of proportions of lumbar puncture opening pressure
eFigure 19. Meta-analysis of proportions of successful and unsuccessful conservative treatment
eFigure 20. Meta-analysis of proportions of successful EBP
eFigure 21. Epidural Blood Patches (EBPs) outcomes. Pooled estimates of proportions (95% CI) of successful EBP treatment stratified by EBP technique (non-targeted/targeted, small/large)
eReferences

This supplemental material has been provided by the authors to give readers additional information about their work.

© 2021 D'Antona et al. *JAMA Neurology.*

eTable 1. Variables included in the systematic review

Domain	Variables
Study characteristics	Authors, affiliations, SIH diagnostic criteria, study design (prospective/retrospective, observational/interventional), number of patients.
Demographics	Age (mean, SD, range), sex (male/female).
Risk factors	Patients with connective tissue disorders, spinal pathologies, other risk factors.
Clinical presentation	Symptoms duration (mean, SD, range), headache (n. patients and characteristics), nausea/vomiting, neck pain/stiffness, hearing impairment, tinnitus, other ear signs/symptoms, dizziness, vertigo, photophobia, diplopia, other visual signs/symptoms, reduced level of consciousness, cognitive disturbances, back pain, movement disturbances, other signs/symptoms.
Brain MRI	N. of investigations performed, diffuse pachymeningeal enhancement, subdural collections, brain sagging, pituitary gland enlargement, venous engorgement, slit ventricles, tonsillar descent, normal brain MRI appearances, other findings.
Spinal imaging	For each type of spinal investigation: n. of investigations performed, n. of extradural CSF detection, type of leak detection (specific leak site, broad area, unspecified). Type of investigations: spine MRI, CT myelogram, radionuclide cisternography, MR myelography (with and without intrathecal gadolinium), other spinal investigations.
CSF leak location	N. of leaks identified, leaks location (cervical, cervico-thoracic, thoracic, thoraco-lumbar, lumbar, lumbo-sacral, sacral, multiple leaks).
CSF pressure	N. of lumbar punctures (or CSF pressure measurements), pressure findings: low (<60 mmH$_2$0), normal (60-200 mmH$_2$0), high (>200 mmH$_2$0).
Treatments and outcomes	Conservative treatment: n. of patients treated, duration of treatment, outcomes, type (bed rest, hydration, caffeine, analgesia, steroids);<br>EBP: n. of patients treated, duration of treatment, outcomes, n. of EBPs, type (targeted, nontargeted, large(>20ml), small);<br>Other treatments.
CSF: Cerebrospinal Fluid; EBP: Epidural Blood Patch; SD: Standard Deviation.	

© 2021 D'Antona et al. *JAMA Neurology.*

eTable 2. Inclusion criteria for meta-analyses

Metanalyses	Inclusion criteria for metanalyses
Age	Mean age and SD specified.
Sex	N. of males/females specified.
Symptoms duration	Mean duration and SD specified.
Sign/Symptoms	For each sign/symptom: n. of patients with the sign/symptom specified.
Brain MRI	N. of investigations, type of positive findings and proportion of positive findings specified.
Spinal imaging	N. of investigations, type of investigations and proportion of positive findings (extradural CSF) specified.
CSF pressure	N. of investigations and findings (n. low pressure, normal pressure and high pressure) specified. Studies in which all patients had low CSF opening pressure were excluded.
Outcomes of conservative treatment	N. of patients who received treatment and n. good outcomes specified. Studies in which all (or none) of the patients had good outcomes were excluded.
Outcomes of first EBP	N. of patients who received treatment and n. good outcomes after 1st EBP specified. Studies in which all (or none) of the patients had good outcomes were excluded.
Outcomes of nontargeted/targeted EBP	N. of patients who received treatment, type of EBP (nontargeted or targeted) and n. good outcomes after 1st EBP specified. Studies in which all (or none) of the patients had good outcomes were excluded.
Outcomes of small/large EBP	N. of patients who received treatment, type of EBP (small or large) and n. good outcomes after 1st EBP specified. Studies in which all (or none) of the patients had good outcomes were excluded.
All	Studies with 0% or 100% proportion of findings were excluded.<br>For each meta-analysis, if more than one study from the same author met the inclusion criteria, only the study reporting the largest number of patients was included.

CSF: Cerebrospinal Fluid; EBP: Epidural Blood Patch; SD: Standard Deviation.

© 2021 D'Antona et al. *JAMA Neurology.*

eTable 3. List of selected studies and inclusion for meta-analyses

Reference	Author (year)	Quality grading[a]	Patients (n)	Meta-analyses
**(1)**	Cebeci et al (2020)	Fair	20	Age, sex, other symptoms, MRMG, leak location
**(2)**	Davies et al (2020)	Good	86	Sex, other symptoms, conservative treatment
**(3)**	Wang et al (2020)	Good	20	
**(4)**	Dobrocky et al (2019)	Good	56	Age, sex, brain MRI
**(5)**	Li et al (2019)	Fair	40	Age, sex, cinical presentation, brain MRI, LPOP
**(6)**	Levi et al (2019)	Good	101	Age, sex, headache, other symptoms, nontargeted EBP, small EBP
**(7)**	Kim et al (2019)	Good	43	Age, sex, symptoms duration, headache, other symptoms, brain MRI
**(8)**	Schievink et al (2019)	Good	113	Age, symptoms duration, headache
**(9)**	Schievink et al (2019)	Good	49	DSM
**(10)**	Pagani-Estévez et al (2019)	Fair	202	First EBP
**(11)**	Fichtner et al (2019)	Fair	27	LPOP, leak location
**(12)**	Martin et al (2019)	Fair	94	Other symptoms, first EBP
**(13)**	Kinsman et al (2019)	Fair	144	Sex
**(14)**	Farb et al (2019)	Fair	31	Sex, brain MRI, spine MRI, DSM, leak location
**(15)**	Griffin et al (2019)	Good	92	
**(16)**	Ohtonary et al (2018)	Fair	19	Age, sex, headache, other symptoms, LPOP, leak location, conservative,
**(17)**	Murakami et al (2018)	Good	38	Age, sex
**(18)**	Lee et al (2018)	Fair	62	Age, sex, leak location, first EBP, targeted EBP
**(19)**	Schievink et al (2018)	Good	29	Conservative treatment
**(20)**	Xia et al (2018)	Good	156	Conservative treatment
**(21)**	Ahn et al (2018)	Good	116	Sex, brain MRI, spine MRI, CTM, leak location, first EBP, nontargeted EBP, targeted EBP
**(22)**	Yagi et al (2018)	Fair	37	Sex, symptoms duration, other symptoms, brain MRI, leak location, conservative, first EBP, large EBP
**(23)**	Tsai et al (2018)	Fair	27	Symptoms duration, brain MRI
**(24)**	Takai et al (2018)	Fair	11	Symptoms duration, LPOP
**(25)**	Dobrocky et al (2018)	Fair	14	
**(26)**	Clark et al (2018)	Fair	101	
**(27)**	Ferrante et al (2018)	Fair	35	
**(28)**	Beck et al (2018)	Fair	47	
**(29)**	Schievink et al (2018)	Fair	15	
**(30)**	Yao et al (2017)	Fair	206	Age, sex, symptoms duration, headache, brain MRI, LPOP
**(31)**	Wu et al (2017)	Good	150	Age, sex, symptoms duration, headache, other symptoms, brain MRI, MRM, leak location, first EBP, targeted EBP
**(32)**	Beck et al (2017)	Good	31	Other symptoms
**(33)**	He et al (2017)	Good	165	Other symptoms, CTM, MRM, leak location, conservative, first EBP, targeted EBP, small EBP
**(34)**	Choi et al (2017)	Good	95	Sex, headache, other symptoms, brain MRI, leak location, first EBP, nontargeted EBP, targeted EBP, small EBP
**(35)**	Kim et al (2017)	Fair	128	Sex, headache, headache location, other symptoms, brain MRI, first EBP
**(36)**	Kranz et al (2017)	Good	22	
**(37)**	Chen et al (2017)	Good	19	
**(38)**	Chen et al (2017)	Good	23	
**(39)**	Chen et al (2016)	Fair	45	Age, sex, headache, other symptoms, conservative, first EBP
**(40)**	So et al (2016)	Fair	34	Age, sex, symptoms duration, headache, leak location, first EBP, small EBP
**(41)**	Amemiya et al (2016)	Good	15	Age, symptoms duration, other symptoms
**(42)**	Kranz et al (2016)	Good	99	CTM
**(43)**	Schievink et al (2016)	Good	53	DSM
**(44)**	Ferrante et al (2016)	Fair	106	First EBP, large EBP

© 2021 D’Antona et al. *JAMA Neurology.*

Reference	Author (year)	Quality grading[a]	Patients (n)	Meta-analyses
**(45)**	Amrhein et al (2016)	Fair	35	First EBP, targeted EBP, small EBP
**(46)**	Fichtner et al (2016)	Good	39	Headache
**(47)**	Monteith et al (2016)	Fair	12	Headache, brain MRI, RIC
**(48)**	Schievink et al (2016)	Good	568	Sex
**(49)**	Hori et al (2016)	Good	22	Sex, brain MRI
**(50)**	Karm et al (2016)	Fair	104	Sex, brain MRI, first EBP, targeted EBP
**(51)**	Ansel et al (2016)	Fair	16	Sex, brain MRI, spine MRI, leak location, first EBP, nontargeted EBP, small EBP
**(52)**	Takahashi et al (2016)	Fair	169	Sex, headache, brain MRI, RIC, LPOP, leak location, conservative, first EBP
**(53)**	Capizzano et al (2016)	Fair	41	Sex, headache, other symptoms, brain MRI
**(54)**	Tanaka et al (2016)	Good	40	Sex, headache, other symptoms, brain MRI
**(55)**	Kim et al (2016)	Fair	140	Sex, headache, other symptoms, brain MRI, spine MRI, conservative, first EBP, small EBP
**(56)**	Choi et al (2016)	Fair	18	Sex, leak location, first EBP, targeted EBP
**(57)**	Kranz et al (2016)	Good	89	Symptoms duration
**(58)**	Ferrante et al (2016)	Good	28	Symptoms duration, headache location
**(59)**	Beck et al (2016)	Good	15	
**(60)**	Verdoorn et al (2016)	Fair	129	
**(61)**	Wan et al (2016)	Fair	15	
**(62)**	Kranz et al (2015)	Good	106	Age, sex, brain MRI, LPOP
**(63)**	Idrissi et al (2015)	Fair	24	Age, sex, headache, headache location, other symptoms, brain MRI, spine MRI, RIC, leak location, conservative, first EBP,
**(64)**	Thielen et al (2015)	Fair	14	Leak location
**(65)**	Joo et al (2015)	Fair	76	Leak location, small EBP
**(66)**	Feltracco et al (2015)	Fair	18	Sex, brain MRI, first EBP, nontargeted EBP
**(67)**	Schievink et al (2015)	Fair	13	
**(68)**	Schievink et al (2015)	Good	42	
**(69)**	Xia et al (2015)	Fair	93	
**(70)**	Yoshida et al (2014)	Fair	12	Age, sex, headache, other symptoms, brain MRI, leak location, first EBP, targeted EBP, small EBP
**(71)**	Chazen et al (2014)	Good	24	Sex, CTM, MRMG, LPOP
**(72)**	Pimienta et al (2014)	Fair	50	
**(73)**	Schievink et al (2014)	Fair	338	
**(74)**	Tung et al (2014)	Good	17	
**(75)**	Hashizume et al (2013)	Fair	29	Age, sex, other symptoms, brain MRI, RIC, LPOP, conservative, first EBP, targeted EBP
**(76)**	Schievink et al (2013)	Fair	24	Headache location
**(77)**	Hosoya et al (2013)	Fair	100	Spine MRI, MRM
**(78)**	Ferrante et al (2013)	Fair	10	
**(79)**	Franzini et al (2013)	Fair	80	
**(80)**	Kranz et al (2013)	Good	19	
**(81)**	Reinstein et al (2013)	Good	50	
**(82)**	Albes et al (2012)	Fair	26	Age, sex, CTM, leak location, first EBP, targeted EBP, large EBP
**(83)**	Hasiloglu et al (2012)	Good	25	Age, sex, headache, LPOP, leak location
**(84)**	Hashizume et al (2012)	Fair	12	Leak location
**(85)**	Akbar et al (2012)	Fair	41	MRMG
**(86)**	Luetmer et al (2012)	Fair	151	Spine MRI
**(87)**	Balkan et al (2012)	Fair	11	Symptoms duration, other symptoms, brain MRI, first EBP, targeted EBP
**(88)**	Schievink et al (2012)	Good	33	
**(89)**	Schievink et al (2012)	Fair	20	
**(90)**	Takeuchi et al (2012)	Fair	15	
**(91)**	Yoon et al (2011)	Fair	30	LPOP
**(92)**	Cho et al (2011)	Good	56	Sex, headache, brain MRI, leak location, first EBP, nontargeted EBP, targeted EBP, small EBP
**(93)**	Bonetto et al (2011)	Fair	17	Sex, symptoms duration, headache, other symptoms, brain MRI, conservative, first EBP, nontargeted EBP
**(94)**	Watanabe et al (2011)	Fair	13	Symptoms duration, headache, small EBP
**(95)**	Franzini et al (2011)	Fair	74	

© 2021 D’Antona et al. *JAMA Neurology.*

Reference	Author (year)	Quality grading[a]	Patients (n)	Meta-analyses
**(96)**	Liu et al (2011)	Good	55	
**(97)**	Franzini et al (2010)	Fair	28	Leak location
**(98)**	Medina et al (2010)	Fair	13	Sex, headache, spine MRI, first EBP
**(99)**	Ferrante et al (2010)	Fair	42	Spine MRI, MRM, RIC
**(100)**	Horikoshi et al (2010)	Fair	16	Targeted EBP
**(101)**	Adachi et al (2009)	Fair	10	Age, sex, headache, other symptoms
**(102)**	Park et al (2009)	Fair	12	Age, sex, headache, other symptoms, brain MRI, RIC, LPOP, conservative
**(103)**	Su et al (2009)	Fair	11	Age, sex, headache, other symptoms, brain MRI, spine MRI, leak location, first EBP, nontargeted EBP
**(104)**	Mea et al (2009)	Good	90	Brain MRI
**(105)**	Wang et al (2009)	Fair	19	CTM
**(106)**	Shankar et al (2009)	Good	17	Sex, brain MRI
**(107)**	Mea et al (2009)	Fair	90	
**(108)**	Watanabe et al (2008)	Fair	18	Age, headache, spine MRI, LPOP
**(109)**	Schievink et al (2008)	Fair	94	Brain MRI, LPOP
**(110)**	Yoo et al (2008)	Fair	15	MRM, RIC, first EBP
**(111)**	Albayram et al (2008)	Good	19	MRMG
**(112)**	Hyun et al (2008)	Good	30	Sex, headache, other symptoms, brain MRI, RIC, LPOP, leak location, conservative, first EBP, small EBP
**(113)**	Tomoda et al (2008)	Fair	27	Sex, other symptoms, conservative treatment, MRM, LPOP, first EBP
**(114)**	Fuh et al (2008)	Fair	53	
**(115)**	Mea et al (2008)	Fair	70	
**(116)**	Mea et al (2007)	Good	59	Sex
**(117)**	Chung et al (2007)	Fair	10	
**(118)**	Farb et al (2007)	Good	12	
**(119)**	Lai et al (2007)	Fair	40	
**(120)**	Schievink et al (2007)	Fair	11	
**(121)**	Schievink et al (2007)	Fair	80	
**(122)**	Tsai et al (2007)	Fair	17	
**(123)**	Wiesemann et al (2006)	Fair	10	Age, sex, symptoms duration, headache, other symptoms, brain MRI, LPOP, conservative
**(124)**	Chung et al (2006)	Fair	67	RIC
**(125)**	Hannerz et al (2006)	Fair	12	Sex, headache, other symptoms, brain MRI, conservative, first EBP, nontargeted EBP, large EBP
**(126)**	Cohen-Gadol et al (2006)	Fair	13	Sex, headache, other symptoms, brain MRI, leak location
**(127)**	Chung et al (2005)	Fair	53	Age, spine MRI, conservative treatment
**(128)**	Kong et al (2005)	Fair	13	Age, symptoms duration, spine MRI, CTM, RIC, LPOP, conservative
**(129)**	Schievink et al (2005)	Fair	33	Leak location
**(130)**	Schievink et al (2005)	Fair	20	
**(131)**	Ferrante et al (2004)	Fair	18	LPOP
**(132)**	Berrior et al (2004)	Fair	33	Sex, symptoms duration, headache, other symptoms, brain MRI, first EBP, nontargeted EBP
**(133)**	Ferrante et al (2004)	Fair	12	
**(134)**	Schievink et al (2004)	Good	18	
**(135)**	Schievink et al (2004)	Good	25	
**(136)**	Miyazawa et al (2003)	Fair	10	Age, headache, other symptoms, brain MRI, RIC, LPOP, leak location
**(137)**	Chen et al (2003)	Fair	13	
**(138)**	Schievink et al (2003)	Good	18	
**(139)**	Lin et al (2002)	Fair	15	LPOP
**(140)**	Schrijver et al (2002)	Good	20	
**(141)**	Chung et al (2000)	Fair	30	Headache location
**(142)**	Chen et al (1999)	Good	13	
**(143)**	Schievink et al (1998)	Good	10	
**(144)**	Schievink et al (1996)	Fair	11	Spine MRI, RIC

[a]Based on NIH Quality Assessment Tool for Case Series Studies
CTM: CT myelography, EBP: Epidural Blood Patch, LPOP: Lumbar Puncture Opening Pressure, MRM: MR Myelography, MRMG: MR myelography with intrathecal Gadolinium, RIC: Radionuclide Cisternography

© 2021 D’Antona et al. *JAMA Neurology.*

eFigure 1. Flowchart of the selection process for articles

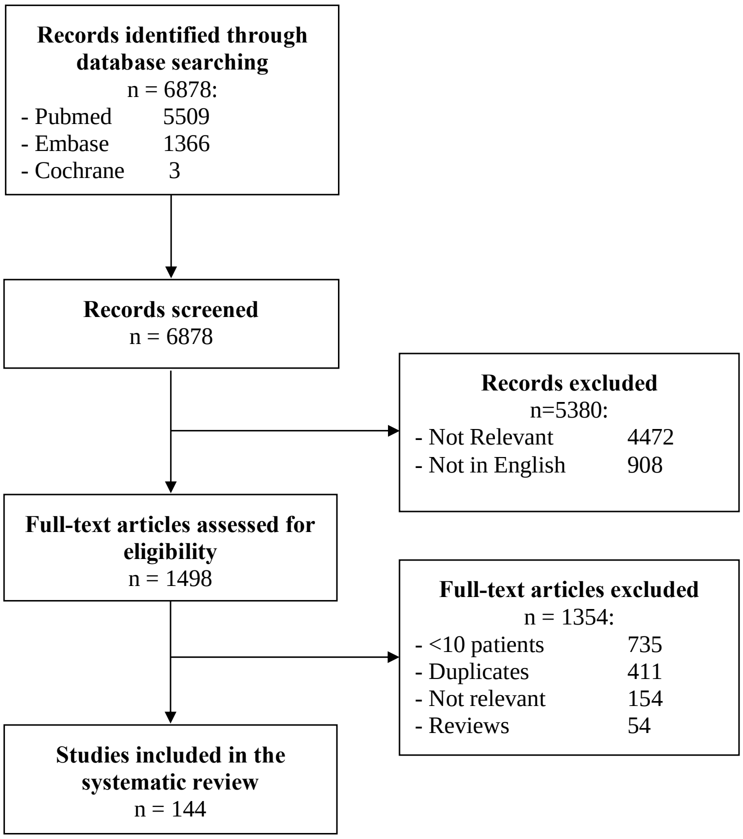

© 2021 D'Antona et al. *JAMA Neurology.*

eFigure 2. Age meta-analysis (mean years)

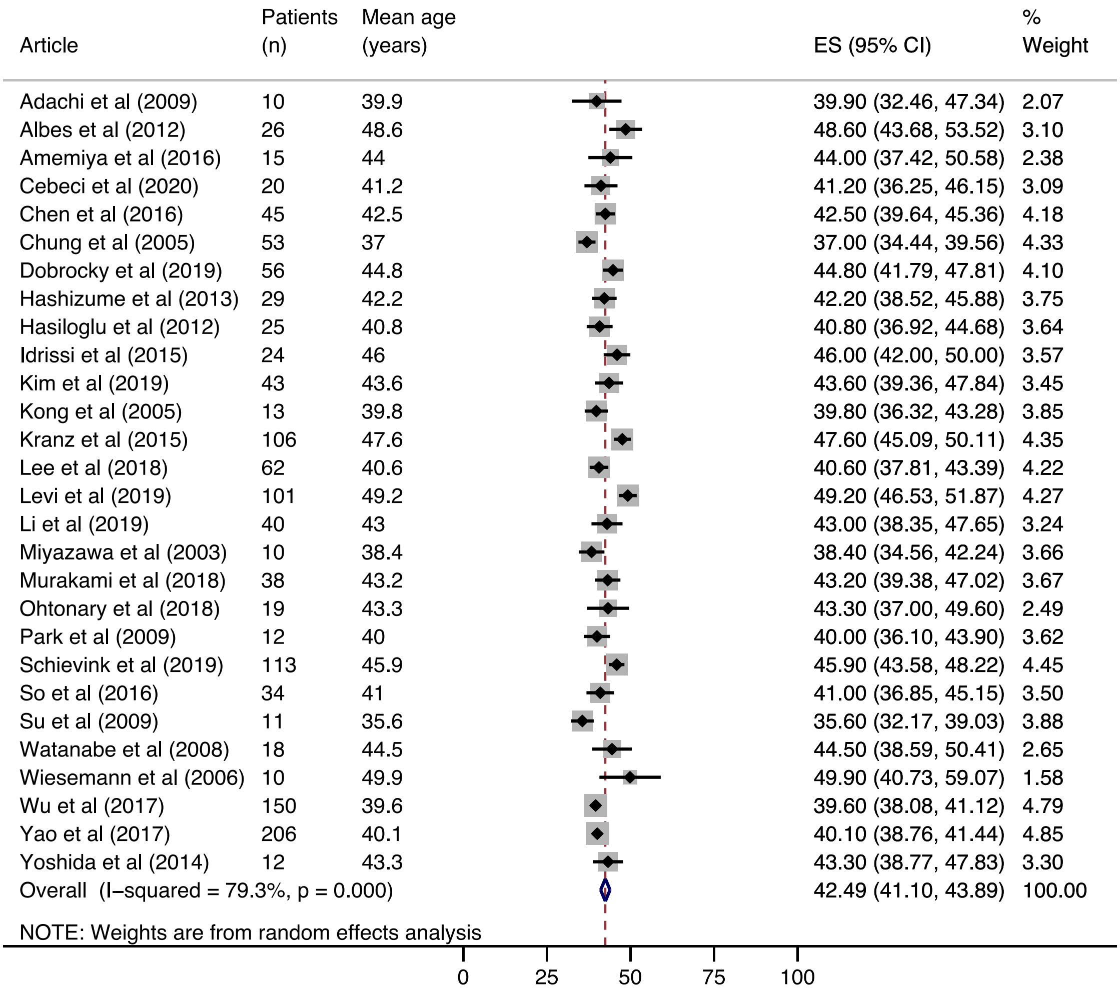

© 2021 D'Antona et al. *JAMA Neurology.*

eFigure 3. Sex meta-analysis (proportion of female patients)

Article	Patients (n)	Female (n)	ES (95% CI)	% Weight
Adachi et al (2009)	10	9	0.90 (0.60, 0.98)	1.46
Ahn et al (2018)	116	69	0.59 (0.50, 0.68)	3.11
Albes et al (2012)	26	11	0.42 (0.26, 0.61)	1.42
Ansel et al (2016)	16	11	0.69 (0.44, 0.86)	1.09
Berrior et al (2004)	33	21	0.64 (0.47, 0.78)	1.72
Bonetto et al (2011)	17	11	0.65 (0.41, 0.83)	1.09
Capizzano et al (2016)	41	26	0.63 (0.48, 0.76)	1.96
Cebeci et al (2020)	20	18	0.90 (0.70, 0.97)	2.23
Chazen et al (2014)	24	10	0.42 (0.24, 0.61)	1.35
Chen et al (2016)	45	17	0.38 (0.25, 0.52)	2.06
Cho et al (2011)	56	33	0.59 (0.46, 0.71)	2.28
Choi et al (2016)	18	9	0.50 (0.29, 0.71)	1.06
Choi et al (2017)	95	60	0.63 (0.53, 0.72)	2.93
Cohen−Gadol et al (2006)	13	9	0.69 (0.42, 0.87)	0.93
Davies et al (2020)	86	53	0.62 (0.51, 0.71)	2.80
Dobrocky et al (2019)	56	40	0.71 (0.59, 0.82)	2.48
Farb et al (2019)	31	18	0.58 (0.41, 0.74)	1.60
Feltracco et al (2015)	18	14	0.78 (0.55, 0.91)	1.40
Hannerz et al (2006)	12	9	0.75 (0.47, 0.91)	0.97
Hashizume et al (2013)	29	13	0.45 (0.28, 0.62)	1.52
Hasiloglu et al (2012)	25	12	0.48 (0.30, 0.67)	1.36
Hori et al (2016)	22	9	0.41 (0.23, 0.61)	1.27
Hyun et al (2008)	30	21	0.70 (0.52, 0.83)	1.73
Idrissi et al (2015)	24	16	0.67 (0.47, 0.82)	1.43
Karm et al (2016)	104	65	0.63 (0.53, 0.71)	3.02
Kim et al (2016)	140	86	0.61 (0.53, 0.69)	3.32
Kim et al (2017)	128	89	0.70 (0.61, 0.77)	3.34
Kim et al (2019)	43	28	0.65 (0.50, 0.78)	2.04
Kinsman et al (2019)	144	83	0.58 (0.49, 0.65)	3.32
Kranz et al (2015)	106	69	0.65 (0.56, 0.73)	3.08
Lee et al (2018)	62	41	0.66 (0.54, 0.77)	2.49
Levi et al (2019)	101	56	0.55 (0.46, 0.65)	2.93
Li et al (2019)	40	24	0.60 (0.45, 0.74)	1.90
Mea et al (2007)	59	36	0.61 (0.48, 0.72)	2.36
Medina et al (2010)	13	8	0.62 (0.36, 0.82)	0.86
Murakami et al (2018)	38	19	0.50 (0.35, 0.65)	1.80
Ohtonary et al (2018)	19	12	0.63 (0.41, 0.81)	1.17
Park et al (2009)	12	9	0.75 (0.47, 0.91)	0.97
Schievink et al (2016)	568	373	0.66 (0.62, 0.69)	4.28
Shankar et al (2009)	17	14	0.82 (0.59, 0.94)	1.51
So et al (2016)	34	22	0.65 (0.48, 0.79)	1.77
Su et al (2009)	11	7	0.64 (0.35, 0.85)	0.76
Takahashi et al (2016)	169	94	0.56 (0.48, 0.63)	3.46
Tanaka et al (2016)	40	28	0.70 (0.55, 0.82)	2.05
Tomoda et al (2008)	27	11	0.41 (0.25, 0.59)	1.47
Wiesemann et al (2006)	10	8	0.80 (0.49, 0.94)	0.95
Wu et al (2017)	150	104	0.69 (0.62, 0.76)	3.48
Yagi et al (2018)	37	24	0.65 (0.49, 0.78)	1.87
Yao et al (2017)	206	145	0.70 (0.64, 0.76)	3.76
Yoshida et al (2014)	12	6	0.50 (0.25, 0.75)	0.77
Overall (I^2 = 52.44%, p = 0.00)			0.63 (0.60, 0.66)	100.00

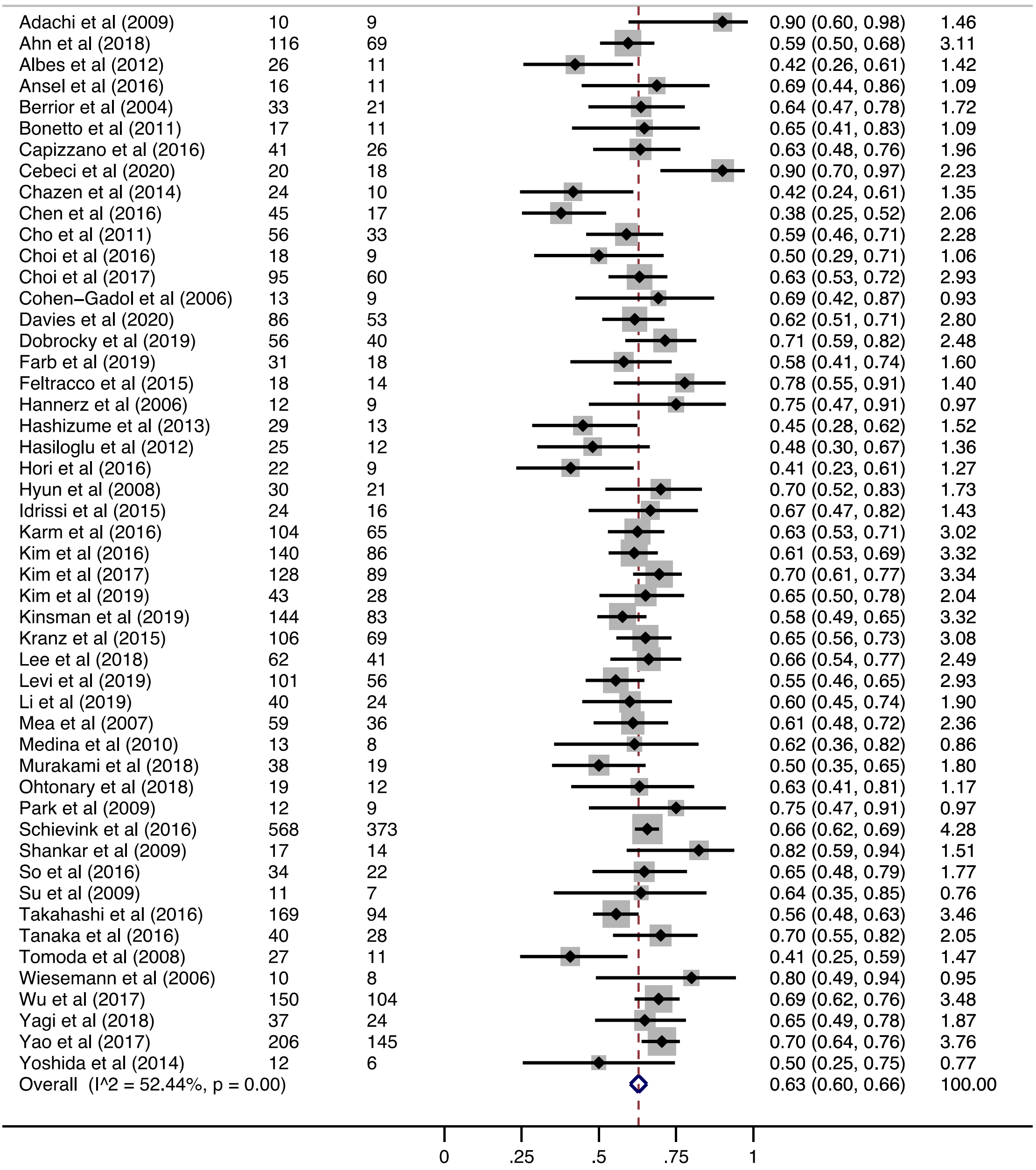

© 2021 D’Antona et al. *JAMA Neurology.*

eFigure 4. Symptoms duration meta-analysis (mean days)

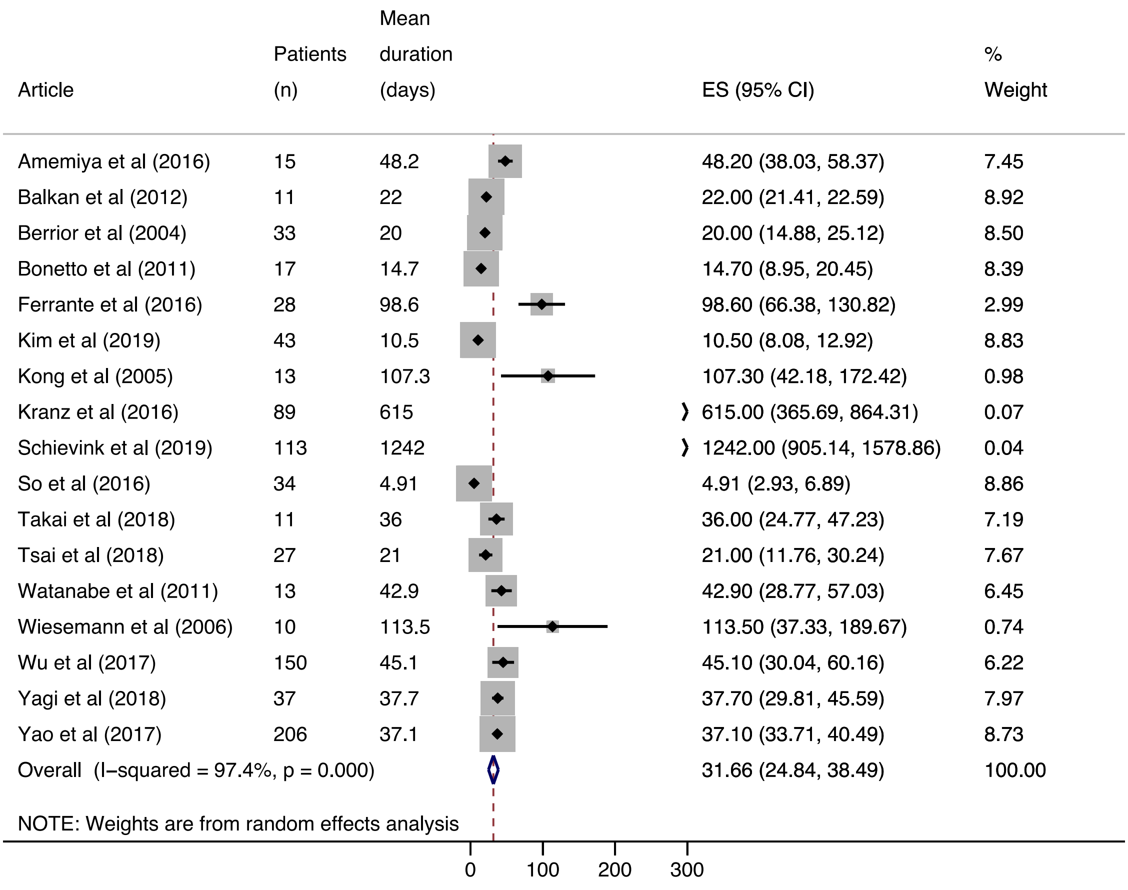

© 2021 D’Antona et al. *JAMA Neurology.*

eFigure 5a. Meta-analysis of proportions of patients with headache

Article	Patients (n)	Headache (n)	ES (95% CI)	% Weight
Capizzano et al (2016)	41	40	0.98 (0.87, 1.00)	14.61
Chen et al (2016)	45	35	0.78 (0.64, 0.87)	3.68
Cho et al (2011)	56	55	0.98 (0.91, 1.00)	19.31
Choi et al (2017)	95	94	0.99 (0.94, 1.00)	25.58
Medina et al (2010)	13	11	0.85 (0.58, 0.96)	1.52
Park et al (2009)	12	11	0.92 (0.65, 0.99)	2.33
Su et al (2009)	11	10	0.91 (0.62, 0.98)	2.00
Takahashi et al (2016)	169	167	0.99 (0.96, 1.00)	27.34
Watanabe et al (2008)	18	16	0.89 (0.67, 0.97)	2.67
Wiesemann et al (2006)	10	8	0.80 (0.49, 0.94)	0.97
Adachi et al (2009)	10	10	(Excluded)	.
Berrior et al (2004)	33	33	(Excluded)	.
Bonetto et al (2011)	17	17	(Excluded)	.
Cohen−Gadol et al (2006)	13	13	(Excluded)	.
Fichtner et al (2016)	39	39	(Excluded)	.
Hannerz et al (2006)	12	12	(Excluded)	.
Hasiloglu et al (2012)	25	25	(Excluded)	.
Hyun et al (2008)	30	30	(Excluded)	.
Idrissi et al (2015)	24	24	(Excluded)	.
Kim et al (2016)	140	140	(Excluded)	.
Kim et al (2017)	128	128	(Excluded)	.
Kim et al (2019)	43	43	(Excluded)	.
Levi et al (2019)	101	101	(Excluded)	.
Miyazawa et al (2003)	10	10	(Excluded)	.
Monteith et al (2016)	12	12	(Excluded)	.
Ohtonary et al (2018)	19	19	(Excluded)	.
Schievink et al (2019)	113	113	(Excluded)	.
So et al (2016)	34	34	(Excluded)	.
Tanaka et al (2016)	40	40	(Excluded)	.
Watanabe et al (2011)	13	13	(Excluded)	.
Wu et al (2017)	150	150	(Excluded)	.
Yao et al (2017)	206	206	(Excluded)	.
Yoshida et al (2014)	12	12	(Excluded)	.
Overall (I^2 = 52.19%, p = 0.03)			0.97 (0.94, 0.99)	100.00

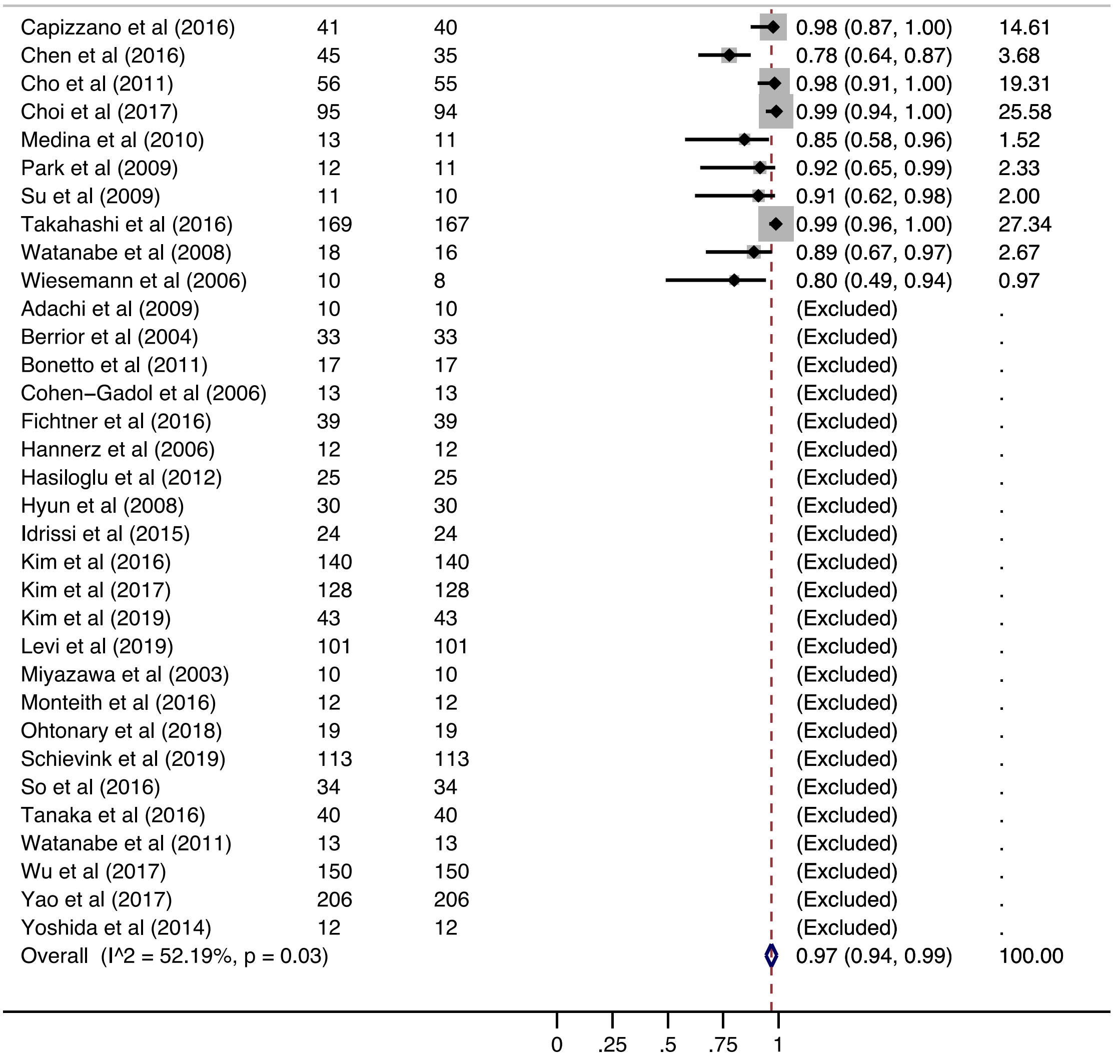

© 2021 D'Antona et al. *JAMA Neurology.*

eFigure 5b. Meta-analysis of proportions of patients without headache

Article	Patients (n)	Patients without headache (n)	ES (95% CI)	% Weight
Capizzano et al (2016)	41	1	0.02 (0.00, 0.13)	14.61
Chen et al (2016)	45	10	0.22 (0.13, 0.36)	3.68
Cho et al (2011)	56	1	0.02 (0.00, 0.09)	19.31
Choi et al (2017)	95	1	0.01 (0.00, 0.06)	25.58
Medina et al (2010)	13	2	0.15 (0.04, 0.42)	1.52
Park et al (2009)	12	1	0.08 (0.01, 0.35)	2.33
Su et al (2009)	11	1	0.09 (0.02, 0.38)	2.00
Takahashi et al (2016)	169	2	0.01 (0.00, 0.04)	27.34
Watanabe et al (2008)	18	2	0.11 (0.03, 0.33)	2.67
Wiesemann et al (2006)	10	2	0.20 (0.06, 0.51)	0.97
Adachi et al (2009)	10	0	(Excluded)	.
Berrior et al (2004)	33	0	(Excluded)	.
Bonetto et al (2011)	17	0	(Excluded)	.
Cohen–Gadol et al (2006)	13	0	(Excluded)	.
Fichtner et al (2016)	39	0	(Excluded)	.
Hannerz et al (2006)	12	0	(Excluded)	.
Hasiloglu et al (2012)	25	0	(Excluded)	.
Hyun et al (2008)	30	0	(Excluded)	.
Idrissi et al (2015)	24	0	(Excluded)	.
Kim et al (2016)	140	0	(Excluded)	.
Kim et al (2017)	128	0	(Excluded)	.
Kim et al (2019)	43	0	(Excluded)	.
Levi et al (2019)	101	0	(Excluded)	.
Miyazawa et al (2003)	10	0	(Excluded)	.
Monteith et al (2016)	12	0	(Excluded)	.
Ohtonary et al (2018)	19	0	(Excluded)	.
Schievink et al (2019)	113	0	(Excluded)	.
So et al (2016)	34	0	(Excluded)	.
Tanaka et al (2016)	40	0	(Excluded)	.
Watanabe et al (2011)	13	0	(Excluded)	.
Wu et al (2017)	150	0	(Excluded)	.
Yao et al (2017)	206	0	(Excluded)	.
Yoshida et al (2014)	12	0	(Excluded)	.
Overall (I^2 = 52.19%, p = 0.03)			0.03 (0.01, 0.06)	100.00

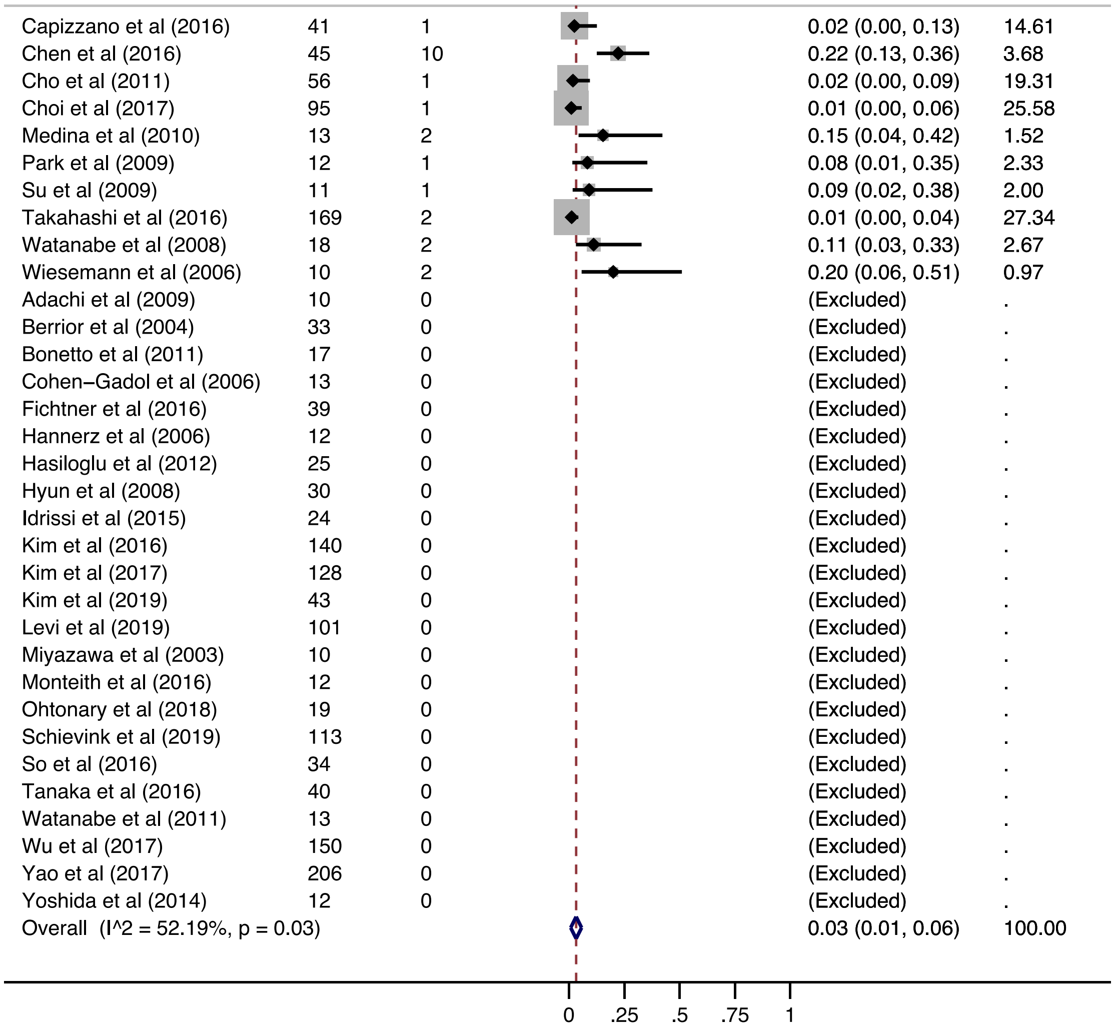

© 2021 D’Antona et al. *JAMA Neurology.*

eFigure 6a. Meta-analysis of proportions of patients with orthostatic headache

Article	Headache (n)	Orthostatic headache (n)	ES (95% CI)	% Weight
Capizzano et al (2016)	40	33	0.82 (0.68, 0.91)	9.96
Chen et al (2016)	35	24	0.69 (0.52, 0.81)	6.94
Cohen–Gadol et al (2006)	13	11	0.85 (0.58, 0.96)	4.76
Kim et al (2017)	128	127	0.99 (0.96, 1.00)	25.22
Ohtonary et al (2018)	19	13	0.68 (0.46, 0.85)	4.29
Schievink et al (2019)	113	109	0.96 (0.91, 0.99)	22.84
Takahashi et al (2016)	167	161	0.96 (0.92, 0.98)	23.72
Wiesemann et al (2006)	8	6	0.75 (0.41, 0.93)	2.27
Adachi et al (2009)	10	10	(Excluded)	.
Berrior et al (2004)	33	33	(Excluded)	.
Bonetto et al (2011)	17	17	(Excluded)	.
Cho et al (2011)	55	55	(Excluded)	.
Choi et al (2017)	94	94	(Excluded)	.
Fichtner et al (2016)	39	39	(Excluded)	.
Hannerz et al (2006)	12	12	(Excluded)	.
Hasiloglu et al (2012)	25	25	(Excluded)	.
Hyun et al (2008)	30	30	(Excluded)	.
Idrissi et al (2015)	24	24	(Excluded)	.
Kim et al (2016)	140	140	(Excluded)	.
Kim et al (2019)	43	43	(Excluded)	.
Levi et al (2019)	101	101	(Excluded)	.
Medina et al (2010)	11	11	(Excluded)	.
Miyazawa et al (2003)	10	10	(Excluded)	.
Monteith et al (2016)	12	12	(Excluded)	.
Park et al (2009)	11	11	(Excluded)	.
So et al (2016)	34	34	(Excluded)	.
Su et al (2009)	10	10	(Excluded)	.
Tanaka et al (2016)	40	40	(Excluded)	.
Watanabe et al (2008)	16	16	(Excluded)	.
Watanabe et al (2011)	13	13	(Excluded)	.
Wu et al (2017)	150	150	(Excluded)	.
Yao et al (2017)	206	206	(Excluded)	.
Yoshida et al (2014)	12	12	(Excluded)	.
Overall (I^2 = 80.95%, p = 0.00)			0.92 (0.87, 0.96)	100.00

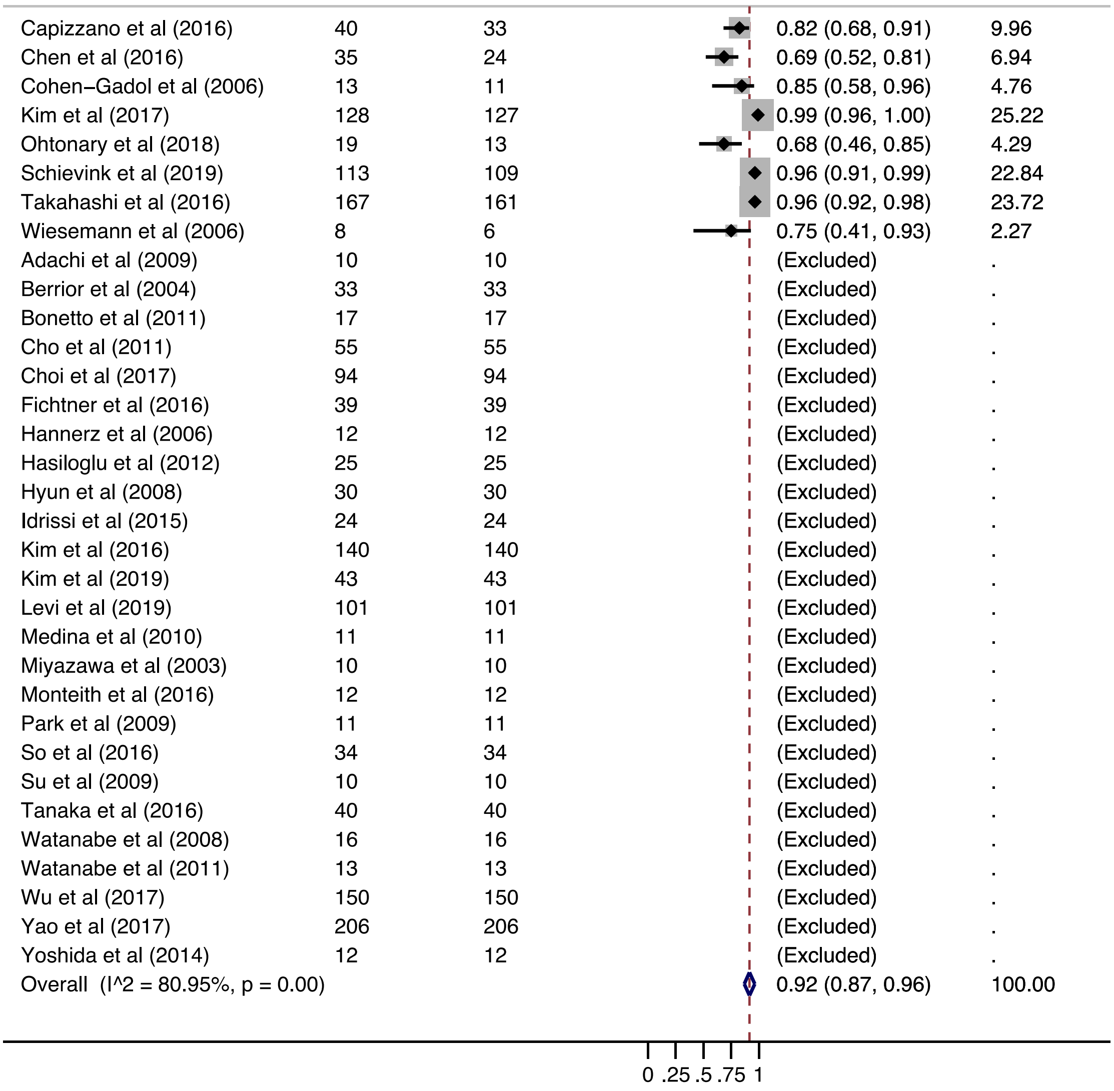

© 2021 D'Antona et al. *JAMA Neurology.*

eFigure 6b. Meta-analysis of proportions of patients with non-orthostatic headache

Article	Headache (n)	Patients with non−orthostatic headache (n)	ES (95% CI)	% Weight
Capizzano et al (2016)	40	7	0.17 (0.09, 0.32)	9.96
Chen et al (2016)	35	11	0.31 (0.19, 0.48)	6.94
Cohen−Gadol et al (2006)	13	2	0.15 (0.04, 0.42)	4.76
Kim et al (2017)	128	1	0.01 (0.00, 0.04)	25.22
Ohtonary et al (2018)	19	6	0.32 (0.15, 0.54)	4.29
Schievink et al (2019)	113	4	0.04 (0.01, 0.09)	22.84
Takahashi et al (2016)	167	6	0.04 (0.02, 0.08)	23.72
Wiesemann et al (2006)	8	2	0.25 (0.07, 0.59)	2.27
Adachi et al (2009)	10	0	(Excluded)	.
Berrior et al (2004)	33	0	(Excluded)	.
Bonetto et al (2011)	17	0	(Excluded)	.
Cho et al (2011)	55	0	(Excluded)	.
Choi et al (2017)	94	0	(Excluded)	.
Fichtner et al (2016)	39	0	(Excluded)	.
Hannerz et al (2006)	12	0	(Excluded)	.
Hasiloglu et al (2012)	25	0	(Excluded)	.
Hyun et al (2008)	30	0	(Excluded)	.
Idrissi et al (2015)	24	0	(Excluded)	.
Kim et al (2016)	140	0	(Excluded)	.
Kim et al (2019)	43	0	(Excluded)	.
Levi et al (2019)	101	0	(Excluded)	.
Medina et al (2010)	11	0	(Excluded)	.
Miyazawa et al (2003)	10	0	(Excluded)	.
Monteith et al (2016)	12	0	(Excluded)	.
Park et al (2009)	11	0	(Excluded)	.
So et al (2016)	34	0	(Excluded)	.
Su et al (2009)	10	0	(Excluded)	.
Tanaka et al (2016)	40	0	(Excluded)	.
Watanabe et al (2008)	16	0	(Excluded)	.
Watanabe et al (2011)	13	0	(Excluded)	.
Wu et al (2017)	150	0	(Excluded)	.
Yao et al (2017)	206	0	(Excluded)	.
Yoshida et al (2014)	12	0	(Excluded)	.
Overall (I^2 = 80.95%, p = 0.00)			0.08 (0.04, 0.13)	100.00

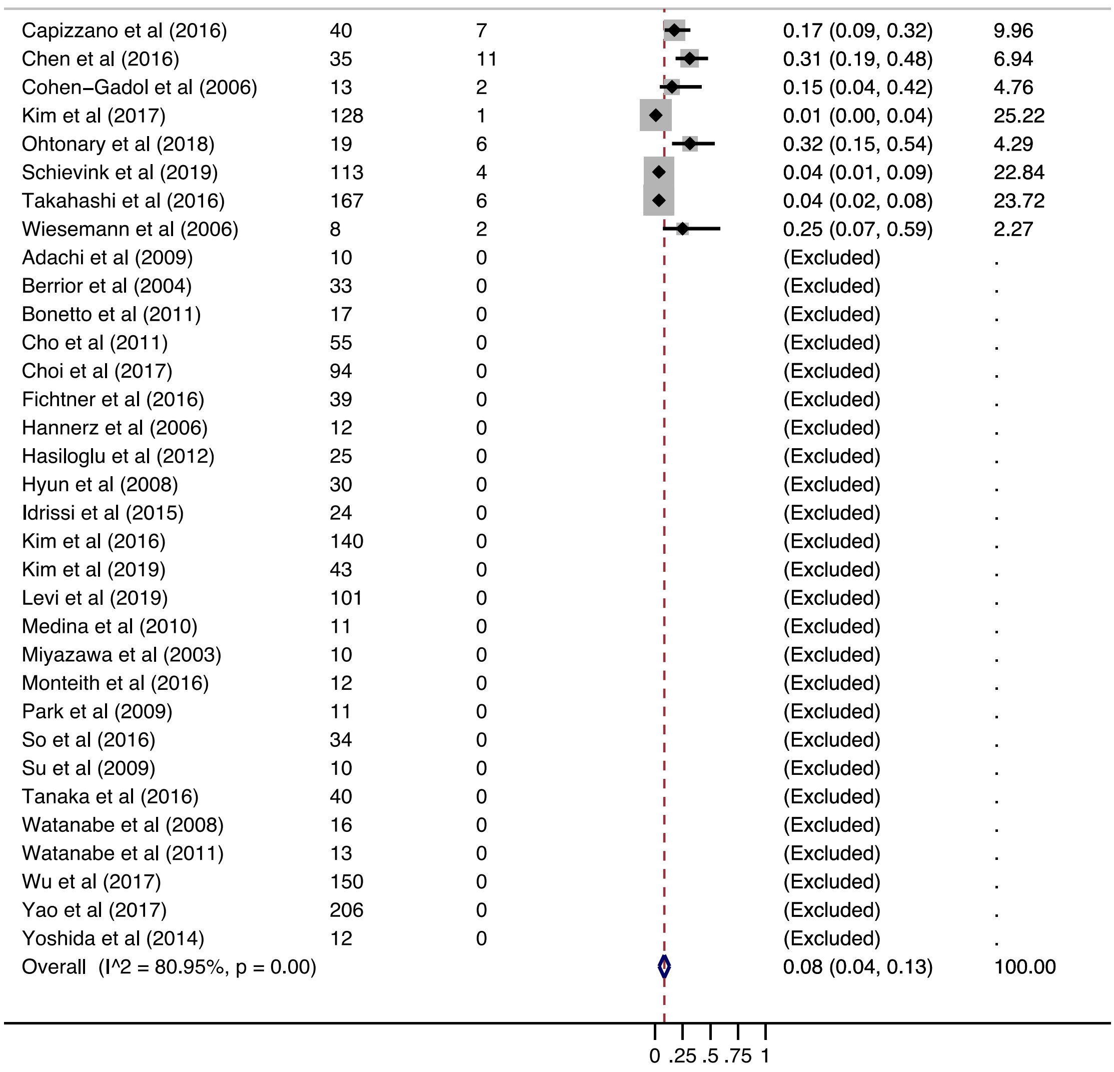

© 2021 D’Antona et al. *JAMA Neurology.*

eFigure 7a. Meta-analysis of proportions of headache location (diffuse)

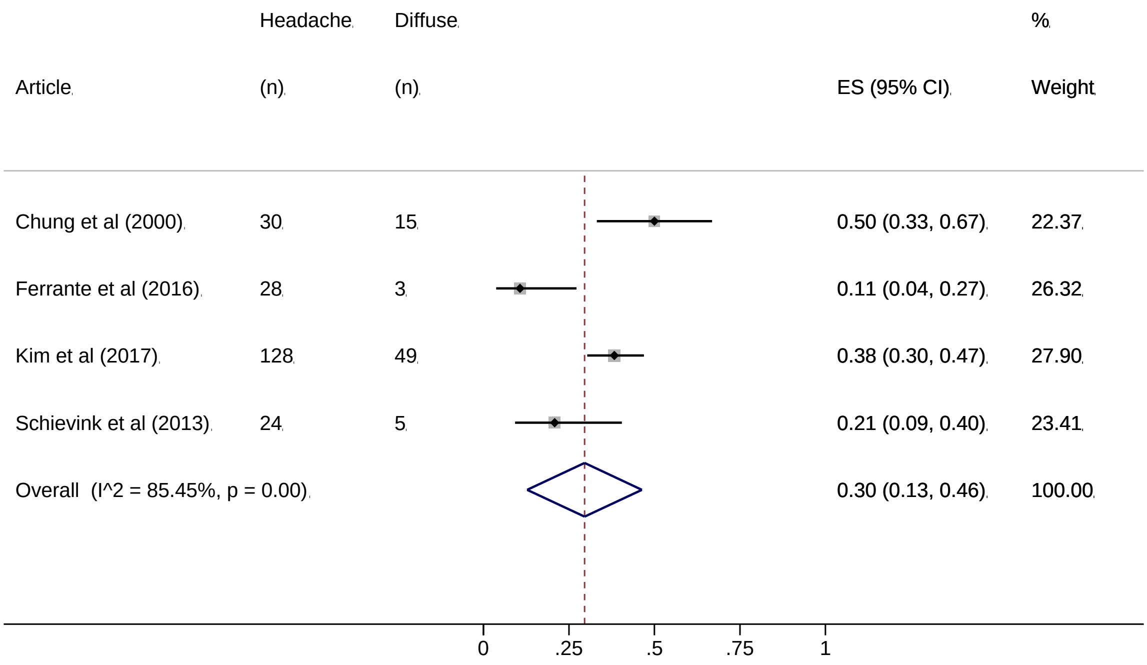

eFigure 7b. Meta-analysis of proportions of headache location (occipital)

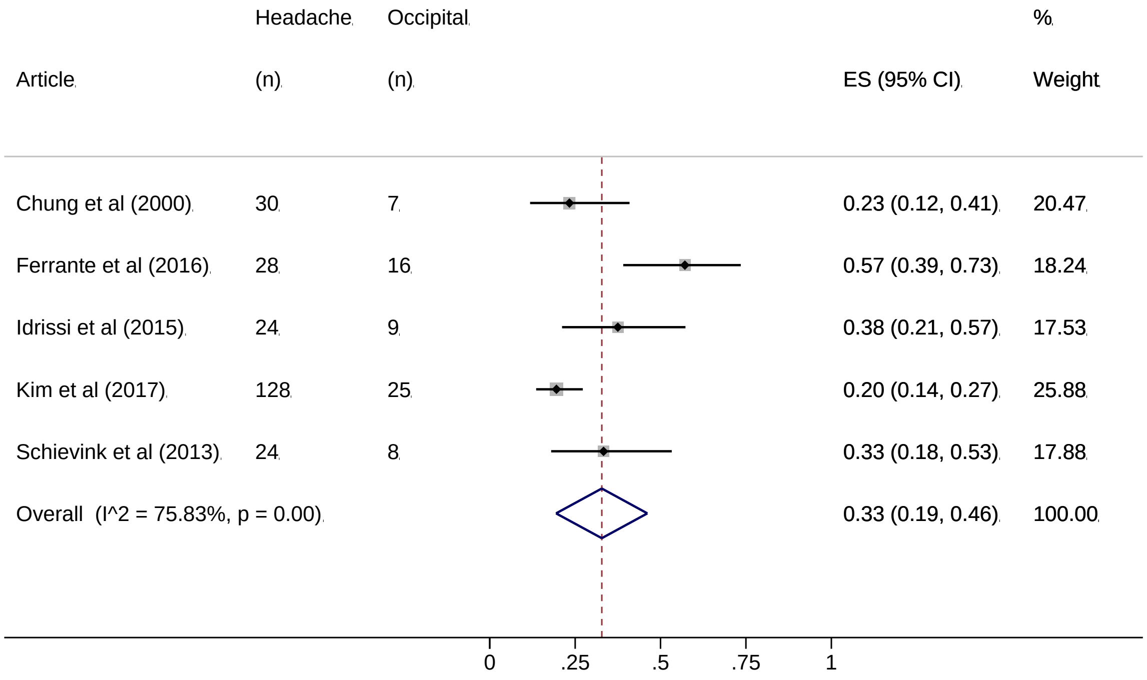

© 2021 D'Antona et al. *JAMA Neurology.*

eFigure 7c. Meta-analysis of proportions of headache location (frontal)

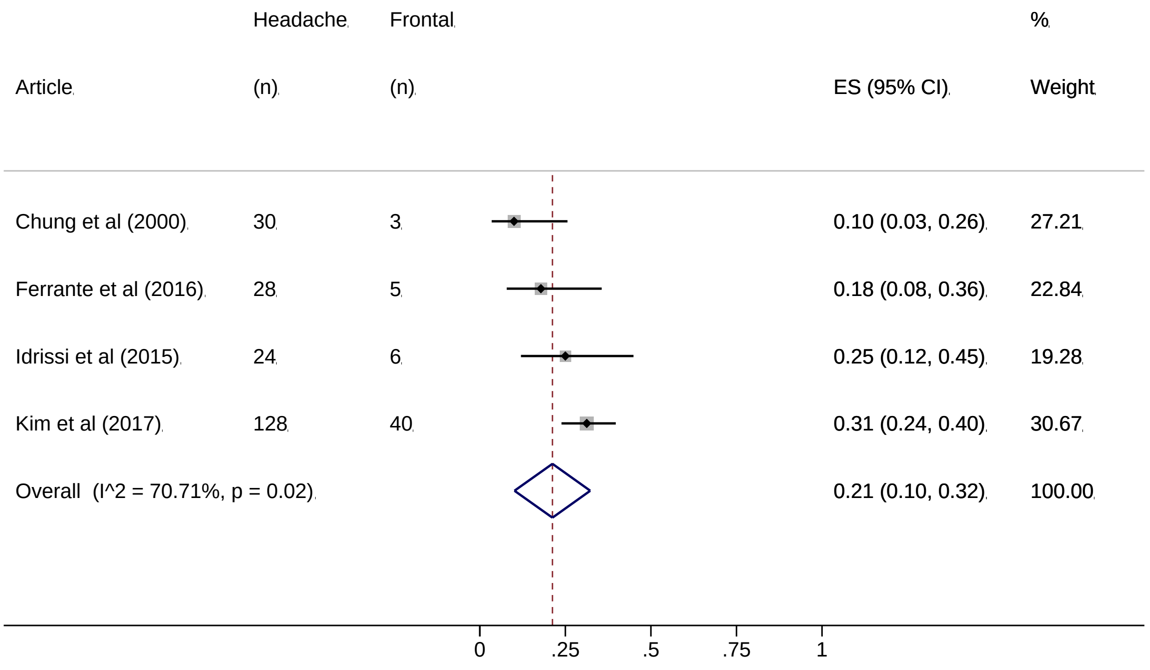

eFigure 7d. Meta-analysis of proportions of headache location (fronto-occipital)

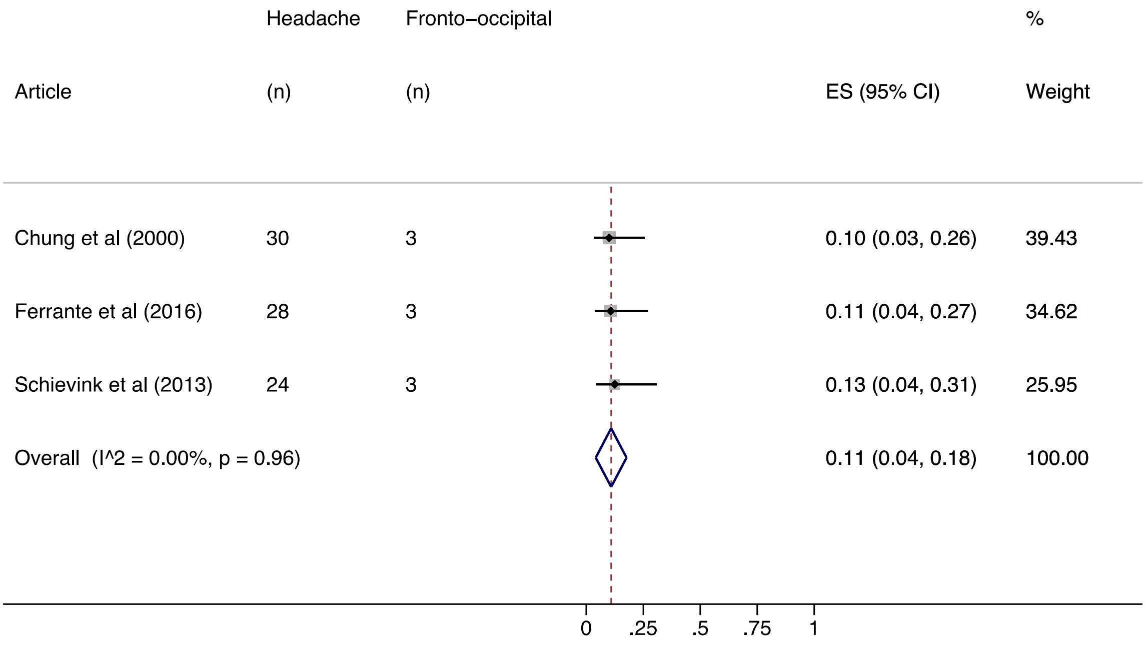

© 2021 D'Antona et al. *JAMA Neurology.*

eFigure 7e. Meta-analysis of proportions of headache location (temporal)

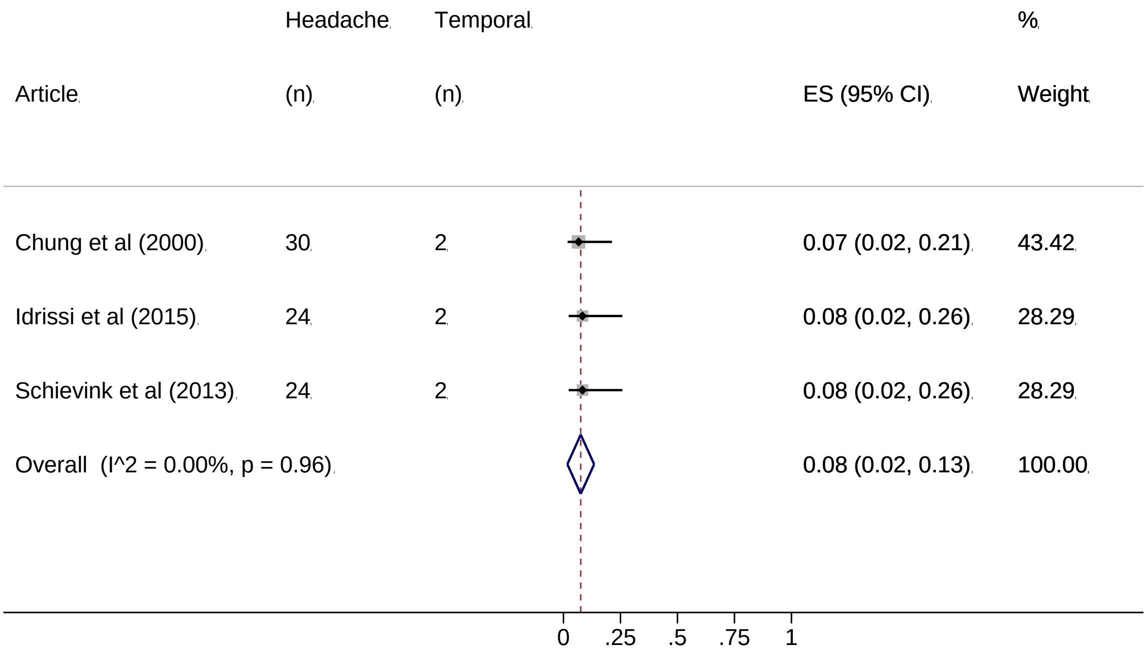

© 2021 D'Antona et al. *JAMA Neurology.*

eFigure 8a. Meta-analysis of proportions of patients with nausea/vomiting

Article	Patients (n)	Nausea/Vomiting (n)	ES (95% CI)	% Weight
Adachi et al (2009)	10	2	0.20 (0.06, 0.51)	2.93
Amemiya et al (2016)	15	10	0.67 (0.42, 0.85)	3.00
Balkan et al (2012)	11	10	0.91 (0.62, 0.98)	3.47
Beck et al (2017)	31	6	0.19 (0.09, 0.36)	3.67
Berrior et al (2004)	33	23	0.70 (0.53, 0.83)	3.56
Bonetto et al (2011)	17	11	0.65 (0.41, 0.83)	3.08
Choi et al (2017)	95	64	0.67 (0.57, 0.76)	3.92
Cohen−Gadol et al (2006)	13	9	0.69 (0.42, 0.87)	2.91
Davies et al (2020)	86	32	0.37 (0.28, 0.48)	3.88
Hashizume et al (2013)	29	24	0.83 (0.65, 0.92)	3.68
He et al (2017)	165	65	0.39 (0.32, 0.47)	4.00
Hyun et al (2008)	30	23	0.77 (0.59, 0.88)	3.60
Idrissi et al (2015)	24	18	0.75 (0.55, 0.88)	3.45
Kim et al (2016)	140	71	0.51 (0.43, 0.59)	3.97
Kim et al (2017)	128	100	0.78 (0.70, 0.84)	4.01
Kim et al (2019)	43	29	0.67 (0.53, 0.80)	3.67
Levi et al (2019)	101	53	0.52 (0.43, 0.62)	3.90
Li et al (2019)	40	25	0.63 (0.47, 0.76)	3.60
Martin et al (2019)	94	28	0.30 (0.21, 0.40)	3.93
Miyazawa et al (2003)	10	3	0.30 (0.11, 0.60)	2.69
Ohtonary et al (2018)	19	7	0.37 (0.19, 0.59)	3.15
Park et al (2009)	12	9	0.75 (0.47, 0.91)	2.95
Su et al (2009)	11	7	0.64 (0.35, 0.85)	2.69
Tanaka et al (2016)	40	10	0.25 (0.14, 0.40)	3.70
Tomoda et al (2008)	27	9	0.33 (0.19, 0.52)	3.42
Wiesemann et al (2006)	10	5	0.50 (0.24, 0.76)	2.52
Wu et al (2017)	150	110	0.73 (0.66, 0.80)	4.02
Yagi et al (2018)	37	9	0.24 (0.13, 0.40)	3.68
Yoshida et al (2014)	12	3	0.25 (0.09, 0.53)	2.95
Overall (I^2 = 89.92%, p = 0.00)			0.54 (0.46, 0.62)	100.00

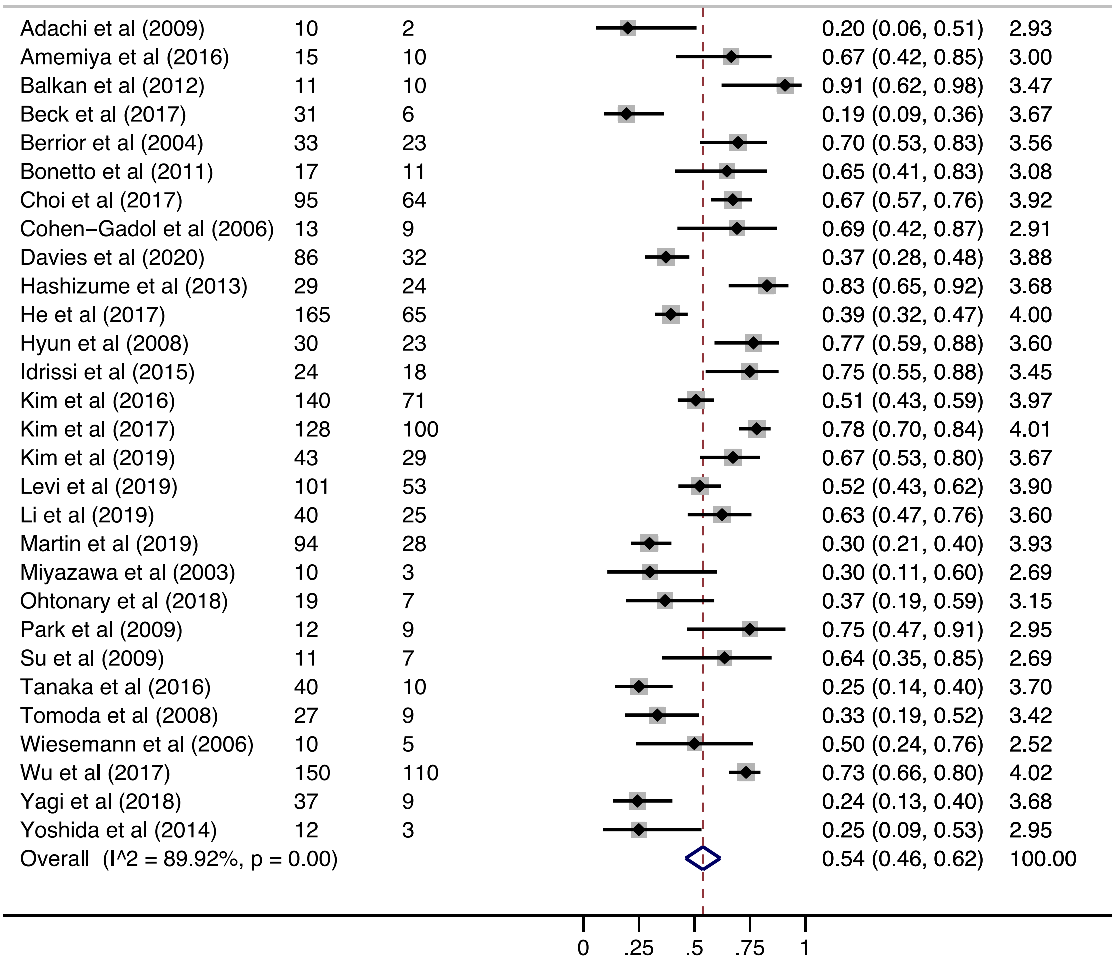

© 2021 D’Antona et al. *JAMA Neurology.*

eFigure 8b. Meta-analysis of proportions of patients with neck pain/stiffness

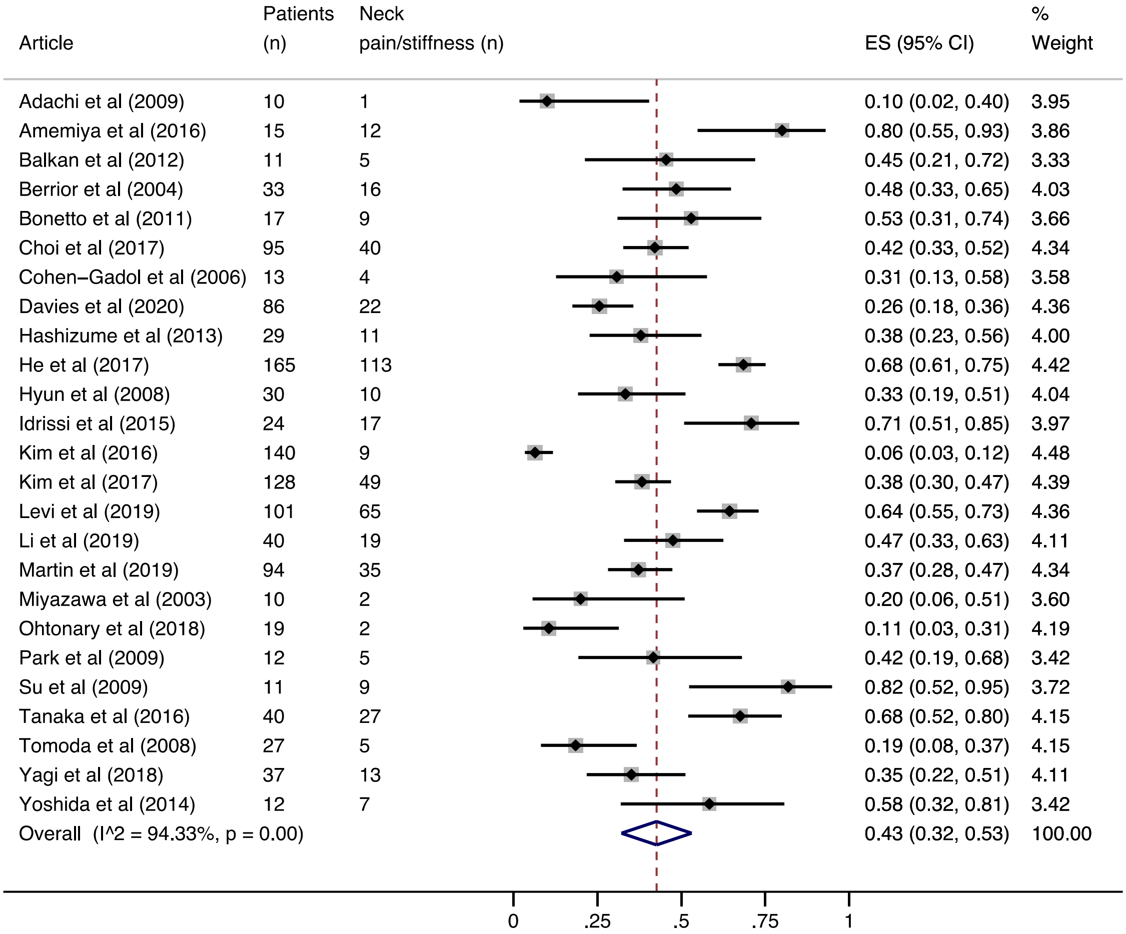

© 2021 D'Antona et al. *JAMA Neurology.*

eFigure 8c. Meta-analysis of proportions of patients with tinnitus

Article	Patients (n)	Tinnitus (n)	ES (95% CI)	% Weight
Adachi et al (2009)	10	1	0.10 (0.02, 0.40)	3.89
Amemiya et al (2016)	15	4	0.27 (0.11, 0.52)	3.33
Balkan et al (2012)	11	3	0.27 (0.10, 0.57)	2.82
Bonetto et al (2011)	17	1	0.06 (0.01, 0.27)	5.14
Choi et al (2017)	95	20	0.21 (0.14, 0.30)	5.61
Cohen–Gadol et al (2006)	13	1	0.08 (0.01, 0.33)	4.58
Davies et al (2020)	86	31	0.36 (0.27, 0.47)	5.31
He et al (2017)	165	31	0.19 (0.14, 0.25)	5.91
Hyun et al (2008)	30	4	0.13 (0.05, 0.30)	4.98
Kim et al (2016)	140	10	0.07 (0.04, 0.13)	6.09
Kim et al (2017)	128	24	0.19 (0.13, 0.26)	5.81
Levi et al (2019)	101	38	0.38 (0.29, 0.47)	5.42
Martin et al (2019)	94	41	0.44 (0.34, 0.54)	5.33
Miyazawa et al (2003)	10	1	0.10 (0.02, 0.40)	3.89
Ohtonary et al (2018)	19	3	0.16 (0.06, 0.38)	4.25
Su et al (2009)	11	1	0.09 (0.02, 0.38)	4.15
Tanaka et al (2016)	40	14	0.35 (0.22, 0.50)	4.53
Tomoda et al (2008)	27	3	0.11 (0.04, 0.28)	5.03
Wiesemann et al (2006)	10	1	0.10 (0.02, 0.40)	3.89
Wu et al (2017)	150	62	0.41 (0.34, 0.49)	5.66
Yoshida et al (2014)	12	1	0.08 (0.01, 0.35)	4.38
Overall (I^2 = 84.75%, p = 0.00)			0.20 (0.14, 0.26)	100.00

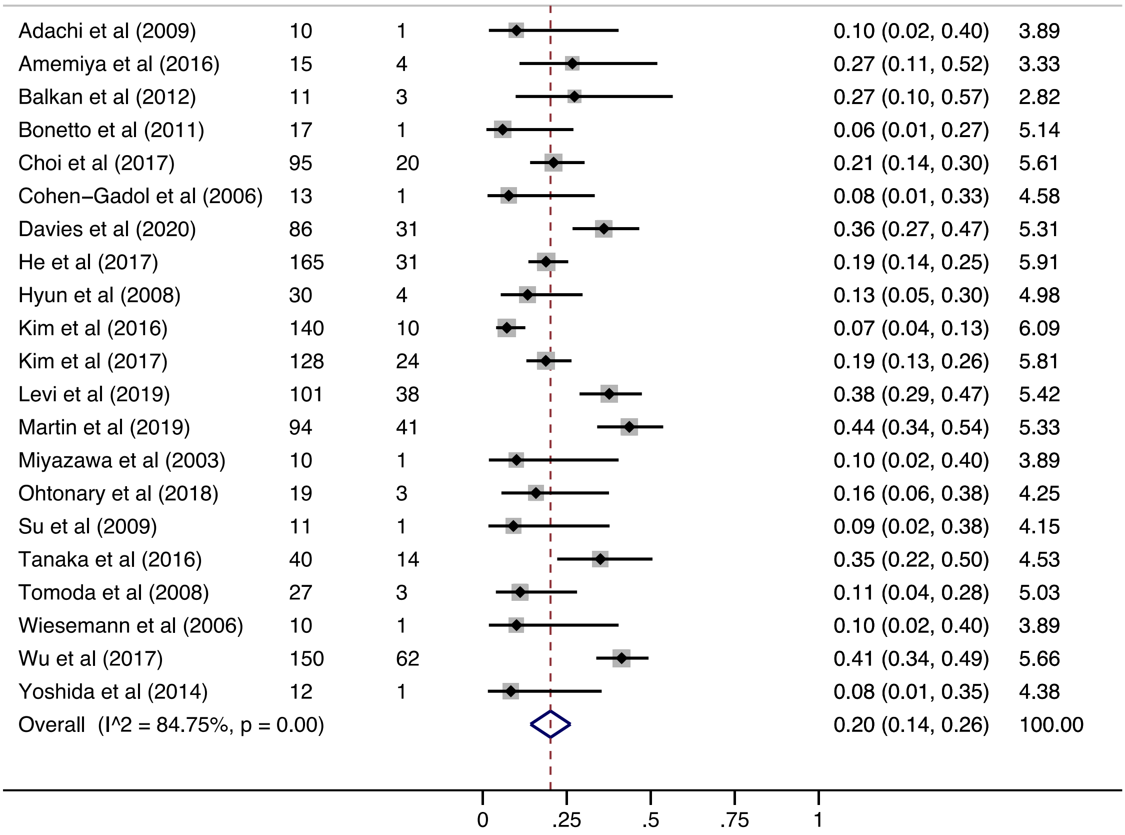

© 2021 D'Antona et al. *JAMA Neurology.*

eFigure 8d. Meta-analysis of proportions of patients with dizziness

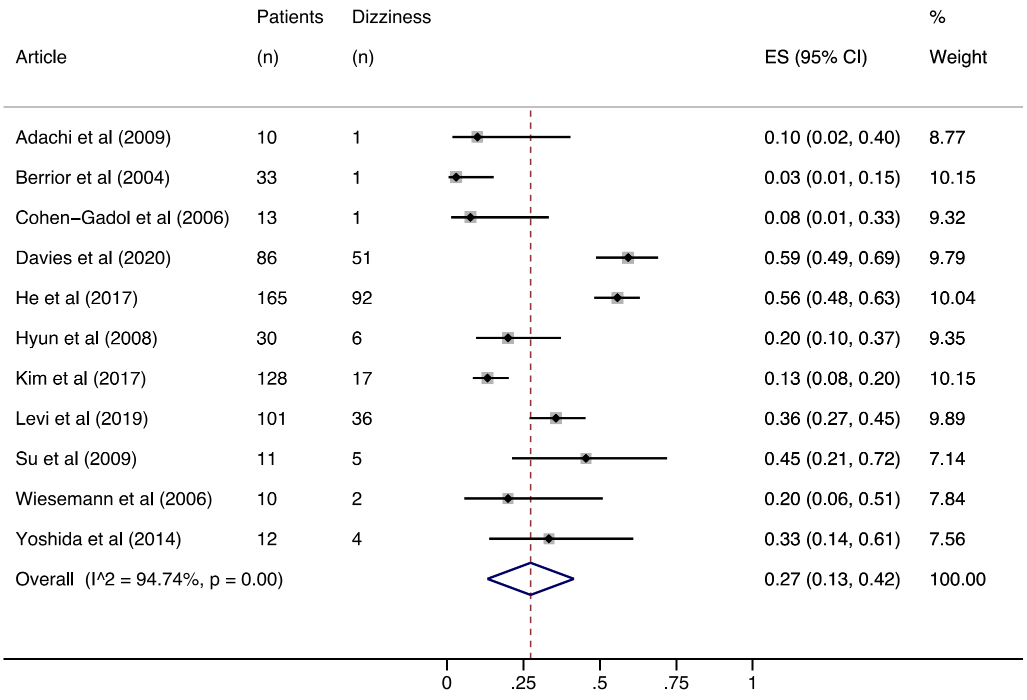

eFigure 8e. Meta-analysis of proportions of patients with hearing disturbances

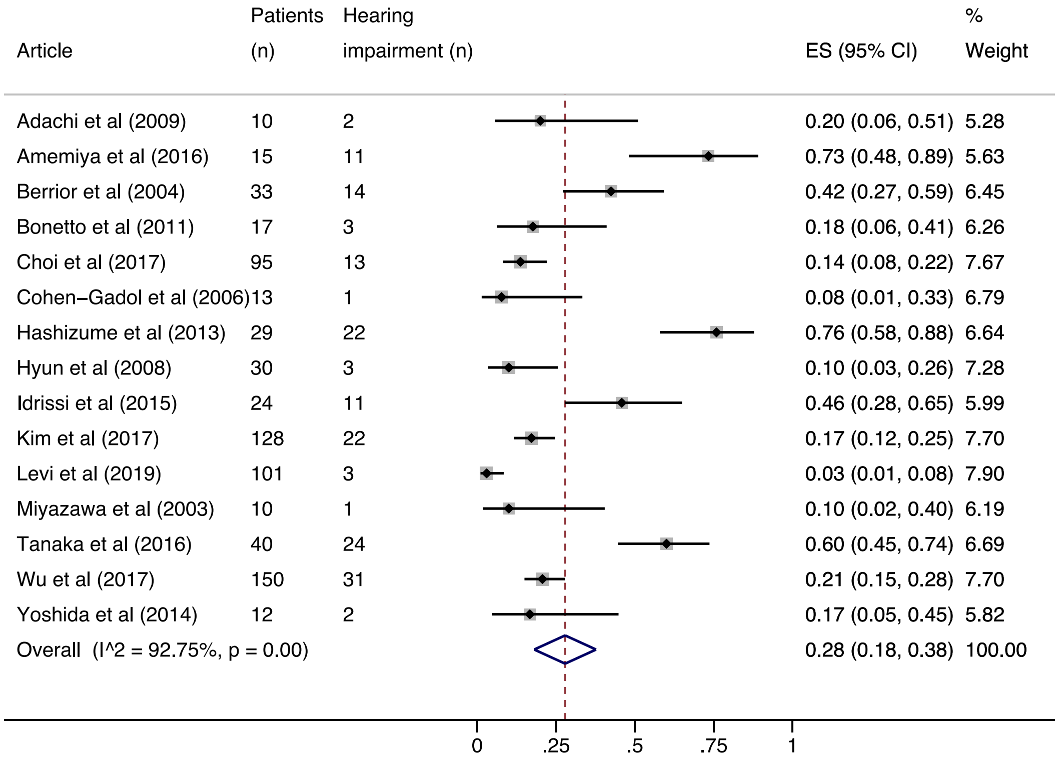

© 2021 D'Antona et al. *JAMA Neurology.*

eFigure 8f. Meta-analysis of proportions of patients with photophobia

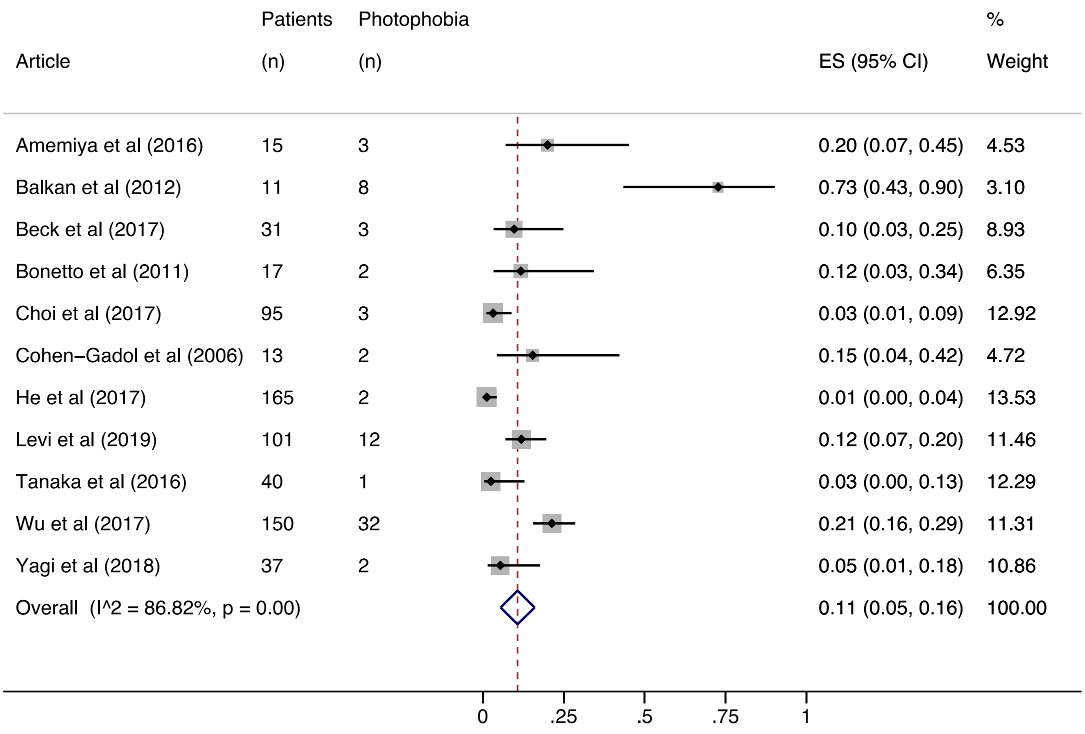

eFigure 8g. Meta-analysis of proportions of patients with cognitive symptoms

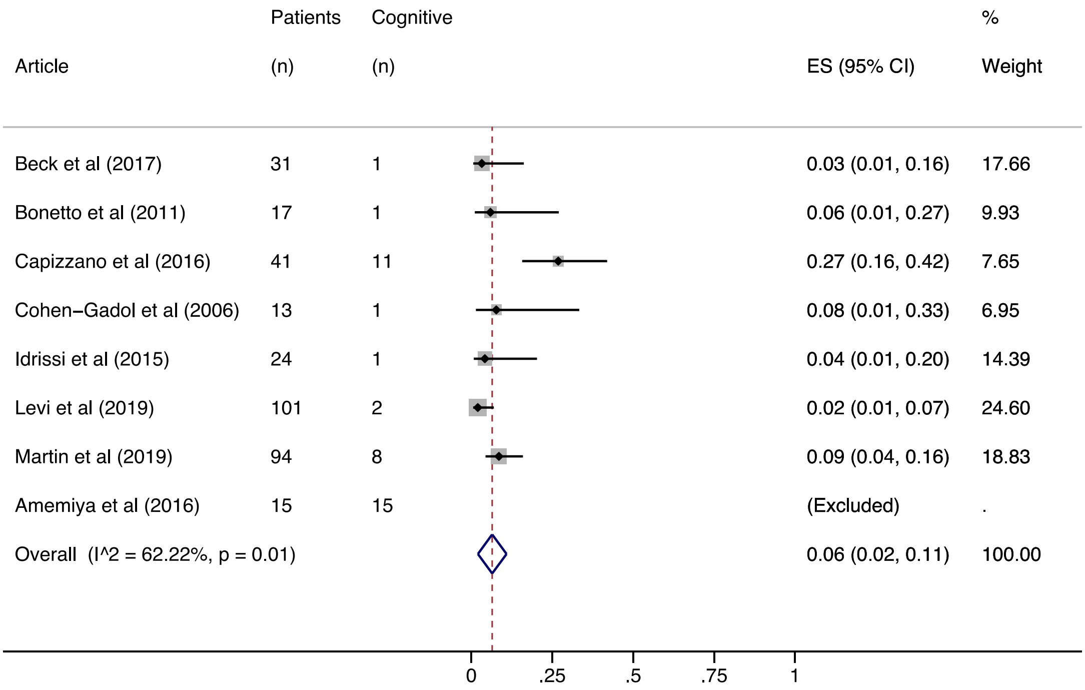

© 2021 D’Antona et al. *JAMA Neurology.*

eFigure 8h. Meta-analysis of proportions of patients with diplopia

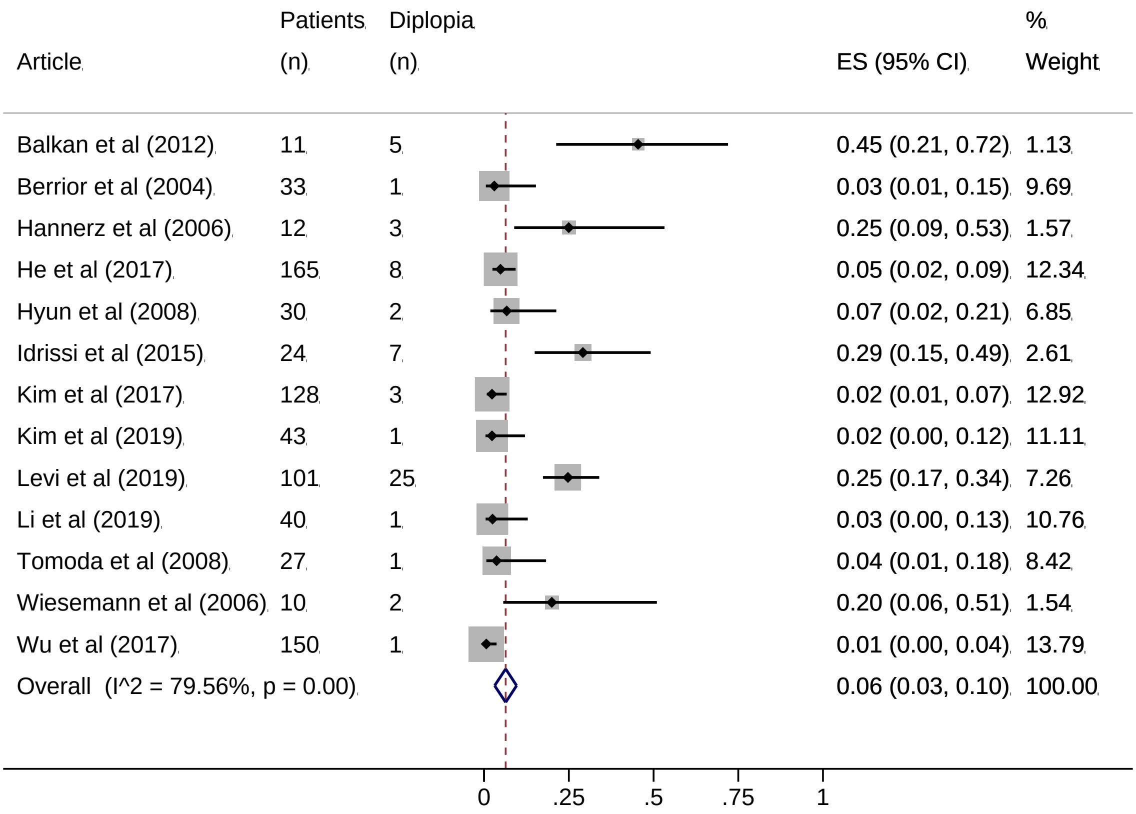

eFigure 8i. Meta-analysis of proportions of patients with other ear-related symptoms

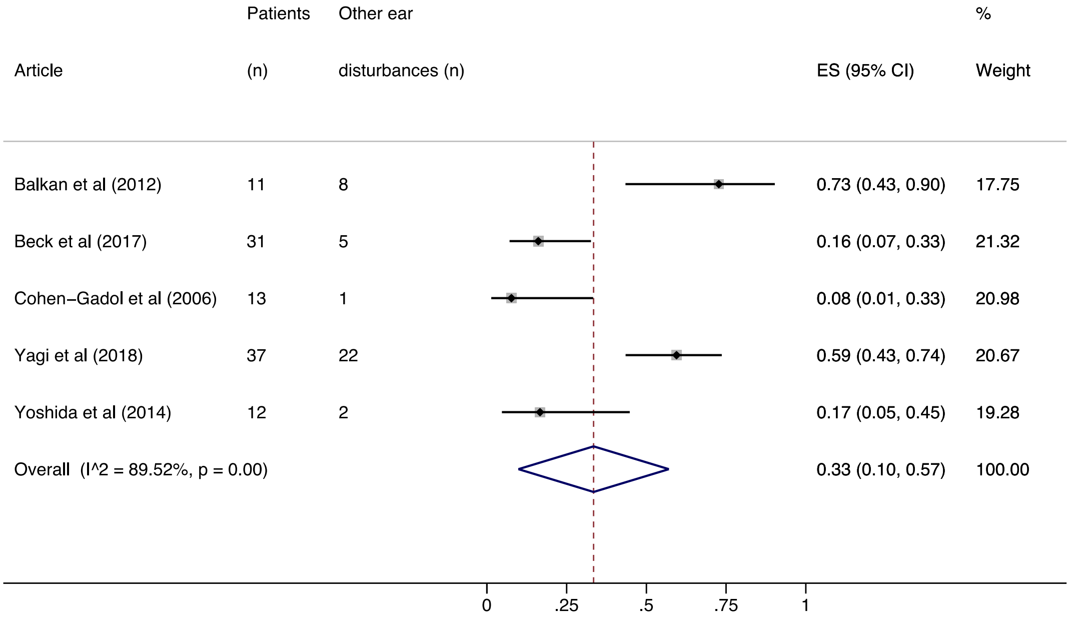

© 2021 D'Antona et al. *JAMA Neurology.*

eFigure 8l. Meta-analysis of proportions of patients with vertigo

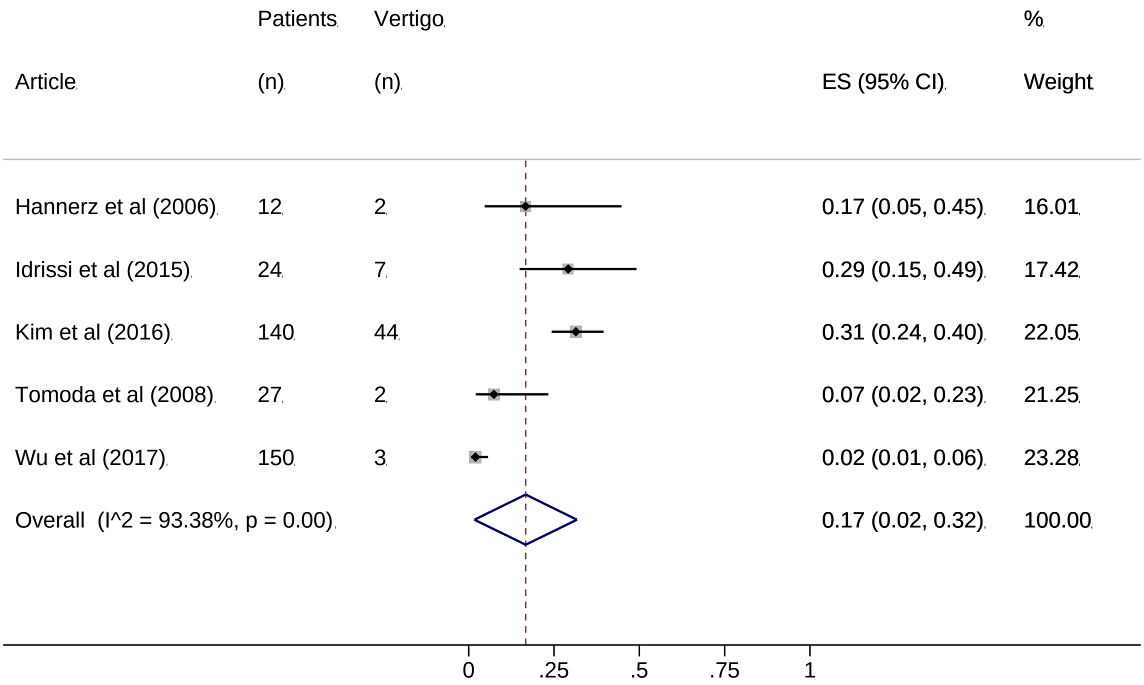

eFigure 8m. Meta-analysis of proportions of patients with movement disorders

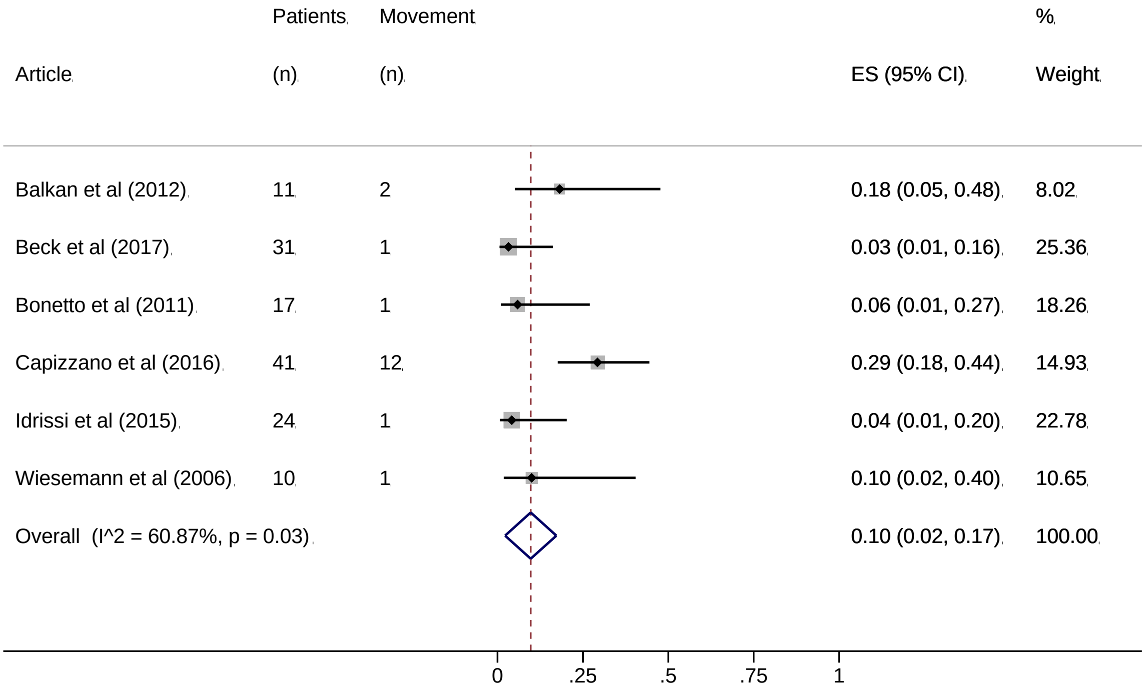

© 2021 D’Antona et al. *JAMA Neurology.*

eFigure 8n. Meta-analysis of proportions of patients with back pain

Article	Patients (n)	Back Pain (n)	ES (95% CI)	% Weight
Balkan et al (2012)	11	7	0.64 (0.35, 0.85)	4.23
Beck et al (2017)	31	2	0.06 (0.02, 0.21)	13.17
Berrior et al (2004)	33	3	0.09 (0.03, 0.24)	12.41
Bonetto et al (2011)	17	2	0.12 (0.03, 0.34)	9.02
Cohen–Gadol et al (2006)	13	1	0.08 (0.01, 0.33)	9.48
He et al (2017)	165	16	0.10 (0.06, 0.15)	15.62
Idrissi et al (2015)	24	1	0.04 (0.01, 0.20)	13.60
Ohtonary et al (2018)	19	2	0.11 (0.03, 0.31)	9.88
Park et al (2009)	12	3	0.25 (0.09, 0.53)	5.24
Tomoda et al (2008)	27	12	0.44 (0.28, 0.63)	7.33
Overall (I^2 = 70.93%, p = 0.00)			0.14 (0.07, 0.21)	100.00

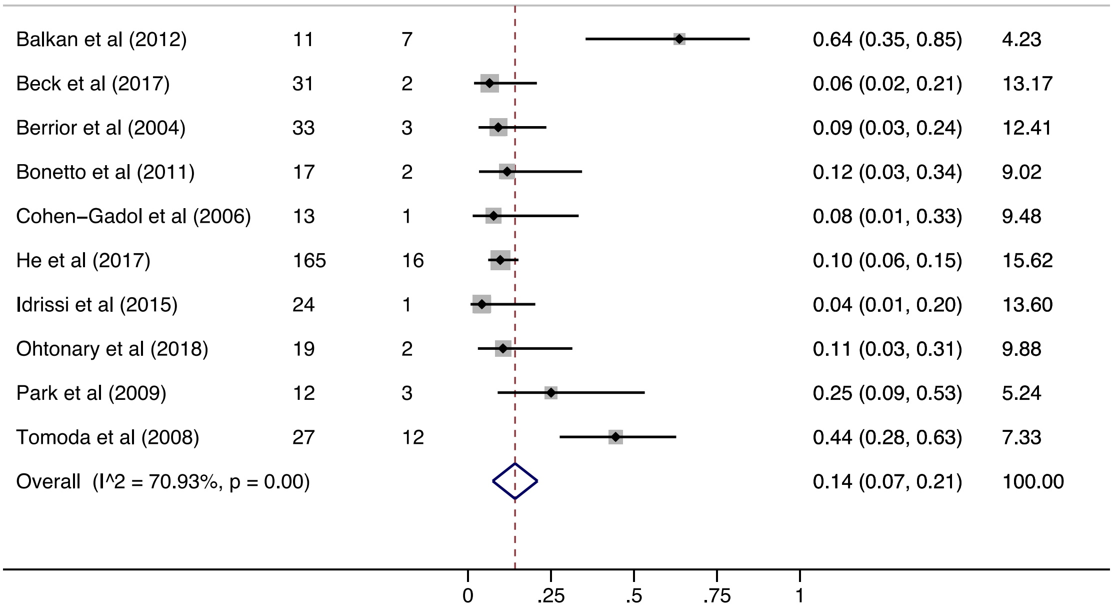

eFigure 8o. Meta-analysis of proportions of patients with other visual symptoms

Article	Patients (n)	Other visual disturbances (n)	ES (95% CI)	% Weight
Balkan et al (2012)	11	6	0.55 (0.28, 0.79)	3.96
Beck et al (2017)	31	3	0.10 (0.03, 0.25)	10.13
Capizzano et al (2016)	41	6	0.15 (0.07, 0.28)	9.95
Cohen–Gadol et al (2006)	13	2	0.15 (0.04, 0.42)	6.46
Davies et al (2020)	86	18	0.21 (0.14, 0.31)	10.90
Hashizume et al (2013)	29	9	0.31 (0.17, 0.49)	7.45
Kim et al (2017)	128	1	0.01 (0.00, 0.04)	12.96
Martin et al (2019)	94	15	0.16 (0.10, 0.25)	11.39
Park et al (2009)	12	1	0.08 (0.01, 0.35)	7.91
Su et al (2009)	11	1	0.09 (0.02, 0.38)	7.39
Tomoda et al (2008)	27	1	0.04 (0.01, 0.18)	11.49
Overall (I^2 = 85.19%, p = 0.00)			0.14 (0.07, 0.21)	100.00

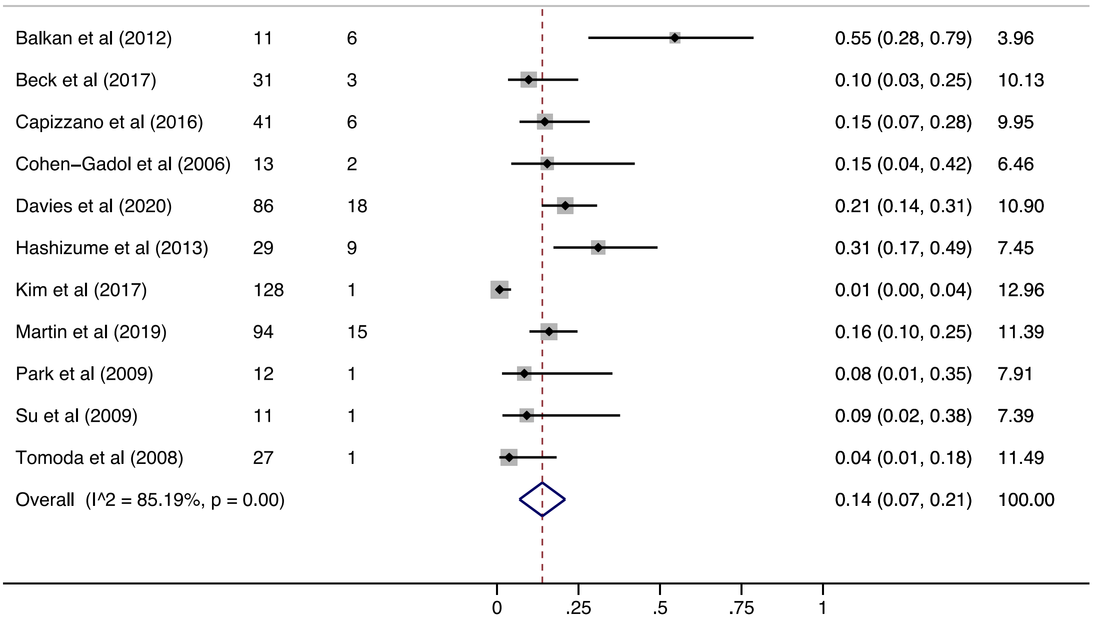

© 2021 D'Antona et al. *JAMA Neurology.*

eFigure 8p. Meta-analysis of proportions of patients with reduced consciousness

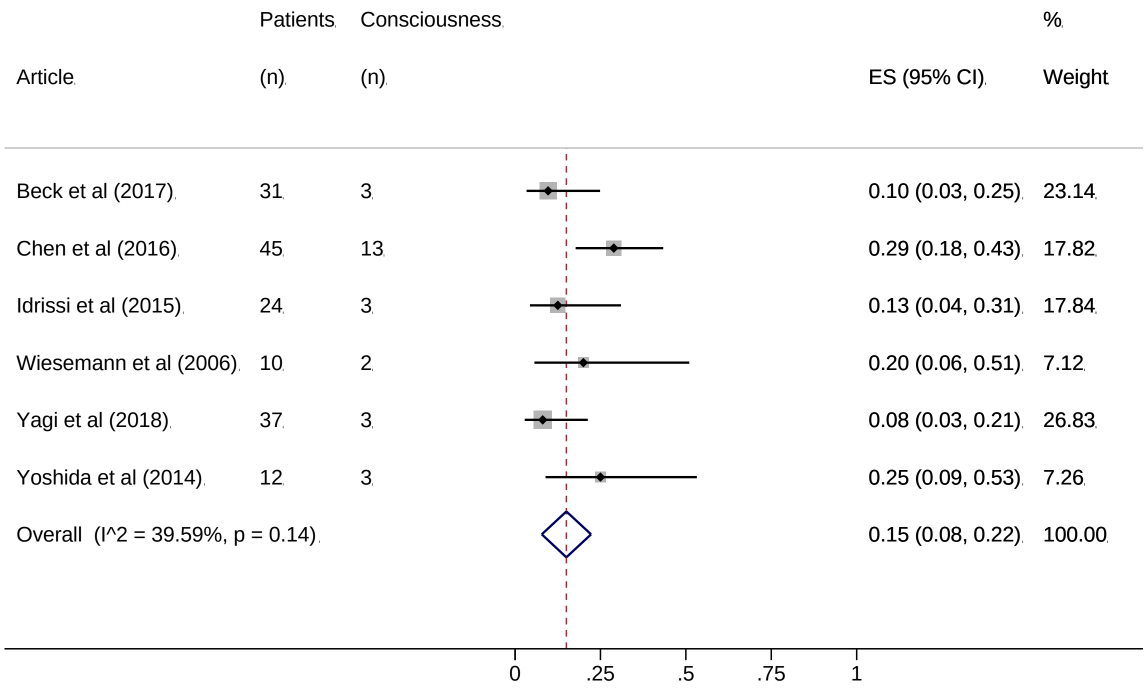

© 2021 D'Antona et al. *JAMA Neurology.*

eFigure 9a. Meta-analysis of proportions of diffuse pachymeningeal enhancement (DPE) in brain MRI

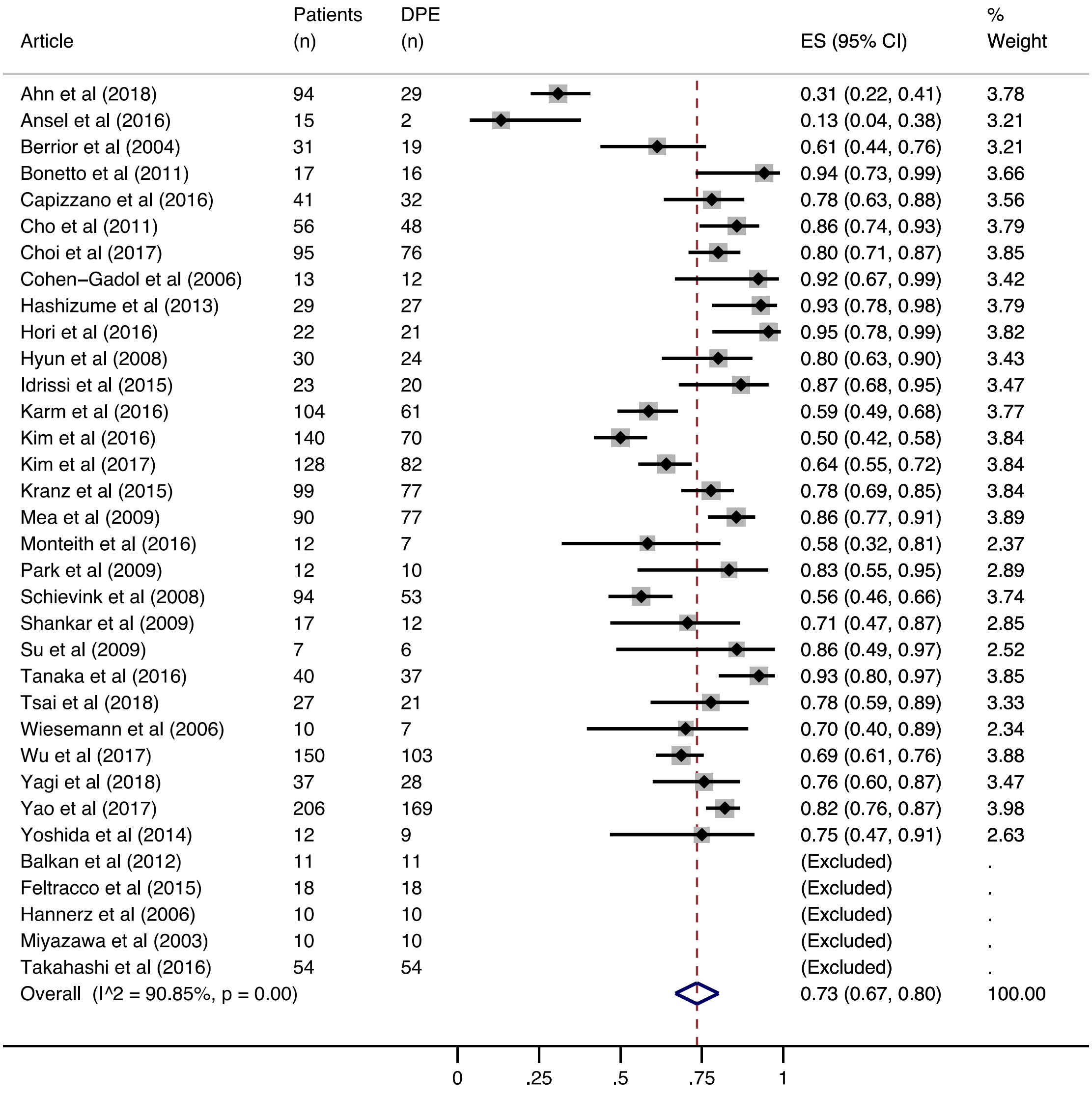

Article	Patients (n)	DPE (n)	ES (95% CI)	% Weight
Ahn et al (2018)	94	29	0.31 (0.22, 0.41)	3.78
Ansel et al (2016)	15	2	0.13 (0.04, 0.38)	3.21
Berrior et al (2004)	31	19	0.61 (0.44, 0.76)	3.21
Bonetto et al (2011)	17	16	0.94 (0.73, 0.99)	3.66
Capizzano et al (2016)	41	32	0.78 (0.63, 0.88)	3.56
Cho et al (2011)	56	48	0.86 (0.74, 0.93)	3.79
Choi et al (2017)	95	76	0.80 (0.71, 0.87)	3.85
Cohen−Gadol et al (2006)	13	12	0.92 (0.67, 0.99)	3.42
Hashizume et al (2013)	29	27	0.93 (0.78, 0.98)	3.79
Hori et al (2016)	22	21	0.95 (0.78, 0.99)	3.82
Hyun et al (2008)	30	24	0.80 (0.63, 0.90)	3.43
Idrissi et al (2015)	23	20	0.87 (0.68, 0.95)	3.47
Karm et al (2016)	104	61	0.59 (0.49, 0.68)	3.77
Kim et al (2016)	140	70	0.50 (0.42, 0.58)	3.84
Kim et al (2017)	128	82	0.64 (0.55, 0.72)	3.84
Kranz et al (2015)	99	77	0.78 (0.69, 0.85)	3.84
Mea et al (2009)	90	77	0.86 (0.77, 0.91)	3.89
Monteith et al (2016)	12	7	0.58 (0.32, 0.81)	2.37
Park et al (2009)	12	10	0.83 (0.55, 0.95)	2.89
Schievink et al (2008)	94	53	0.56 (0.46, 0.66)	3.74
Shankar et al (2009)	17	12	0.71 (0.47, 0.87)	2.85
Su et al (2009)	7	6	0.86 (0.49, 0.97)	2.52
Tanaka et al (2016)	40	37	0.93 (0.80, 0.97)	3.85
Tsai et al (2018)	27	21	0.78 (0.59, 0.89)	3.33
Wiesemann et al (2006)	10	7	0.70 (0.40, 0.89)	2.34
Wu et al (2017)	150	103	0.69 (0.61, 0.76)	3.88
Yagi et al (2018)	37	28	0.76 (0.60, 0.87)	3.47
Yao et al (2017)	206	169	0.82 (0.76, 0.87)	3.98
Yoshida et al (2014)	12	9	0.75 (0.47, 0.91)	2.63
Balkan et al (2012)	11	11	(Excluded)	.
Feltracco et al (2015)	18	18	(Excluded)	.
Hannerz et al (2006)	10	10	(Excluded)	.
Miyazawa et al (2003)	10	10	(Excluded)	.
Takahashi et al (2016)	54	54	(Excluded)	.
Overall (I^2 = 90.85%, p = 0.00)			0.73 (0.67, 0.80)	100.00

© 2021 D'Antona et al. *JAMA Neurology.*

eFigure 9b. Meta-analysis of proportions of subdural collections (SDC) in brain MRI

Article	Patients (n)	SDC (n)	ES (95% CI)	% Weight
Ahn et al (2018)	94	36	0.38 (0.29, 0.48)	3.77
Ansel et al (2016)	15	3	0.20 (0.07, 0.45)	2.97
Balkan et al (2012)	11	2	0.18 (0.05, 0.48)	2.76
Berrior et al (2004)	31	11	0.35 (0.21, 0.53)	3.25
Bonetto et al (2011)	17	7	0.41 (0.22, 0.64)	2.72
Capizzano et al (2016)	41	16	0.39 (0.26, 0.54)	3.40
Cho et al (2011)	56	13	0.23 (0.14, 0.36)	3.69
Choi et al (2017)	95	48	0.51 (0.41, 0.60)	3.75
Cohen–Gadol et al (2006)	13	2	0.15 (0.04, 0.42)	3.02
Feltracco et al (2015)	18	4	0.22 (0.09, 0.45)	3.05
Hannerz et al (2006)	10	3	0.30 (0.11, 0.60)	2.34
Hashizume et al (2013)	29	9	0.31 (0.17, 0.49)	3.25
Hori et al (2016)	22	14	0.64 (0.43, 0.80)	2.98
Hyun et al (2008)	30	8	0.27 (0.14, 0.44)	3.33
Idrissi et al (2015)	23	17	0.74 (0.54, 0.87)	3.16
Karm et al (2016)	104	11	0.11 (0.06, 0.18)	3.98
Kim et al (2016)	140	34	0.24 (0.18, 0.32)	3.92
Kim et al (2017)	128	20	0.16 (0.10, 0.23)	3.96
Mea et al (2009)	90	65	0.72 (0.62, 0.80)	3.80
Miyazawa et al (2003)	10	1	0.10 (0.02, 0.40)	3.10
Park et al (2009)	12	2	0.17 (0.05, 0.45)	2.90
Schievink et al (2008)	94	34	0.36 (0.27, 0.46)	3.78
Shankar et al (2009)	17	11	0.65 (0.41, 0.83)	2.77
Su et al (2009)	7	3	0.43 (0.16, 0.75)	1.82
Tanaka et al (2016)	40	17	0.43 (0.29, 0.58)	3.37
Tsai et al (2018)	27	7	0.26 (0.13, 0.45)	3.27
Wiesemann et al (2006)	10	4	0.40 (0.17, 0.69)	2.20
Wu et al (2017)	150	54	0.36 (0.29, 0.44)	3.89
Yagi et al (2018)	37	19	0.51 (0.36, 0.67)	3.31
Yao et al (2017)	206	38	0.18 (0.14, 0.24)	4.00
Yoshida et al (2014)	12	8	0.67 (0.39, 0.86)	2.47
Takahashi et al (2016)	54	54	(Excluded)	.
Overall (I^2 = 88.45%, p = 0.00)			0.35 (0.28, 0.42)	100.00

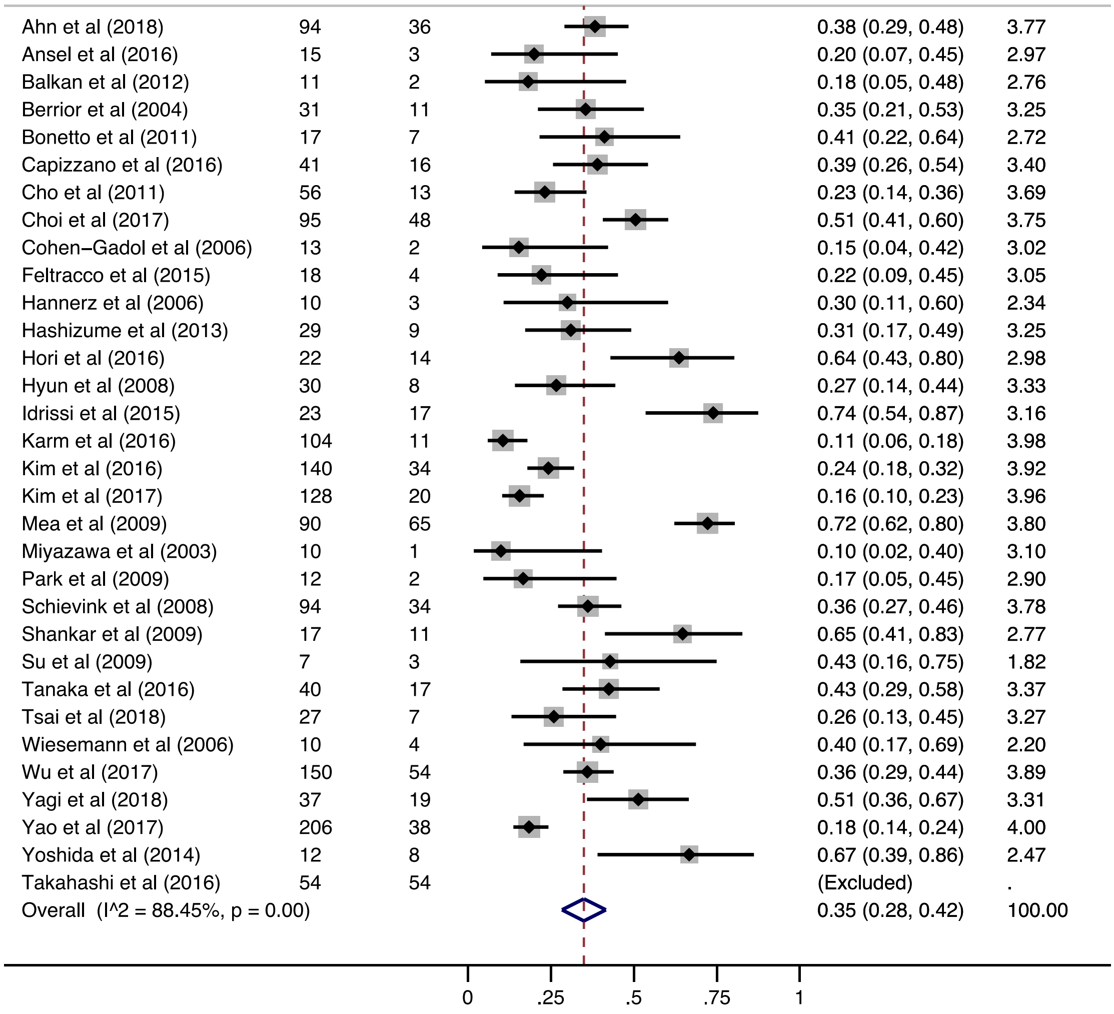

© 2021 D’Antona et al. *JAMA Neurology.*

eFigure 9c. Meta-analysis of proportions of brain sagging brain MRI

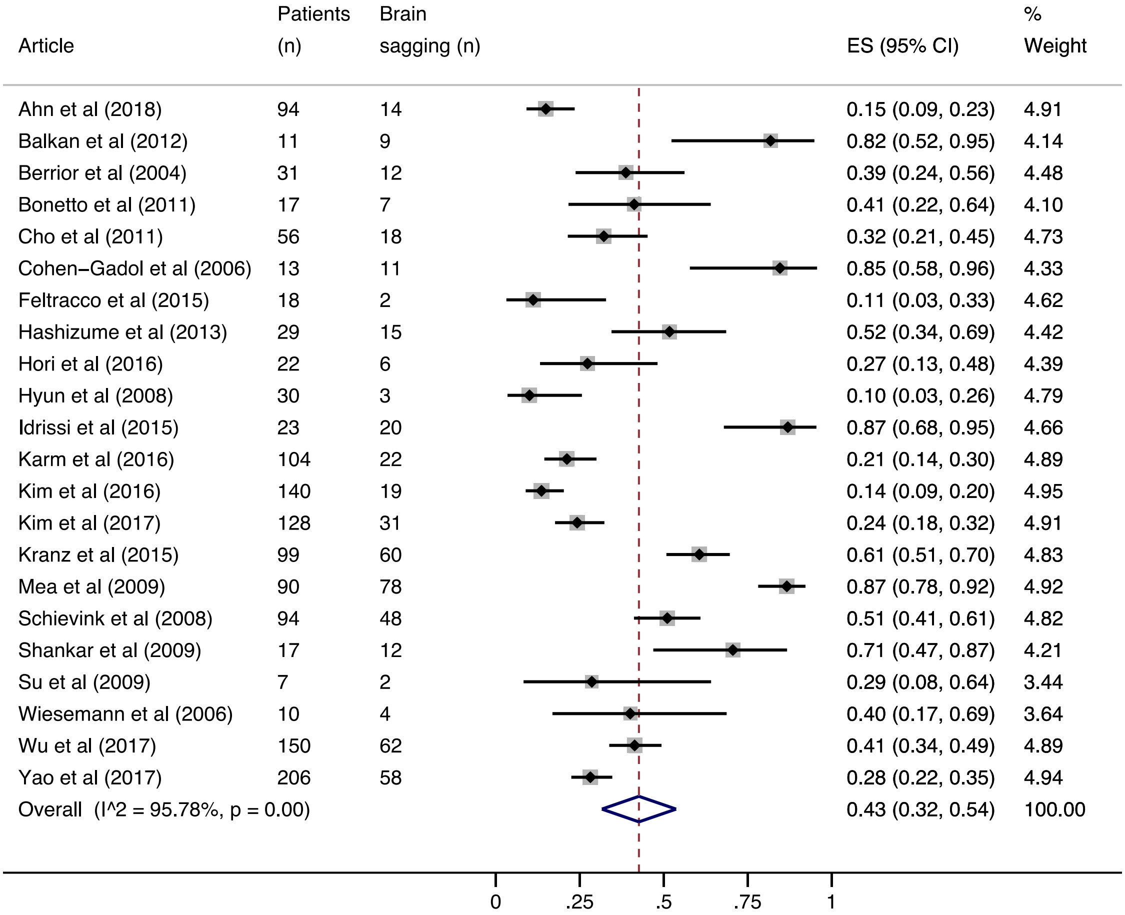

© 2021 D'Antona et al. *JAMA Neurology.*

eFigure 9d. Meta-analysis of proportions of enlarged pituitary gland (EPG) in brain MRI

Article	Patients (n)	EPG (n)	ES (95% CI)	% Weight
Balkan et al (2012)	11	6	0.55 (0.28, 0.79)	8.13
Bonetto et al (2011)	17	1	0.06 (0.01, 0.27)	9.21
Choi et al (2017)	95	15	0.16 (0.10, 0.24)	9.33
Karm et al (2016)	104	14	0.13 (0.08, 0.21)	9.35
Kim et al (2016)	140	2	0.01 (0.00, 0.05)	9.42
Kim et al (2017)	128	22	0.17 (0.12, 0.25)	9.35
Mea et al (2009)	90	38	0.42 (0.33, 0.53)	9.25
Shankar et al (2009)	17	7	0.41 (0.22, 0.64)	8.57
Tanaka et al (2016)	40	28	0.70 (0.55, 0.82)	9.09
Tsai et al (2018)	27	18	0.67 (0.48, 0.81)	8.91
Wu et al (2017)	150	134	0.89 (0.83, 0.93)	9.38
Overall (I^2 = 99.16%, p = 0.00)			0.38 (0.15, 0.60)	100.00

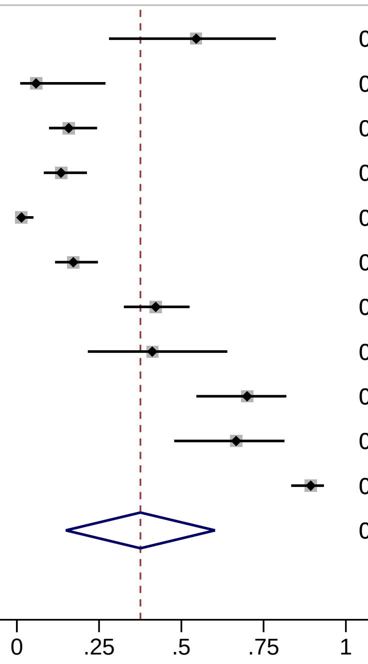

eFigure 9e. Meta-analysis of proportions of venous engorgement in brain MRI

Article	Patients (n)	VE (n)	ES (95% CI)	% Weight
Balkan et al (2012)	11	5	0.45 (0.21, 0.72)	11.91
Choi et al (2017)	95	70	0.74 (0.64, 0.81)	17.59
Karm et al (2016)	104	40	0.38 (0.30, 0.48)	17.50
Kim et al (2017)	128	56	0.44 (0.35, 0.52)	17.64
Mea et al (2009)	90	48	0.53 (0.43, 0.63)	17.30
Wu et al (2017)	150	124	0.83 (0.76, 0.88)	18.05
Overall (I^2 = 94.82%, p = 0.00)			0.57 (0.40, 0.74)	100.00

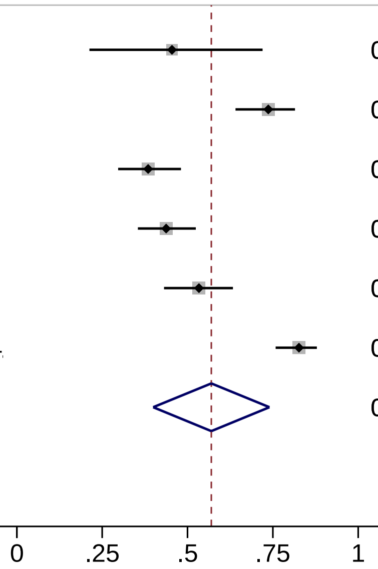

© 2021 D'Antona et al. *JAMA Neurology.*

eFigure 9f. Meta-analysis of proportions of normal brain MRI

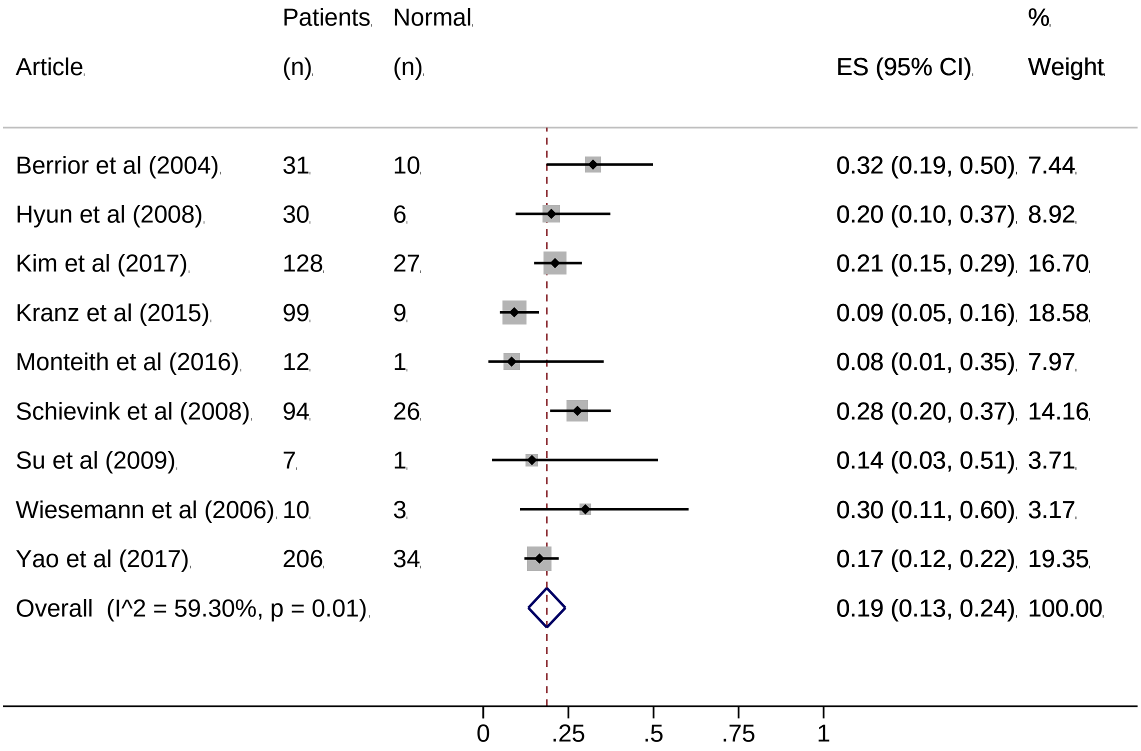

© 2021 D'Antona et al. *JAMA Neurology.*

eFigure 10. Meta-analysis of proportions of positive findings in spinal MRI

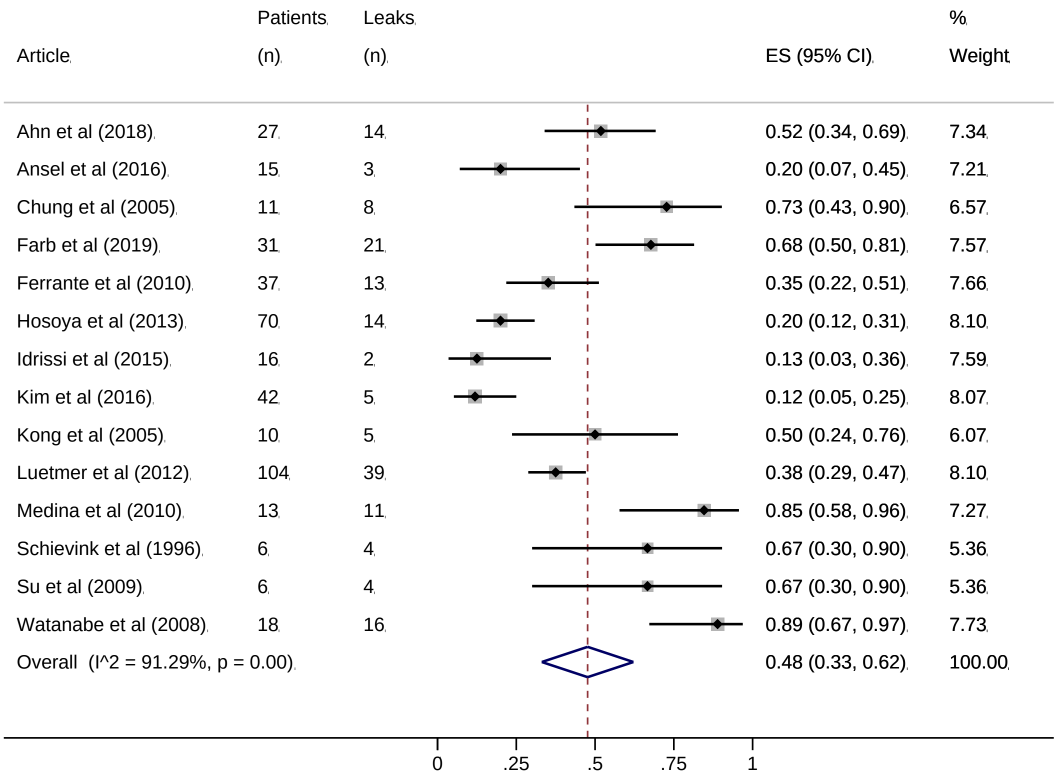

eFigure 11. Meta-analysis of proportions of positive findings in CT myelography (CTM)

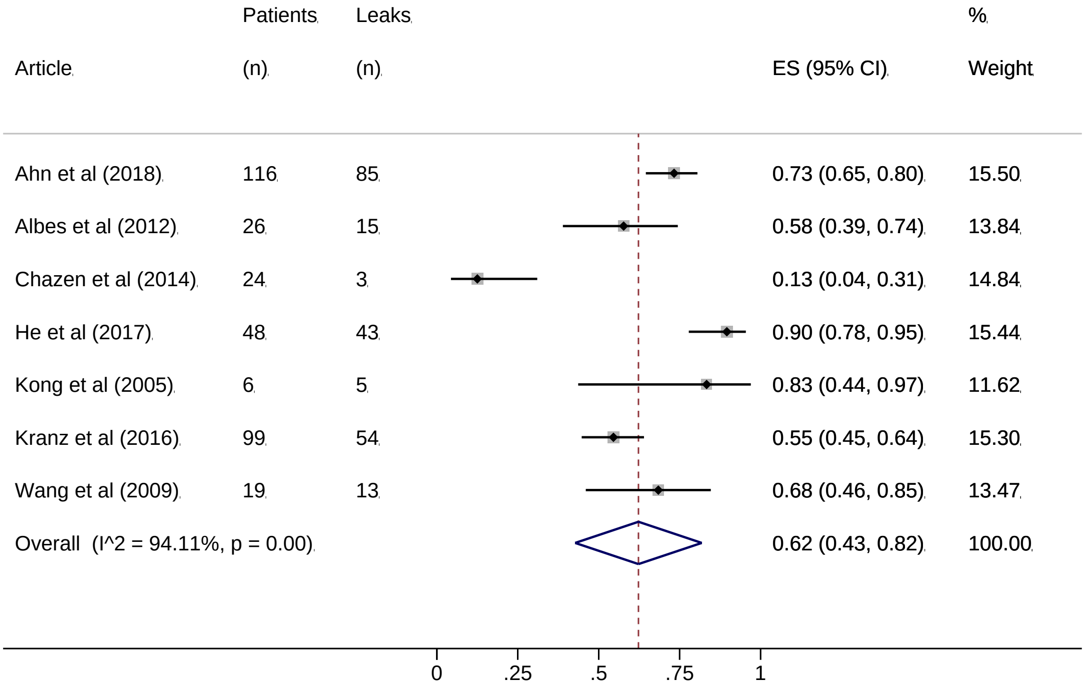

© 2021 D’Antona et al. *JAMA Neurology.*

eFigure 12. Meta-analysis of proportions of positive findings in MR myelography

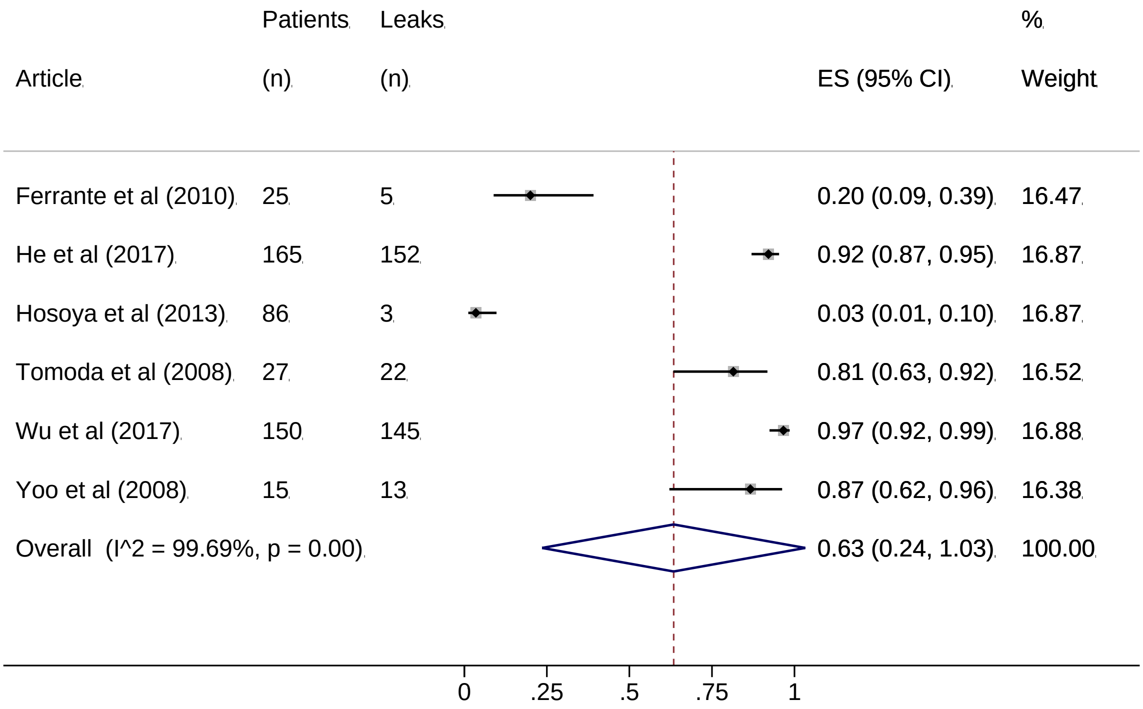

eFigure 13. Meta-analysis of proportions of positive findings in MR myelography with intrathecal gadolinium

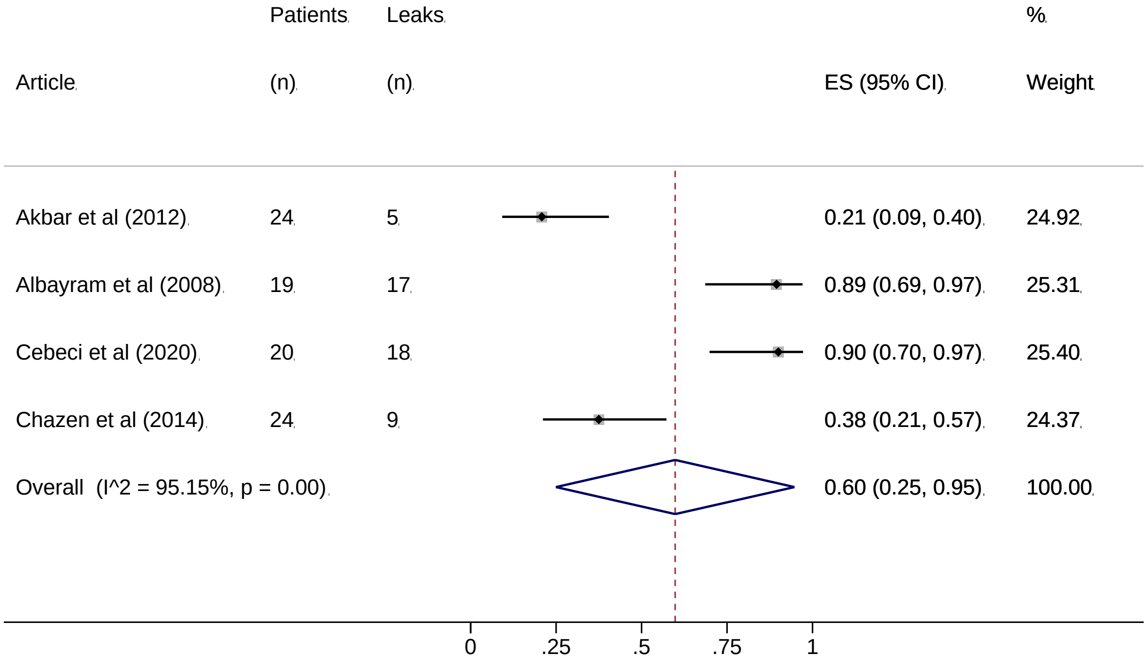

© 2021 D'Antona et al. *JAMA Neurology.*

eFigure 14. Meta-analysis of proportions of positive findings in radionuclide cisternography

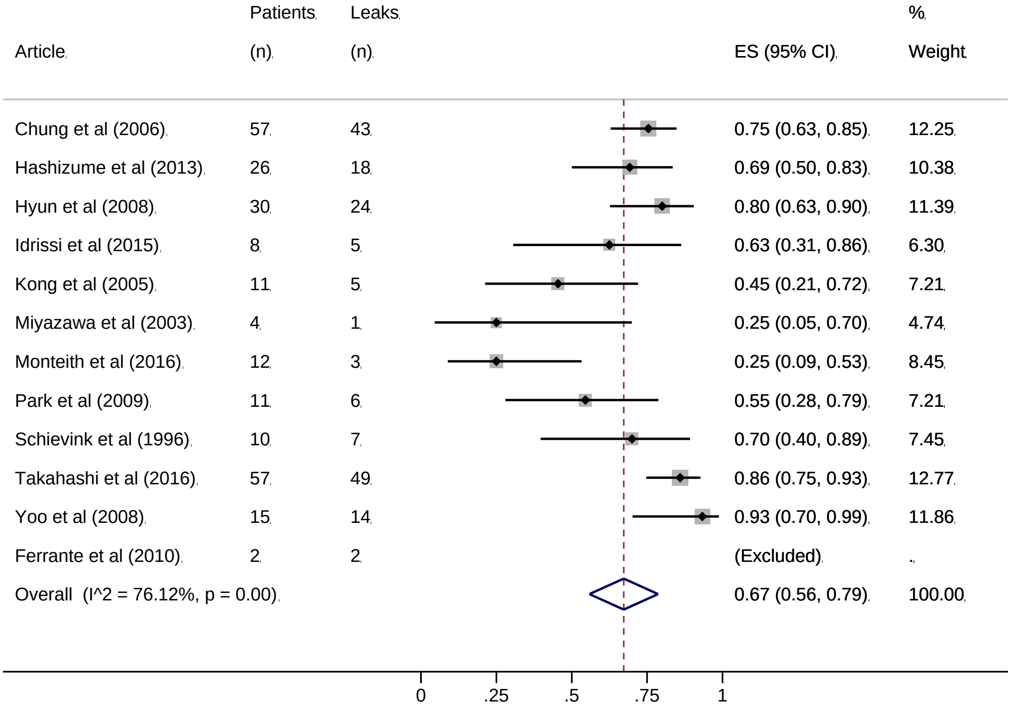

eFigure 15. Meta-analysis of proportions of positive findings in digital subtraction myelography

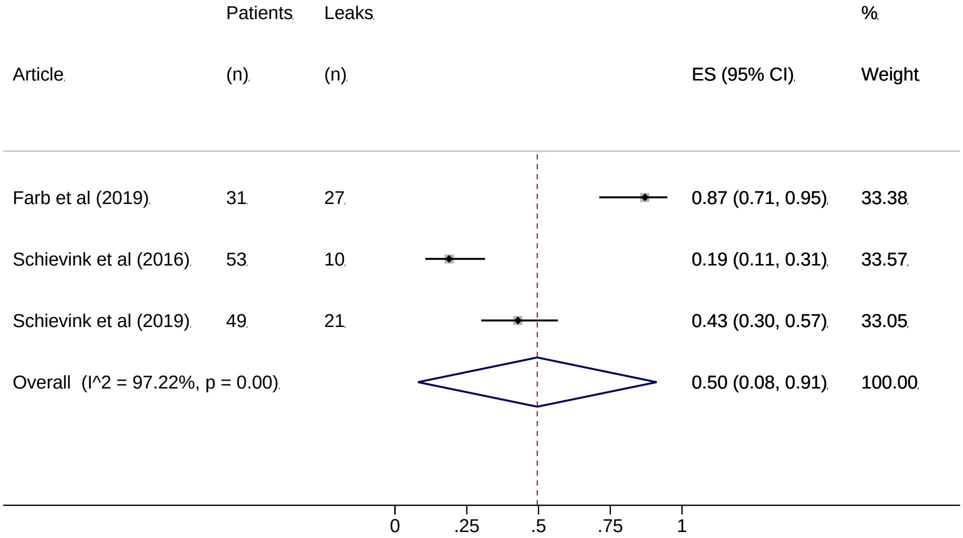

The two studies by Schievink *et al.* have clearly been reported to come from different groups of patients, therefore, they were both eligible for the meta-analysis.

© 2021 D’Antona et al. *JAMA Neurology.*

eFigure 16. Meta-analysis of proportions of positive findings in digital subtraction myelography performed in lateral decubitus position

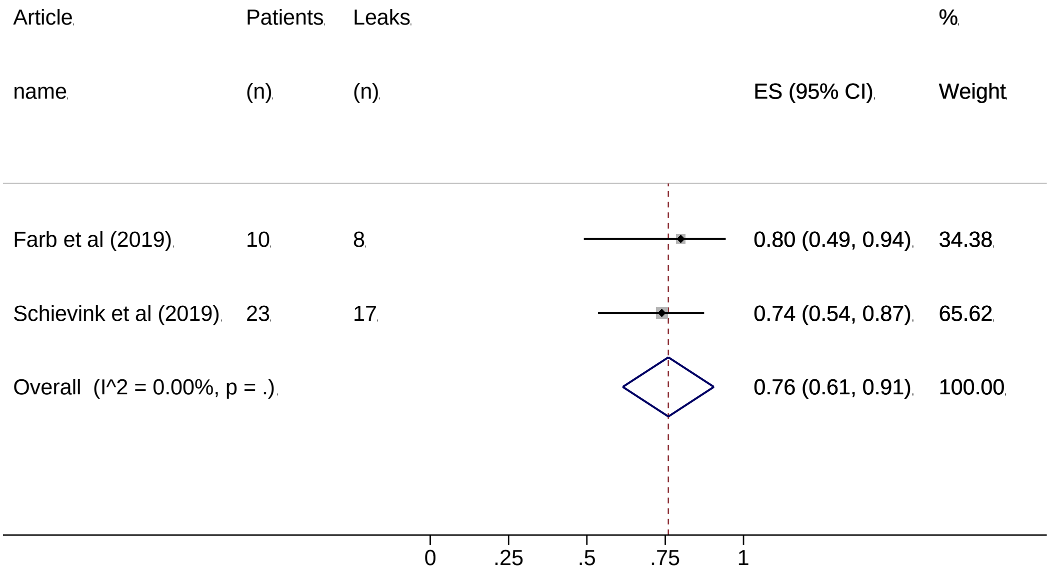

© 2021 D'Antona et al. *JAMA Neurology.*

eFigure 17a. Meta-analysis of proportions of leak location (cervical)

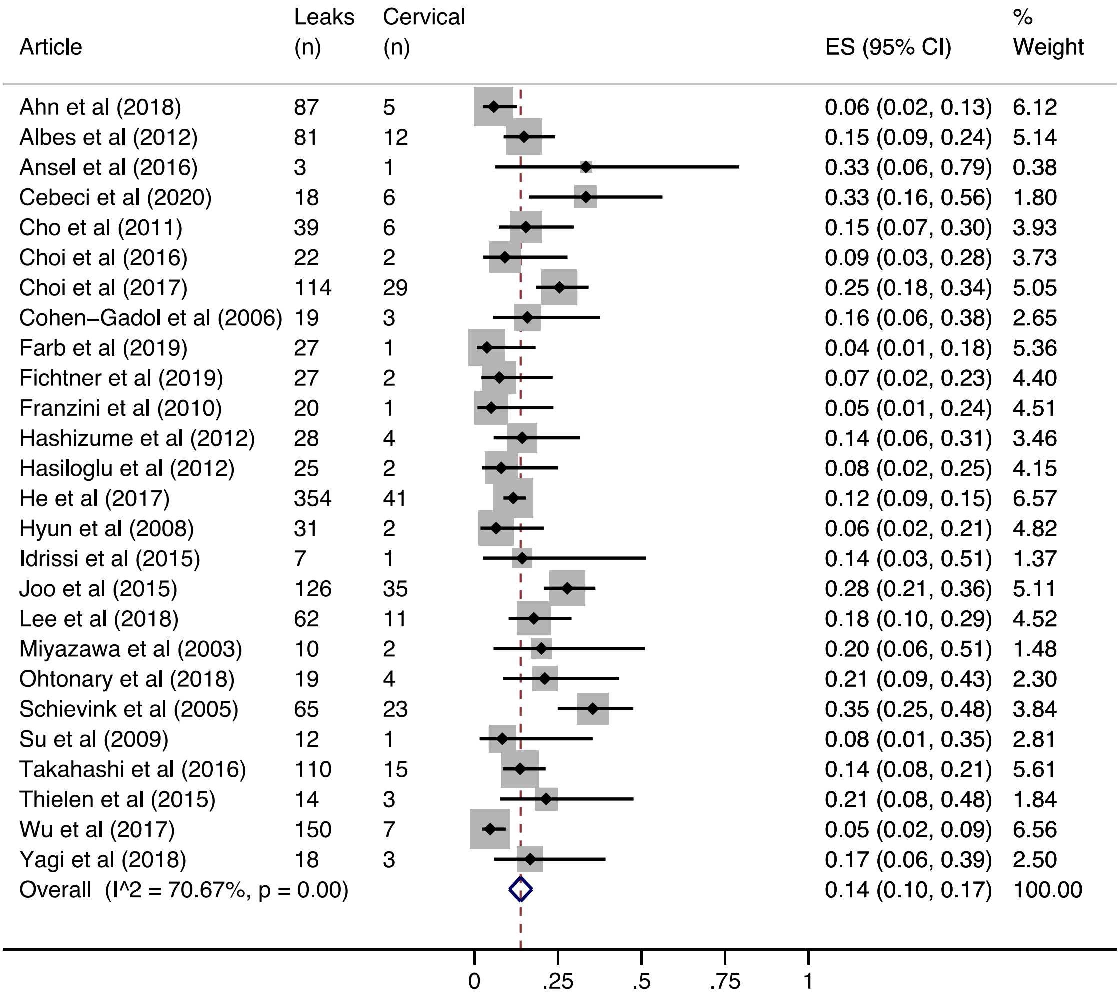

© 2021 D’Antona et al. *JAMA Neurology.*

eFigure 17b. Meta-analysis of proportions of leak location (cervico-thoracic)

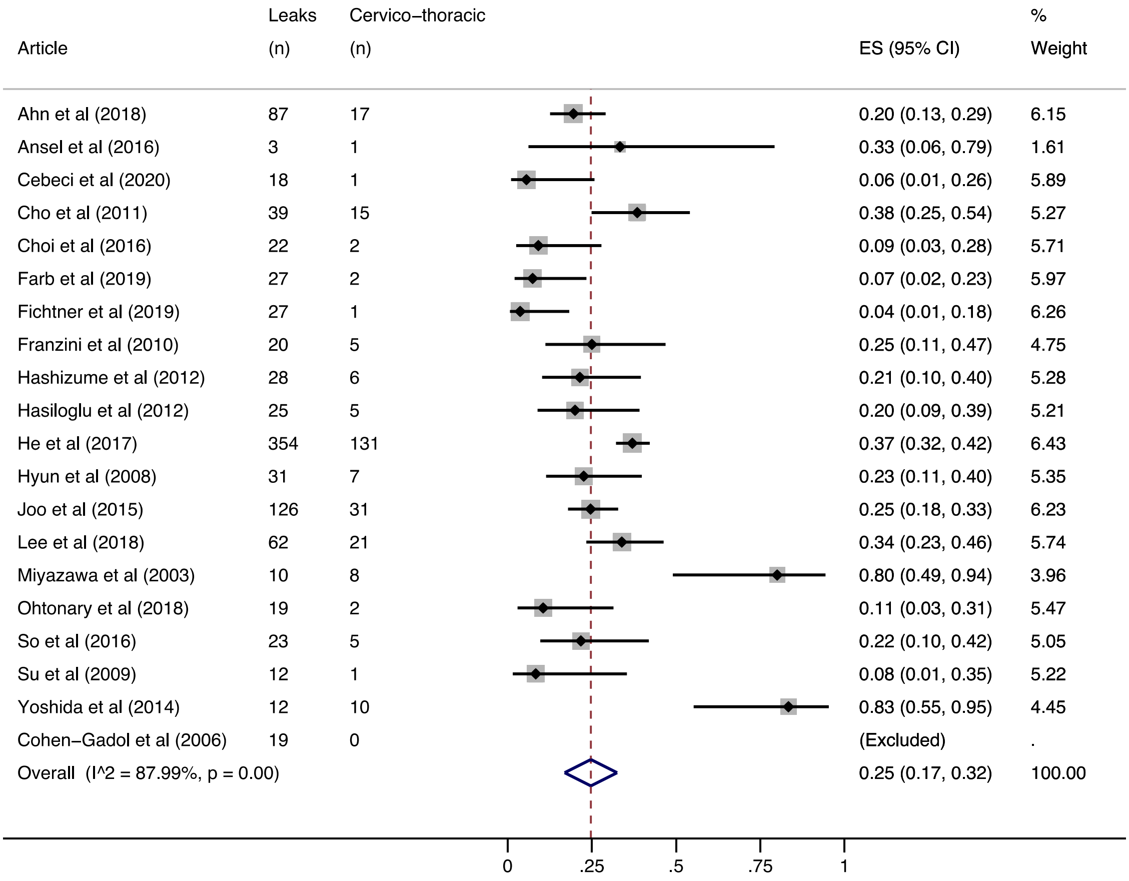

© 2021 D’Antona et al. *JAMA Neurology.*

eFigure 17c. Meta-analysis of proportions of leak location (thoracic)

Article	Leaks (n)	Thoracic (n)	ES (95% CI)	% Weight
Ahn et al (2018)	87	4	0.05 (0.02, 0.11)	4.15
Albes et al (2012)	81	25	0.31 (0.22, 0.42)	4.05
Cebeci et al (2020)	18	5	0.28 (0.12, 0.51)	3.68
Cho et al (2011)	39	8	0.21 (0.11, 0.36)	3.97
Choi et al (2016)	22	8	0.36 (0.20, 0.57)	3.70
Choi et al (2017)	114	49	0.43 (0.34, 0.52)	4.07
Cohen–Gadol et al (2006)	19	9	0.47 (0.27, 0.68)	3.60
Farb et al (2019)	27	20	0.74 (0.55, 0.87)	3.84
Fichtner et al (2019)	27	22	0.81 (0.63, 0.92)	3.91
Franzini et al (2010)	20	5	0.25 (0.11, 0.47)	3.75
Hashizume et al (2012)	28	5	0.18 (0.08, 0.36)	3.93
Hasiloglu et al (2012)	25	11	0.44 (0.27, 0.63)	3.73
He et al (2017)	354	25	0.07 (0.05, 0.10)	4.17
Hyun et al (2008)	31	14	0.45 (0.29, 0.62)	3.81
Idrissi et al (2015)	7	4	0.57 (0.25, 0.84)	2.93
Joo et al (2015)	126	44	0.35 (0.27, 0.44)	4.09
Lee et al (2018)	62	25	0.40 (0.29, 0.53)	3.99
Ohtonary et al (2018)	19	11	0.58 (0.36, 0.77)	3.61
Schievink et al (2005)	65	21	0.32 (0.22, 0.44)	4.01
So et al (2016)	23	1	0.04 (0.01, 0.21)	4.09
Su et al (2009)	12	7	0.58 (0.32, 0.81)	3.35
Takahashi et al (2016)	110	29	0.26 (0.19, 0.35)	4.09
Thielen et al (2015)	14	11	0.79 (0.52, 0.92)	3.64
Wu et al (2017)	150	127	0.85 (0.78, 0.90)	4.13
Yagi et al (2018)	18	15	0.83 (0.61, 0.94)	3.82
Yoshida et al (2014)	12	1	0.08 (0.01, 0.35)	3.88
Overall (I^2 = 97.26%, p = 0.00)			0.41 (0.29, 0.52)	100.00

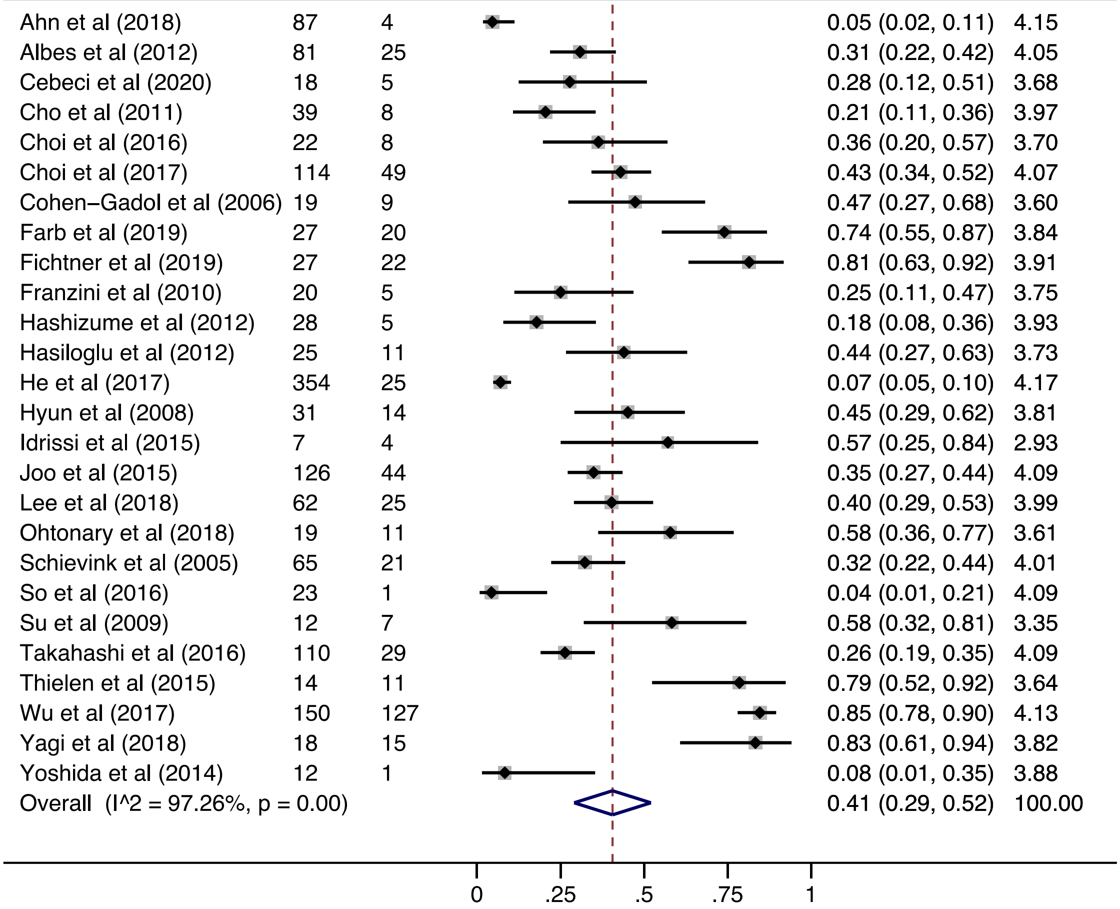

© 2021 D'Antona et al. *JAMA Neurology.*

eFigure 17d. Meta-analysis of proportions of leak location (thoraco-lumbar)

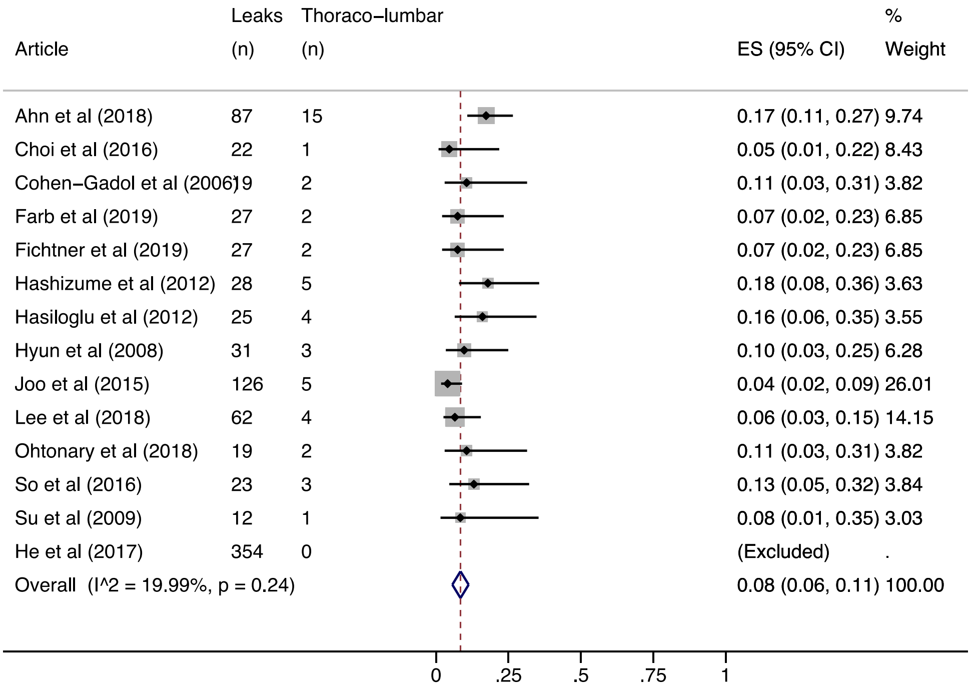

eFigure 17e. Meta-analysis of proportions of leak location (lumbar)

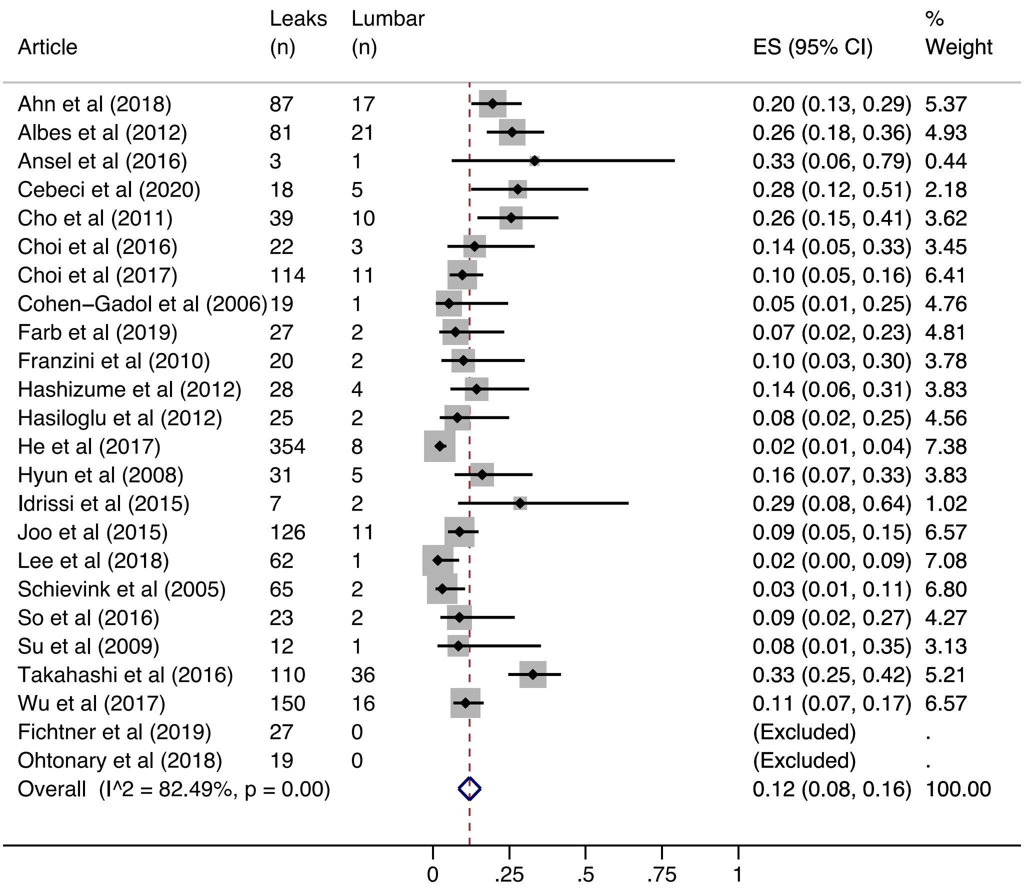

© 2021 D'Antona et al. *JAMA Neurology.*

eFigure 17f. Meta-analysis of proportions of leak location (sacral)

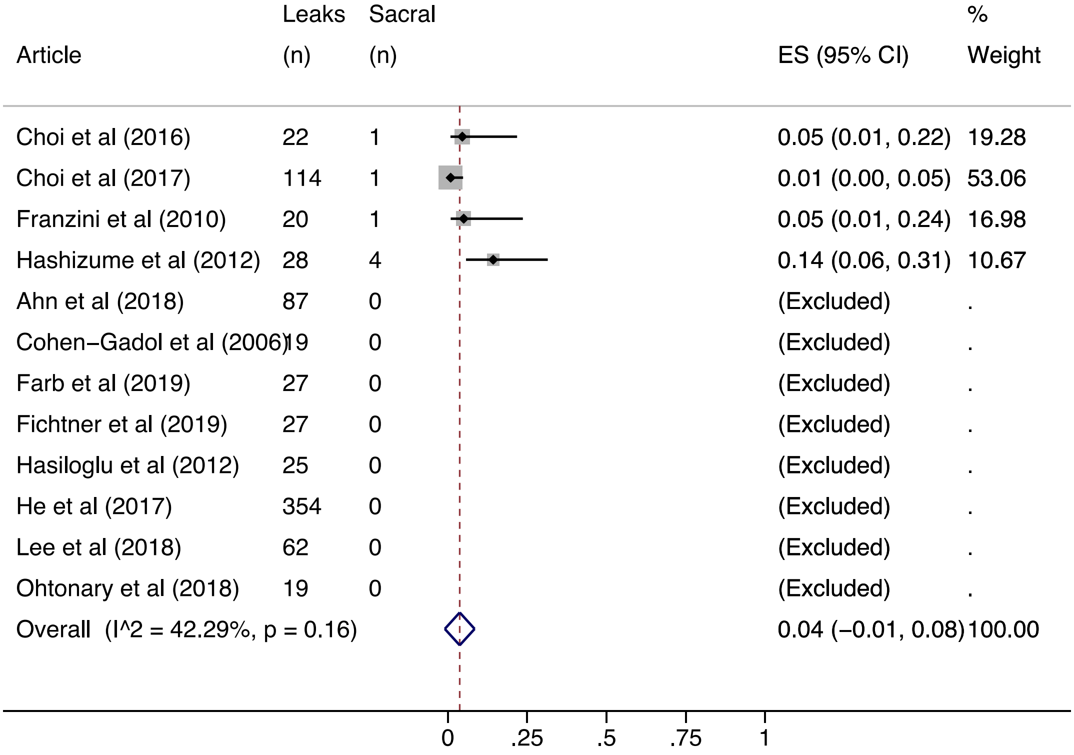

eFigure 17g. Meta-analysis of proportions of leak location (multiple)

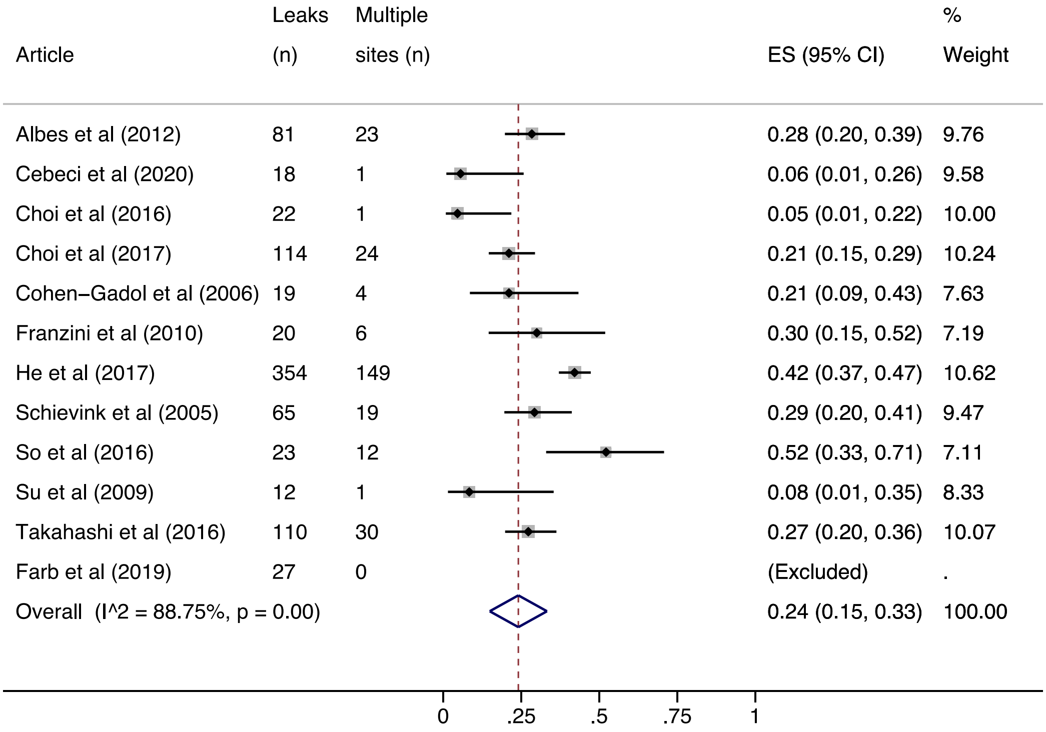

© 2021 D'Antona et al. *JAMA Neurology.*

eFigure 18a. Meta-analysis of proportions of lumbar puncture opening pressure (low)

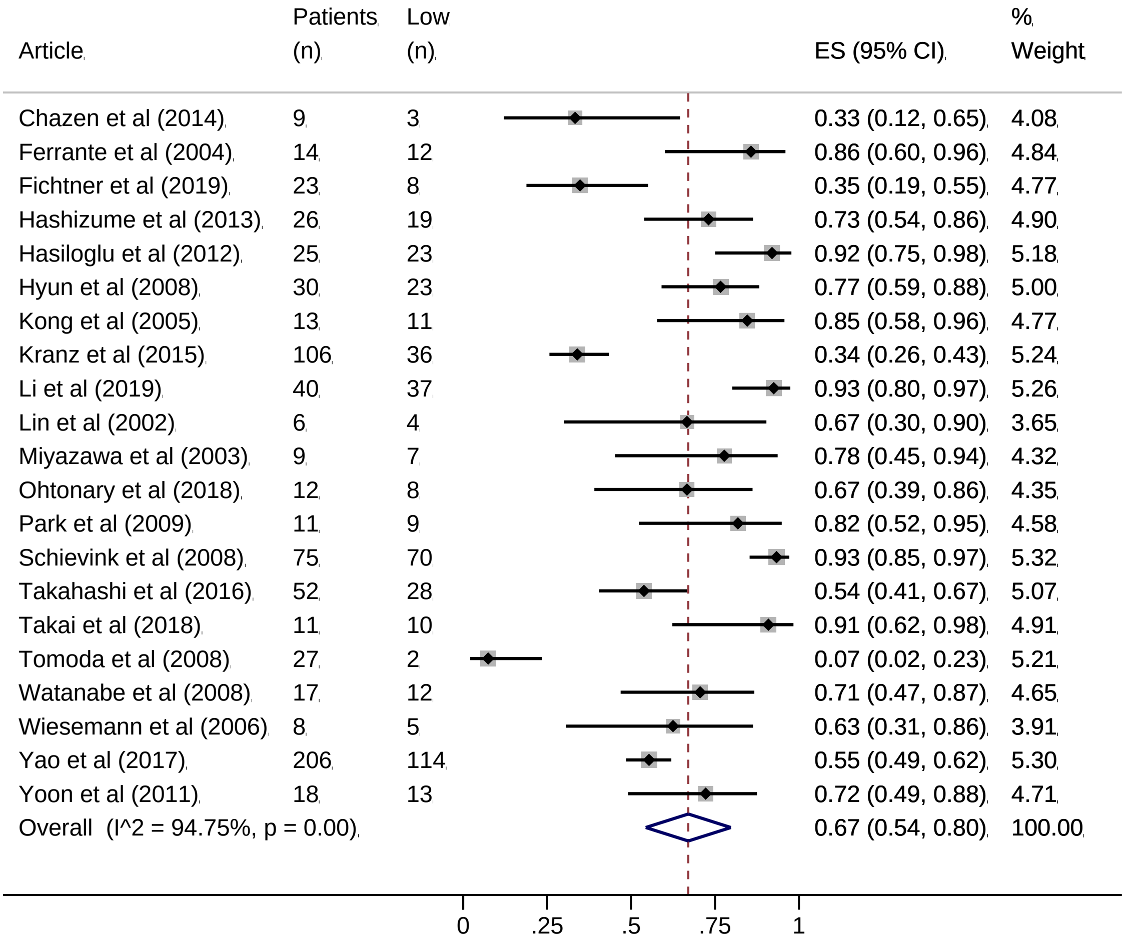

© 2021 D'Antona et al. *JAMA Neurology.*

eFigure 18b. Meta-analysis of proportions of lumbar puncture opening pressure (normal)

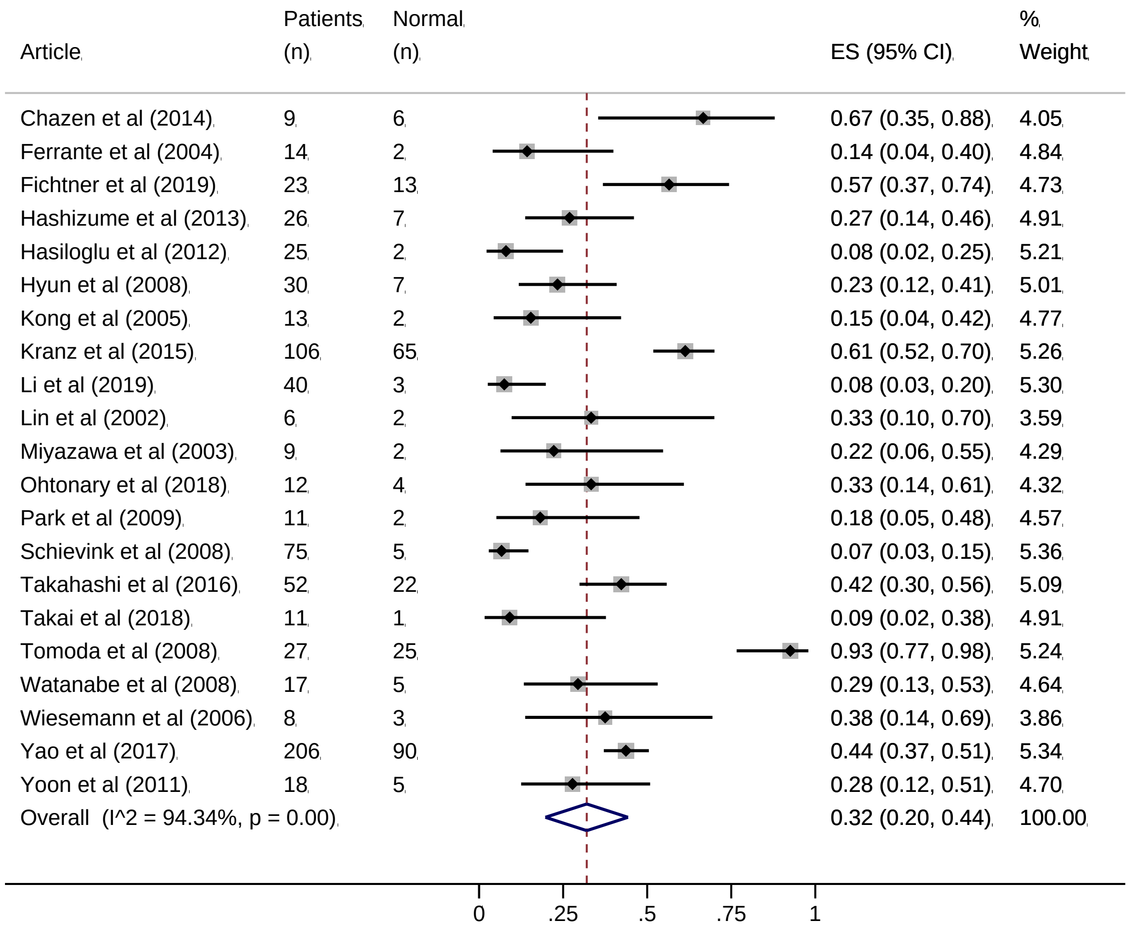

© 2021 D’Antona et al. *JAMA Neurology.*

eFigure 18c. Meta-analysis of proportions of lumbar puncture opening pressure (high)

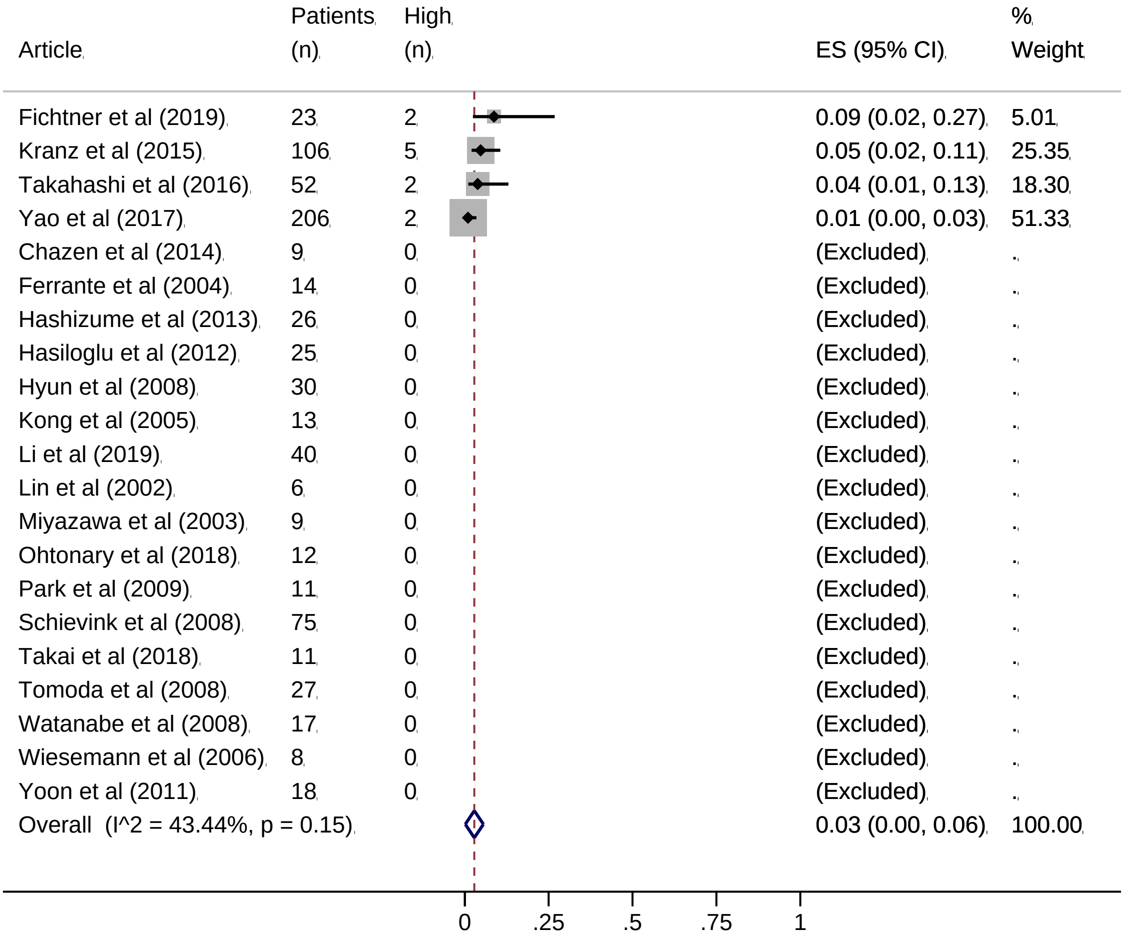

© 2021 D'Antona et al. *JAMA Neurology.*

eFigure 19a. Meta-analysis of proportions of successful conservative treatment

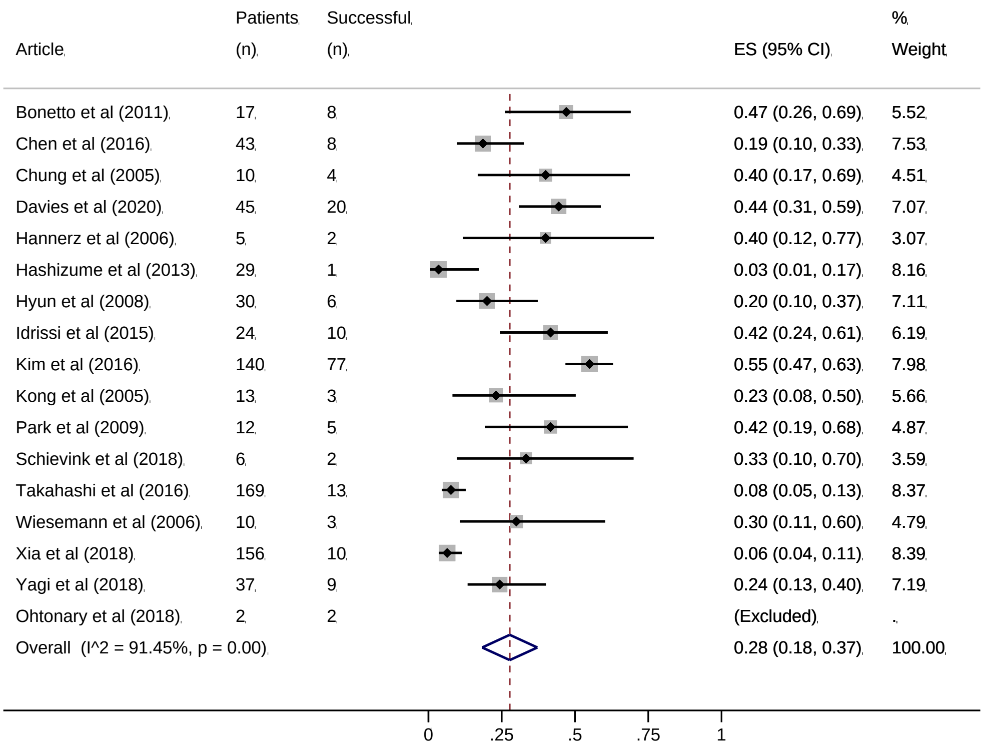

eFigure 19b. Meta-analysis of proportions of unsuccessful conservative treatment

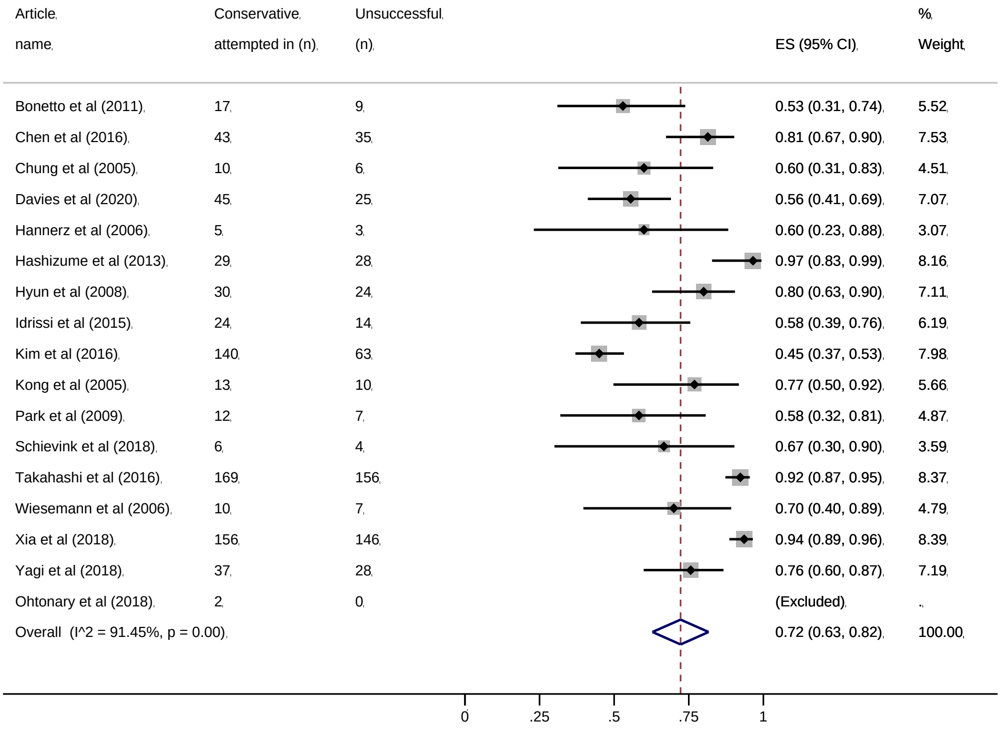

© 2021 D’Antona et al. *JAMA Neurology.*

eFigure 20a. Meta-analysis of proportions of successful first EBP

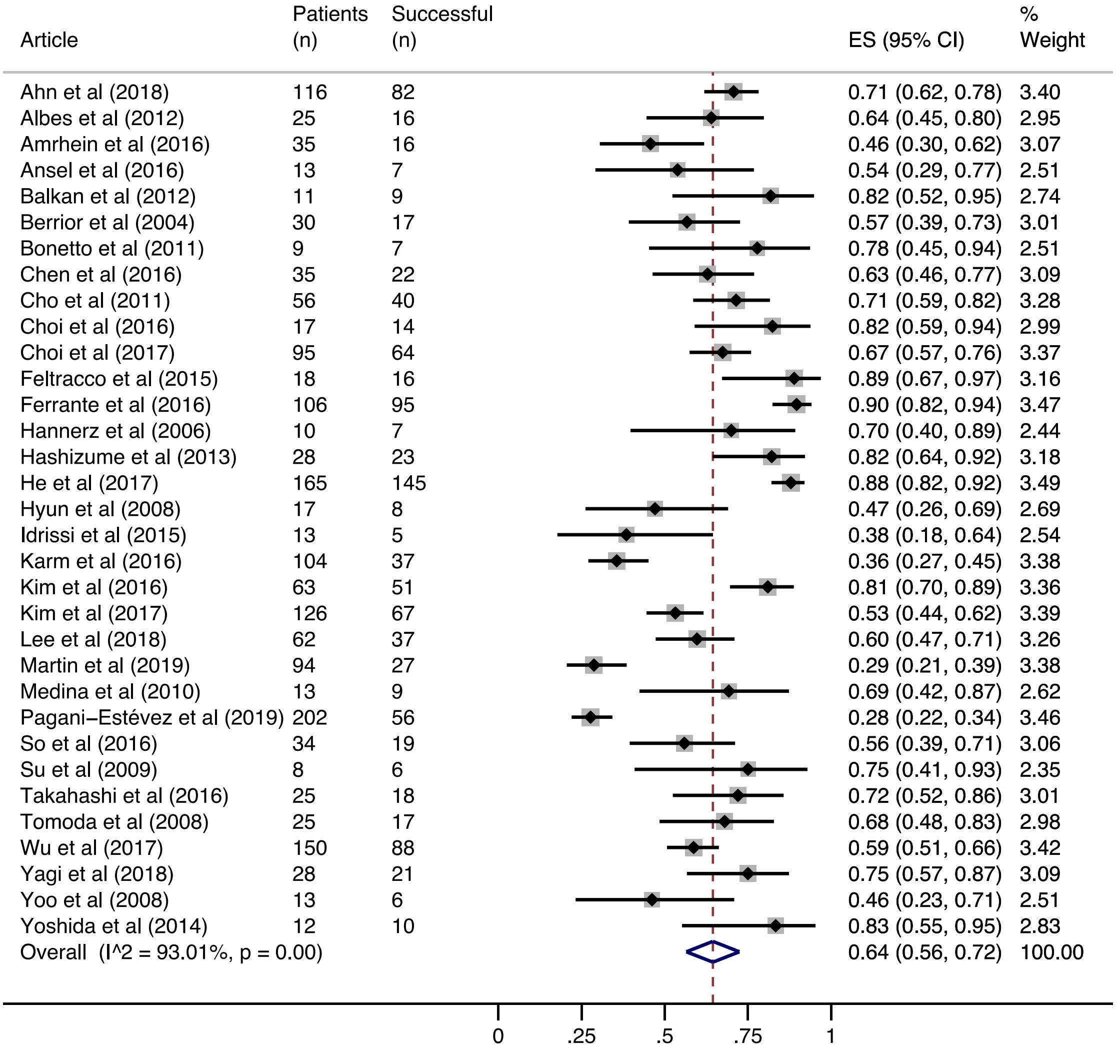

© 2021 D’Antona et al. *JAMA Neurology.*

eFigure 20b. Meta-analysis of proportions of successful nontargeted EBP

Article	Patients (n)	Successful (n)	ES (95% CI)	% Weight
Ahn et al (2018)	28	20	0.71 (0.53, 0.85)	11.91
Ansel et al (2016)	13	7	0.54 (0.29, 0.77)	5.95
Berrior et al (2004)	30	17	0.57 (0.39, 0.73)	11.07
Bonetto et al (2011)	9	7	0.78 (0.45, 0.94)	5.93
Cho et al (2011)	25	13	0.52 (0.33, 0.70)	9.70
Choi et al (2017)	22	15	0.68 (0.47, 0.84)	9.78
Feltracco et al (2015)	18	16	0.89 (0.67, 0.97)	14.05
Hannerz et al (2006)	10	7	0.70 (0.40, 0.89)	5.51
Levi et al (2019)	101	69	0.68 (0.59, 0.77)	21.08
Su et al (2009)	8	6	0.75 (0.41, 0.93)	5.03
Overall (I^2 = 34.90%, p = 0.13)			0.69 (0.61, 0.76)	100.00

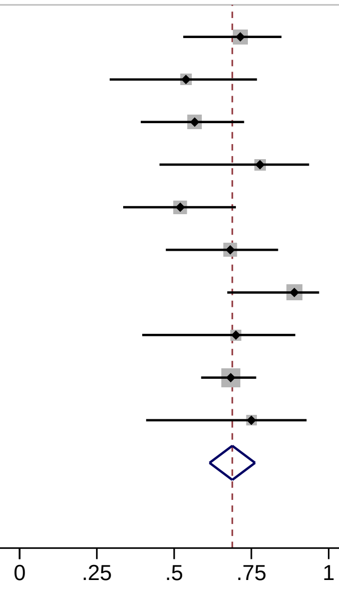

eFigure 20c. Meta-analysis of proportions of successful targeted EBP

Article	Patients (n)	Successful (n)	ES (95% CI)	% Weight
Ahn et al (2018)	88	62	0.70 (0.60, 0.79)	7.84
Albes et al (2012)	25	16	0.64 (0.45, 0.80)	6.52
Amrhein et al (2016)	35	16	0.46 (0.30, 0.62)	6.88
Balkan et al (2012)	11	9	0.82 (0.52, 0.95)	5.89
Cho et al (2011)	31	27	0.87 (0.71, 0.95)	7.56
Choi et al (2016)	17	14	0.82 (0.59, 0.94)	6.62
Choi et al (2017)	73	49	0.67 (0.56, 0.77)	7.69
Hashizume et al (2013)	28	23	0.82 (0.64, 0.92)	7.22
He et al (2017)	165	145	0.88 (0.82, 0.92)	8.26
Horikoshi et al (2010)	15	11	0.73 (0.48, 0.89)	5.95
Karm et al (2016)	104	37	0.36 (0.27, 0.45)	7.88
Lee et al (2018)	62	37	0.60 (0.47, 0.71)	7.50
Wu et al (2017)	150	88	0.59 (0.51, 0.66)	8.02
Yoshida et al (2014)	12	10	0.83 (0.55, 0.95)	6.16
Overall (I^2 = 90.50%, p = 0.00)			0.70 (0.59, 0.80)	100.00

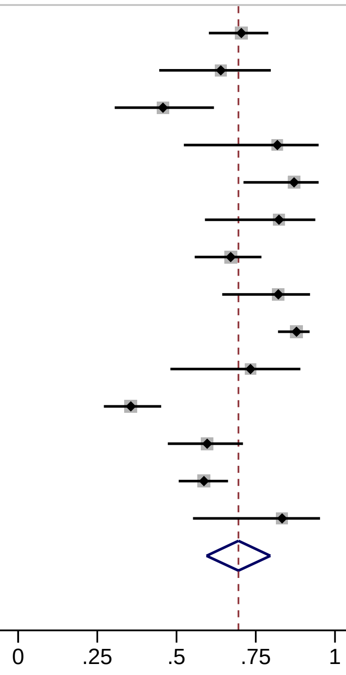

© 2021 D'Antona et al. *JAMA Neurology.*

eFigure 20d. Meta-analysis of proportions of successful small EBP

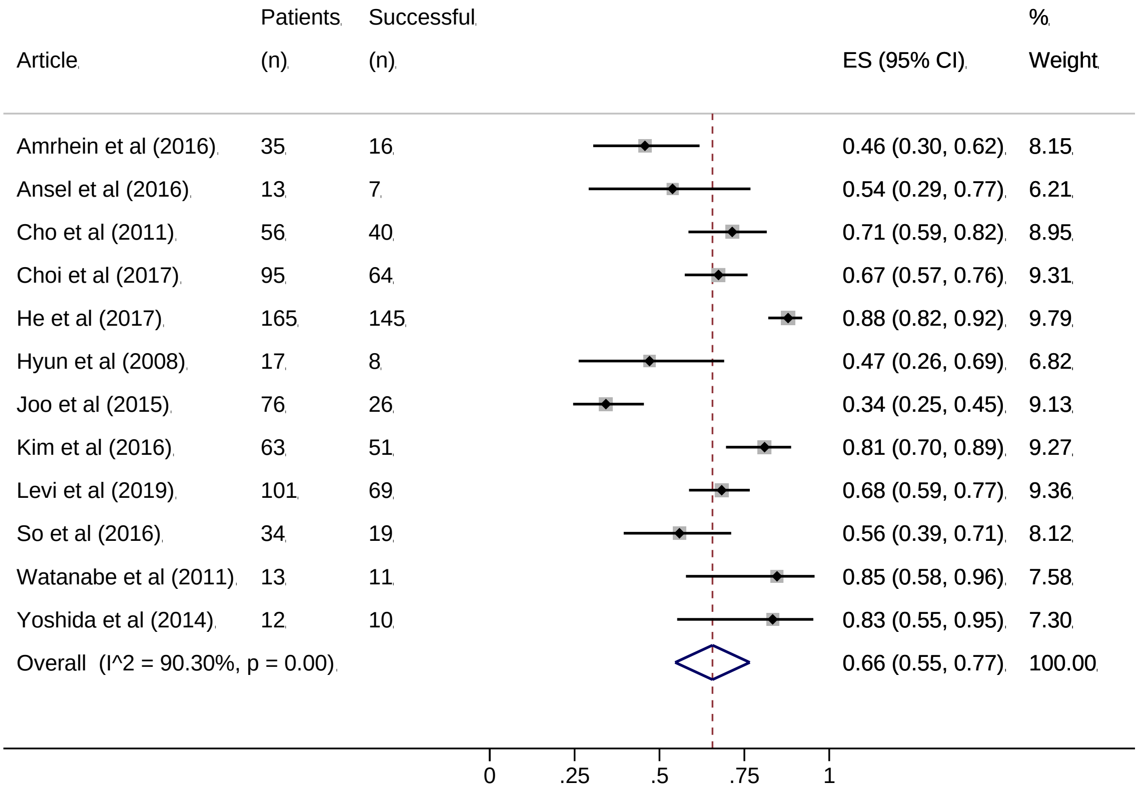

eFigure 20e. Meta-analysis of proportions of successful large EBP

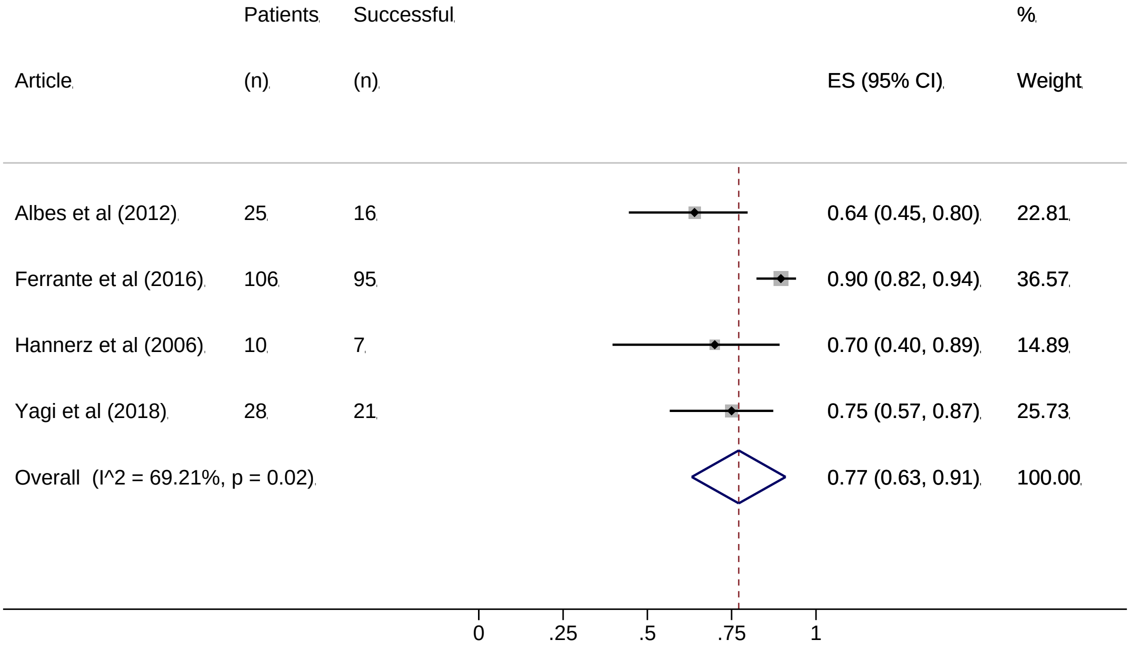

© 2021 D'Antona et al. *JAMA Neurology.*

eFigure 21. Epidural Blood Patches (EBPs) outcomes. Pooled estimates of proportions (95% CI) of successful EBP treatment stratified by EBP technique (non-targeted/targeted, small/large)

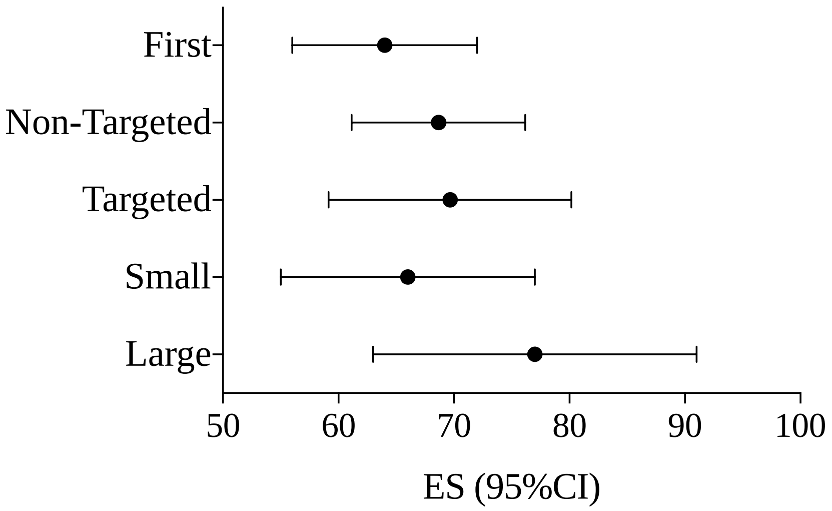

© 2021 D'Antona et al. *JAMA Neurology.*

eReferences

1. Cebeci H, Bilgin C, Candan S, Demir AB, Hakyemez B. Spinal Cerebrospinal Fluid Leakage in Spontaneous Intracranial Hypotension: An Intrathecal Gadolinium Enhanced MR-Myelography Study. J Belg Soc Radiol. 2020;104(1):6.
2. Davies MJ, Davies MA, Sharpe R, Cordato D, Schwartz R. Epidural Blood Patch as a Diagnostic and Therapeutic Intervention in Spontaneous Intracranial Hypotension: A Novel Approach to Management. World Neurosurg. 2020;137:e242-e50.
3. Wang TY, Karikari IO, Amrhein TJ, Gray L, Kranz PG. Clinical Outcomes Following Surgical Ligation of Cerebrospinal Fluid-Venous Fistula in Patients With Spontaneous Intracranial Hypotension: A Prospective Case Series. Oper Neurosurg (Hagerstown). 2020;18(3):239-45.
4. Dobrocky T, Grunder L, Breiding PS, Branca M, Limacher A, Mosimann PJ, et al. Assessing Spinal Cerebrospinal Fluid Leaks in Spontaneous Intracranial Hypotension With a Scoring System Based on Brain Magnetic Resonance Imaging Findings. JAMA Neurol. 2019;76(5):580-7.
5. Li C, Raza HK, Chansysouphanthong T, Zu J, Cui G. A clinical analysis on 40 cases of spontaneous intracranial hypotension syndrome. Somatosens Mot Res. 2019;36(1):24-30.
6. Levi V, Di Laurenzio NE, Franzini A, Tramacere I, Erbetta A, Chiapparini L, et al. Lumbar epidural blood patch: effectiveness on orthostatic headache and MRI predictive factors in 101 consecutive patients affected by spontaneous intracranial hypotension. J Neurosurg. 2019:1-9.

© 2021 D’Antona et al. *JAMA Neurology.*

7. Kim SC, Ryoo I, Sun HY, Park SW. MRI Findings of Spontaneous Intracranial Hypotension: Usefulness of Straight Sinus Distention. AJR Am J Roentgenol. 2019:1-7.

8. Schievink WI, Maya MM, Jean-Pierre S, Moser FG, Nuno M, Pressman BD. Rebound high-pressure headache after treatment of spontaneous intracranial hypotension: MRV study. Neurol Clin Pract. 2019;9(2):93-100.

9. Schievink WI, Maya MM, Moser FG, Prasad RS, Cruz RB, Nuno M, et al. Lateral decubitus digital subtraction myelography to identify spinal CSF-venous fistulas in spontaneous intracranial hypotension. J Neurosurg Spine. 2019:1-4.

10. Pagani-Estevez GL, Cutsforth-Gregory JK, Morris JM, Mokri B, Piepgras DG, Mauck WD, et al. Procedural predictors of epidural blood patch efficacy in spontaneous intracranial hypotension. Reg Anesth Pain Med. 2019.

11. Fichtner J, Ulrich CT, Fung C, Cipriani D, Gralla J, Piechowiak EI, et al. Sonography of the optic nerve sheath diameter before and after microsurgical closure of a dural CSF fistula in patients with spontaneous intracranial hypotension - a consecutive cohort study. Cephalalgia. 2019;39(2):306-15.

12. Martin R, Louy C, Babu V, Jiang Y, Far A, Schievink WI. A two-level large-volume epidural blood patch protocol for spontaneous intracranial hypothension: retrospective analysis of risk benefit. Reg Anesth Pain Med. 2020;45:32-7.

13. Kinsman KA, Verdoorn JT, Luetmer PH, Clark MS, Diehn FE. Renal Contrast on CT Myelography: Diagnostic Value in Patients with Spontaneous Intracranial Hypotension. AJNR Am J Neuroradiol. 2019;40(2):376-81.

14. Farb RI, Nicholson PJ, Peng PW, Massicotte EM, Lay C, Krings T, et al. Spontaneous Intracranial Hypotension: A Systematic Imaging Approach for CSF Leak Localization

© 2021 D’Antona et al. *JAMA Neurology.*

and Management Based on MRI and Digital Subtraction Myelography. AJNR Am J Neuroradiol. 2019;40(4):745-53.

15. Griffin AS, Lu L, Peacock S, Gray L, Kranz PG, Amrhein TJ. CSF volume provocation maneuvers during lumbar puncture as a possible predictive tool for diagnosing spontaneous intracranial hypotension. Clin Neurol Neurosurg. 2019;186:105552.

16. Ohtonari T, Ota S, Himeno T, Nishihara N, Sato M, Tanaka A. Excellent Outcomes of Large-Volume Epidural Blood Patch Using an Intravenous Catheter in 15 Consecutive Cases with Cerebrospinal Fluid Leak. World Neurosurg. 2018;118:e276-e82.

17. Murakami Y, Takahashi K, Hoshi K, Ito H, Kanno M, Saito K, et al. Spontaneous intracranial hypotension is diagnosed by a combination of lipocalin-type prostaglandin D synthase and brain-type transferrin in cerebrospinal fluid. Biochim Biophys Acta Gen Subj. 2018;1862(8):1835-42.

18. Lee JY, Lee MJ, Park HJ, Park JH, Jeong HJ, Oh MS, et al. Clinical effect of the proximity of epidural blood patch injection to the leakage site in spontaneous intracranial hypotension. Br J Neurosurg. 2018;32(6):671-3.

19. Schievink WI, Maya MM, Barnard ZR, Moser FG, Jean-Pierre S, Waxman AD, et al. Behavioral Variant Frontotemporal Dementia as a Serious Complication of Spontaneous Intracranial Hypotension. Oper Neurosurg (Hagerstown). 2018;15(5):505-15.

20. Xia P, Zhang SR, Zhou ZJ, Shao YQ, Hu XY. Benign paroxysmal positional vertigo in spontaneous intracranial hypotension. Neurol Res. 2018;40(10):868-73.

© 2021 D’Antona et al. *JAMA Neurology.*

21. Ahn C, Lee E, Lee JW, Chee CG, Kang Y, Kang HS. Two-site blind epidural blood patch versus targeted epidural blood patch in spontaneous intracranial hypotension. J Clin Neurosci. 2019;62:147-54.
22. Yagi T, Horikoshi T, Senbokuya N, Murayama H, Kinouchi H. Distribution Patterns of Spinal Epidural Fluid in Patients with Spontaneous Intracranial Hypotension Syndrome. Neurol Med Chir (Tokyo). 2018;58(5):212-8.
23. Tsai YH, Chen HC, Tung H, Wu YY, Chen HM, Pan KJ, et al. Noninvasive assessment of intracranial elastance and pressure in spontaneous intracranial hypotension by MRI. J Magn Reson Imaging. 2018;48(5):1255-63.
24. Takai K, Niimura M, Hongo H, Umekawa M, Teranishi A, Kayahara T, et al. Disturbed Consciousness and Coma: Diagnosis and Management of Intracranial Hypotension Caused by a Spinal Cerebrospinal Fluid Leak. World Neurosurg. 2019;121:e700-e11.
25. Dobrocky T, Mosimann PJ, Zibold F, Mordasini P, Raabe A, Ulrich CT, et al. Cryptogenic Cerebrospinal Fluid Leaks in Spontaneous Intracranial Hypotension: Role of Dynamic CT Myelography. Radiology. 2018;289(3):766-72.
26. Clark MS, Diehn FE, Verdoorn JT, Lehman VT, Liebo GB, Morris JM, et al. Prevalence of hyperdense paraspinal vein sign in patients with spontaneous intracranial hypotension without dural CSF leak on standard CT myelography. Diagn Interv Radiol. 2018;24(1):54-9.
27. Ferrante E, Rubino F, Beretta F, Regna-Gladin C, Ferrante MM. Treatment and outcome of subdural hematoma in patients with spontaneous intracranial hypotension: a report of 35 cases. Acta Neurol Belg. 2018;118(1):61-70.

© 2021 D’Antona et al. *JAMA Neurology.*

28. Beck J, Raabe A, Schievink WI, Fung C, Gralla J, Piechowiak E, et al. Posterior Approach and Spinal Cord Release for 360 degrees Repair of Dural Defects in Spontaneous Intracranial Hypotension. Neurosurgery. 2019;84(6):E345-E51.

29. Schievink WI, Maya MM, Moser FG, Jean-Pierre S, Nuno M. Coma: A serious complication of spontaneous intracranial hypotension. Neurology. 2018;90(19):e1638-e45.

30. Yao LL, Hu XY. Factors affecting cerebrospinal fluid opening pressure in patients with spontaneous intracranial hypotension. J Zhejiang Univ Sci B. 2017;18(7):577-85.

31. Wu JW, Hseu SS, Fuh JL, Lirng JF, Wang YF, Chen WT, et al. Factors predicting response to the first epidural blood patch in spontaneous intracranial hypotension. Brain. 2017;140(2):344-52.

32. Beck J, Fung C, Ulrich CT, Fiechter M, Fichtner J, Mattle HP, et al. Cerebrospinal fluid outflow resistance as a diagnostic marker of spontaneous cerebrospinal fluid leakage. J Neurosurg Spine. 2017;27(2):227-34.

33. He FF, Li L, Liu MJ, Zhong TD, Zhang QW, Fang XM. Targeted Epidural Blood Patch Treatment for Refractory Spontaneous Intracranial Hypotension in China. J Neurol Surg B Skull Base. 2018;79(3):217-23.

34. Choi H, Lee MJ, Choi HA, Cha J, Chung CS. Intracranial structural alteration predicts treatment outcome in patients with spontaneous intracranial hypotension. Cephalalgia. 2018;38(2):323-31.

35. Kim YJ, Cho HY, Seo DW, Sohn CH, Ahn S, Lee YS, et al. Misdiagnosis of Spontaneous Intracranial Hypotension as a Risk Factor for Subdural Hematoma. Headache. 2017;57(10):1593-600.

© 2021 D'Antona et al. *JAMA Neurology.*

36. Kranz PG, Amrhein TJ, Gray L. CSF Venous Fistulas in Spontaneous Intracranial Hypotension: Imaging Characteristics on Dynamic and CT Myelography. AJR Am J Roentgenol. 2017;209(6):1360-6.

37. Chen CH, Chen JH, Chen HC, Chai JW, Chen PL, Chen CC. Patterns of cerebrospinal fluid (CSF) distribution in patients with spontaneous intracranial hypotension: Assessed with magnetic resonance myelography. J Chin Med Assoc. 2017;80(2):109-16.

38. Chen HC, Chen PL, Tsai YH, Chen CH, Chen CC, Chai JW. Quantitative Measurement of CSF in Patients with Spontaneous Intracranial Hypotension. AJNR Am J Neuroradiol. 2017;38(5):1061-7.

39. Chen YC, Wang YF, Li JY, Chen SP, Lirng JF, Hseu SS, et al. Treatment and prognosis of subdural hematoma in patients with spontaneous intracranial hypotension. Cephalalgia. 2016;36(3):225-31.

40. So Y, Park JM, Lee P, Kim CL, Lee C, Kim JH. Epidural blood patch for the treatment of spontaneous and iatrogenic orthostatic headache. Pain Physician. 2016;19:E1115-E22.

41. Amemiya S, Takahashi K, Mima T, Yoshioka N, Miki S, Ohtomo K. Reversible alterations of the neuronal activity in spontaneous intracranial hypotension. Cephalalgia. 2016;36(2):162-71.

42. Kranz PG, Tanpitukpongse TP, Choudhury KR, Amrhein TJ, Gray L. Imaging Signs in Spontaneous Intracranial Hypotension: Prevalence and Relationship to CSF Pressure. AJNR Am J Neuroradiol. 2016;37(7):1374-8.

43. Schievink WI, Moser FG, Maya MM, Prasad RS. Digital subtraction myelography for the identification of spontaneous spinal CSF-venous fistulas. J Neurosurg Spine. 2016;24(6):960-4.

© 2021 D'Antona et al. *JAMA Neurology.*

44. Ferrante E, Rubino F, Mongelli M, Arpino I. Subarachnoideal blood spread following epidural blood patch given to treat spontaneous intracranial hypotension: Can it cause neurological complications? Clin Neurol Neurosurg. 2016;140:43-6.
45. Amrhein TJ, Befera NT, Gray L, Kranz PG. CT Fluoroscopy-Guided Blood Patching of Ventral CSF Leaks by Direct Needle Placement in the Ventral Epidural Space Using a Transforaminal Approach. AJNR Am J Neuroradiol. 2016;37(10):1951-6.
46. Fichtner J, Ulrich CT, Fung C, Knuppel C, Veitweber M, Jilch A, et al. Management of spontaneous intracranial hypotension - Transorbital ultrasound as discriminator. J Neurol Neurosurg Psychiatry. 2016;87(6):650-5.
47. Monteith TS, Kralik SF, Dillon WP, Hawkins RA, Goadsby PJ. The utility of radioisotope cisternography in low CSF/volume syndromes compared to myelography. Cephalalgia. 2016;36(13):1291-5.
48. Schievink WI, Maya MM, Jean-Pierre S, Nuno M, Prasad RS, Moser FG. A classification system of spontaneous spinal CSF leaks. Neurology. 2016;87:673-9.
49. Hori S, Taoka T, Miyasaka T, Ochi T, Sakamoto M, Wada T, et al. Dural Thickening of the Internal Auditory Canal in Patients With Spontaneous Intracranial Hypotension Syndrome. J Comput Assist Tomogr. 2016;40(2):297-300.
50. Karm MH, Choi JH, Kim D, Park JY, Yun HJ, Suh JH. Predictors of the Treatment Response of Spontaneous Intracranial Hypotension to an Epidural Blood Patch. Medicine (Baltimore). 2016;95(18):e3578.
51. Ansel S, Rae A, Tyagi A. Efficacy of epidural blood patches for spontaneous low-pressure headaches: a case series. J R Coll Physicians Edinb. 2016;46(4):234-7.
52. Takahashi K, Mima T, Akiba Y. Chronic Subdural Hematoma Associated with Spontaneous Intracranial Hypotension: Therapeutic Strategies and Outcomes of 55 Cases. Neurol Med Chir (Tokyo). 2016;56(2):69-76.

© 2021 D’Antona et al. *JAMA Neurology.*

53. Capizzano AA, Lai L, Kim J, Rizzo M, Gray L, Smoot MK, et al. Atypical Presentations of Intracranial Hypotension: Comparison with Classic Spontaneous Intracranial Hypotension. AJNR Am J Neuroradiol. 2016;37(7):1256-61.

54. Tanaka Y, Tosaka M, Fujimaki H, Honda F, Yoshimoto Y. Sex- and Age-Related Differences in the Clinical and Neuroimaging Characteristics of Patients With Spontaneous Intracranial Hypotension: A Records Review. Headache. 2016;56(8):1310-6.

55. Kim WJ, Shin HY, Kim YC, Moon JY. Clinical association between brain MRI findings with epidural blood patch in spontaneous intracranial hypotension. J Neurosurg Anesthesiol. 2016;28(2):147-52.

56. Choi SW, Ahn JM, Lee JW, Park KS, Kang HS. The usefulness of MDCT-myelography for patients with spontaneous intracranial hypotension. Springerplus. 2016;5:424.

57. Kranz PG, Amrhein TJ, Choudhury KR, Tanpitukpongse TP, Gray L. Time-Dependent Changes in Dural Enhancement Associated With Spontaneous Intracranial Hypotension. AJR Am J Roentgenol. 2016;207(6):1283-7.

58. Ferrante E, Olgiati E, Sangalli V, Rubino F. Early pain relief from orthostatic headache and hearing changes in spontaneous intracranial hypotension after epidural blood patch. Acta Neurol Belg. 2016;116(4):503-8.

59. Beck J, Ulrich CT, Fung C, Fichtner J, Seidel K, Fiechter M, et al. Diskogenic microspurs as a major cause of intractable spontaneous intracranial hypotension. Neurology. 2016;87:1220-6.

60. Verdoorn JT, Luetmer PH, Carr CM, Lane JI, Lehman VT, Morris JM, et al. Predicting High-Flow Spinal CSF Leaks in Spontaneous Intracranial Hypotension

Using a Spinal MRI-Based Algorithm: Have Repeat CT Myelograms Been Reduced? AJNR Am J Neuroradiol. 2016;37(1):185-8.

61. Wan Y, Xie J, Xie D, Xue Z, Wang Y, Yang S. Clinical characteristics of 15 cases of chronic subdural hematomas due to spontaneous intracranial hypotension with spinal cerebrospinal fluid leak. Acta Neurol Belg. 2016;116(4):509-12.

62. Kranz PG, Tanpitukpongse TP, Choudhury KR, Amrhein TJ, Gray L. How common is normal cerebrospinal fluid pressure in spontaneous intracranial hypotension? Cephalalgia. 2016;36(13):1209-17.

63. Idrissi AL, Lacour JC, Klein O, Schmitt E, Ducrocq X, Richard S. Spontaneous Intracranial Hypotension: Characteristics of the Serious Form in a Series of 24 Patients. World Neurosurg. 2015;84(6):1613-20.

64. Thielen KR, Sillery JC, Morris JM, Hoxworth JM, Diehn FE, Wald JT, et al. Ultrafast dynamic computed tomography myelography for the precise identification of high-flow cerebrospinal fluid leaks caused by spiculated spinal osteophytes. J Neurosurg Spine. 2015;22(3):324-31.

65. Joo EY, Hwang BY, Kong YG, Lee JH, Hwang BS, Suh JH. Retrospective study of epidural blood patch use for spontaneous intracranial hypotension. Reg Anesth Pain Med. 2015;40(1):58-61.

66. Feltracco P, Galligioni H, Barbieri S, Ori C. Thoracic epidural blood patches in the treatment of spontaneous intracranial hypotension: a retrospective case series. Pain Physician. 2015;18:343-8.

67. Schievink WI, Maya MM, Chu RM, Moser F. False localizing sign of cervico-thoracic CSF leak in spontaneous intracranial hypotension. 2015;84:2445-8.

© 2021 D'Antona et al. *JAMA Neurology.*

68. Schievink WI, Nuno M, Rozen TD, Maya MM, Mamelak AN, Carmichael J, et al. Hyperprolactinemia due to spontaneous intracranial hypotension. J Neurosurg. 2015;122(5):1020-5.

69. Xia P, Hu XY, Wang J, Hu BB, Xu QL, Zhou ZJ, et al. Risk factors for subdural haematoma in patients with spontaneous intracranial hypotension. PLoS One. 2015;10(4):e0123616.

70. Yoshida H, Takai K, Taniguchi M. Leakage detection on CT myelography for targeted epidural blood patch in spontaneous cerebrospinal fluid leaks: calcified or ossified spinal lesions ventral to the thecal sac. J Neurosurg Spine. 2014;21(3):432-41.

71. Chazen JL, Talbott JF, Lantos JE, Dillon WP. MR myelography for identification of spinal CSF leak in spontaneous intracranial hypotension. AJNR Am J Neuroradiol. 2014;35(10):2007-12.

72. Pimienta AL, Rimoin DL, Pariani M, Schievink WI, Reinstein E. Echocardiographic findings in patients with spontaneous CSF leak. J Neurol. 2014;261(10):1957-60.

73. Schievink WI, Goseland A, Cunneen S. Bariatric surgery as a possible risk factor for spontaneous intracranial hypotension. Neurology. 2014;83:1819-22.

74. Tung H, Liao YC, Wu CC, Chang MH, Chen CC, Chen PL, et al. Usefulness of phase-contrast magnetic resonance imaging for diagnosis and treatment evaluation in patients with SIH. Cephalalgia. 2014;34(8):584-93.

75. Hashizume K, Watanabe K, Kawaguchi M, Fujiwara A, Furuya H. Evaluation on a clinical course of subdural hematoma in patients undergoing epidural blood patch for spontaneous cerebrospinal fluid leak. Clin Neurol Neurosurg. 2013;115(8):1403-6.

76. Schievink WI, Maya MM, Louy C, Moser FG, Sloninsky L. Spontaneous intracranial hypotension in childhood and adolescence. J Pediatr. 2013;163(2):504-10.

© 2021 D’Antona et al. *JAMA Neurology.*

77. Hosoya T, Hatazawa J, Sato S, Kanoto M, Fukao A, Kayama T. Floating dural sac sign is a sensitive magnetic resonance imaging finding of spinal cerebrospinal fluid leakage. Neurol Med Chir (Tokyo). 2013;53:207-12.

78. Ferrante E, Regna-Gladin C, Arpino I, Rubino F, Porrinis L, Ferrante MM, et al. Pseudo-subarachnoid hemorrhage: a potential imaging pitfall associated with spontaneous intracranial hypotension. Clin Neurol Neurosurg. 2013;115(11):2324-8.

79. Franzini A, Messina G, Chiapparini L, Bussone G. Treatment of spontaneous intracranial hypotension: evolution of the therapeutic and diagnostic modalities. Neurol Sci. 2013;34 Suppl 1:S151-5.

80. Kranz PG, Stinnett SS, Huang KT, Gray L. Spinal meningeal diverticula in spontaneous intracranial hypotension: analysis of prevalence and myelographic appearance. AJNR Am J Neuroradiol. 2013;34(6):1284-9.

81. Reinstein E, Pariani M, Bannykh S, Rimoin DL, Schievink WI. Connective tissue spectrum abnormalities associated with spontaneous cerebrospinal fluid leaks: a prospective study. Eur J Hum Genet. 2013;21(4):386-90.

82. Albes G, Weng H, Horvath D, Musahl C, Bazner H, Henkes H. Detection and treatment of spinal CSF leaks in idiopathic intracranial hypotension. Neuroradiology. 2012;54(12):1367-73.

83. Hasiloglu ZI, Albayram S, Gorucu Y, Selcuk H, Cagil E, Erdemli HE, et al. Assessment of CSF flow dynamics using PC-MRI in spontaneous intracranial hypotension. Headache. 2012;52(5):808-19.

84. Hashizume K, Watanabe K, Kawaguchi M, Taoka T, Shinkai T, Furuya H. Comparison of computed tomography myelography and radioisotope cisternography to detect cerebrospinal fluid leakage in spontaneous intracranial hypotension. Spine (Phila Pa 1976). 2012;37(4):E237-42.

© 2021 D’Antona et al. *JAMA Neurology.*

85. Akbar JJ, Luetmer PH, Schwartz KM, Hunt CH, Diehn FE, Eckel LJ. The role of MR myelography with intrathecal gadolinium in localization of spinal CSF leaks in patients with spontaneous intracranial hypotension. AJNR Am J Neuroradiol. 2012;33(3):535-40.
86. Luetmer PH, Schwartz KM, Eckel LJ, Hunt CH, Carter RE, Diehn FE. When should I do dynamic CT myelography? Predicting fast spinal CSF leaks in patients with spontaneous intracranial hypotension. AJNR Am J Neuroradiol. 2012;33(4):690-4.
87. Balkan, II, Albayram S, Ozaras R, Yilmaz MH, Ozbayrak M, Mete B, et al. Spontaneous intracranial hypotension syndrome may mimic aseptic meningitis. Scand J Infect Dis. 2012;44(7):481-8.
88. Schievink WI, Schwartz MS, Maya MM, Moser FG, Rozen TD. Lack of causal association between spontaneous intracranial hypotension and cranial cerebrospinal fluid leaks. J Neurosurg. 2012;116(4):749-54.
89. Schievink WI, Chu RM, Maya MM, Johnson JP, Cohen HC. Spinal manifestations of spontaneous intracranial hypotension. J Neurosurg Spine. 2013;18(1):96-101.
90. Takeuchi N, Horikoshi T, Kinouchi H, Watanabe A, Yagi T, Mitsuka K, et al. Diagnostic value of the optic nerve sheath subarachnoid space in patients with intracranial hypotension syndrome. J Neurosurg. 2012;117(2):372-7.
91. Yoon SH, Chung YS, Yoon BW, Kim JE, Paek SH, Kim DG. Clinical experiences with spontaneous intracranial hypotension: a proposal of a diagnostic approach and treatment. Clin Neurol Neurosurg. 2011;113(5):373-9.
92. Cho KI, Moon HS, Jeon HJ, Park K, Kong DS. Spontaneous intracranial hypotension: Efficacy of radiologic targeting vs blind blood patch. Neurology. 2011;76:1139-44.
93. Bonetto N, Manara R, Citton V, Cagnin A. Spinal subtraction MRI for diagnosis of epidural leakage in SIH. Neurology. 2011;77:1873-6.

© 2021 D’Antona et al. *JAMA Neurology.*

94. Watanabe K, Hashizume K, Kawaguchi M, Fujiwara A, Sasaoka N, Furuya H. Fluoroscopically guided epidural blood patch with subsequent spinal CT scans in the treatment of spontaneous cerebrospinal fluid hypovolemia. J Neurosurg. 2011;114(6):1731-5.
95. Franzini A, Messina G, Mea E, Chiapparini L, Bussone G. Spontaneous intracranial hypotension: diagnostic and therapeutic implications in neurosurgical practice. Neurol Sci. 2011;32 Suppl 3:S287-90.
96. Liu FC, Fuh JL, Wang YF, Wang SJ. Connective tissue disorders in patients with spontaneous intracranial hypotension. Cephalalgia. 2011;31(6):691-5.
97. Franzini A, Messina G, Nazzi V, Mea E, Leone M, Chiapparini L, et al. Spontaneous intracranial hypotension syndrome: a novel speculative physiopathological hypothesis and a novel patch method in a series of 28 consecutive patients. J Neurosurg. 2010;112(2):300-6.
98. Medina JH, Abrams K, Falcone S, Bhatia RG. Spinal imaging findings in spontaneous intracranial hypotension. AJR Am J Roentgenol. 2010;195(2):459-64.
99. Ferrante E, Arpino I, Citterio A, Wetzl R, Savino A. Epidural blood patch in Trendelenburg position pre-medicated with acetazolamide to treat spontaneous intracranial hypotension. Eur J Neurol. 2010;17(5):715-9.
100. Horikoshi T, Watanabe A, Uchida M, Kinouchi H. Effectiveness of an epidural blood patch for patients with intracranial hypotension syndrome and persistent spinal epidural fluid collection after treatment. J Neurosurg. 2010;113(5):940-6.
101. Adachi M, Mugikura S, Shibata A, Kawaguchi E, Sato T, Takahashi S. Relative decrease in signal intensity of subcortical white matter in spontaneous intracranial hypotension on fluid-attenuated inversion recovery images. AJNR Am J Neuroradiol. 2009;30(5):906-10.

© 2021 D'Antona et al. *JAMA Neurology.*

102. Park ES, Kim E. Spontaneous intracranial hypotension: clinical presentation, imaging features and treatment. J Korean Neurosurg Soc. 2009;45:1-4.

103. Su CS, Lan MY, Chang YY, Lin WC, Liu KT. Clinical features, neuroimaging and treatment of spontaneous intracranial hypotension and magnetic resonance imaging evidence of blind epidural blood patch. Eur Neurol. 2009;61(5):301-7.

104. Mea E, Chiapparini L, Savoiardo M, Franzini A, Grimaldi D, Bussone G, et al. Application of IHS criteria to headache attributed to spontaneous intracranial hypotension in a large population. Cephalalgia. 2009;29(4):418-22.

105. Wang YF, Lirng JF, Fuh JL, Hseu SS, Wang SJ. Heavily T2-weighted MR myelography vs CT myelography in spontaneous intracranial hypotension. Neurology. 2009;73:1892-8.

106. Shankar JJ, Chakraborty S, Lum C. The venous hinge--an objective sign for the diagnosis and follow-up of treatment in patients with intracranial hypotension syndrome. Neuroradiology. 2009;51(7):453-6.

107. Mea E, Chiapparini L, Savoiardo M, Franzini A, Bussone G, Leone M. Clinical features and outcomes in spontaneous intracranial hypotension: a survey of 90 consecutive patients. Neurol Sci. 2009;30 Suppl 1:S11-3.

108. Watanabe A, Horikoshi T, Uchida M, Koizumi H, Yagishita T, Kinouchi H. Diagnostic value of spinal MR imaging in spontaneous intracranial hypotension syndrome. AJNR Am J Neuroradiol. 2009;30(1):147-51.

109. Schievink WI, Maya MM, Louy C, Moser FG, Tourje J. Diagnostic criteria for spontaneous spinal CSF leaks and intracranial hypotension. AJNR Am J Neuroradiol. 2008;29(5):853-6.

© 2021 D’Antona et al. *JAMA Neurology.*

110. Yoo HM, Kim SJ, Choi CG, Lee DH, Lee JH, Suh DC, et al. Detection of CSF leak in spinal CSF leak syndrome using MR myelography: correlation with radioisotope cisternography. AJNR Am J Neuroradiol. 2008;29(4):649-54.

111. Albayram S, Kilic F, Ozer H, Baghaki S, Kocer N, Islak C. Gadolinium-enhanced MR cisternography to evaluate dural leaks in intracranial hypotension syndrome. AJNR Am J Neuroradiol. 2008;29(1):116-21.

112. Hyun SH, Lee KH, Lee SJ, Cho YS, Lee EJ, Choi JY, et al. Potential value of radionuclide cisternography in diagnosis and management planning of spontaneous intracranial hypotension. Clin Neurol Neurosurg. 2008;110(7):657-61.

113. Tomoda Y, Korogi Y, Aoki T, Morioka T, Takahashi H, Ohno M, et al. Detection of cerebrospinal fluid leakage: initial experience with three-dimensional fast spin-echo magnetic resonance myelography. Acta Radiol. 2008;49(2):197-203.

114. Fuh JL, Wang SJ, Lai TH, Hseu SS. The timing of MRI determines the presence or absence of diffuse pachymeningeal enhancement in patients with spontaneous intracranial hypotension. Cephalalgia. 2008;28(4):318-22.

115. Mea E, Chiapparini L, Savoiardo M, Franzini A, Bussone G, Leone M. Headache attributed to spontaneous intracranial hypotension. Neurol Sci. 2008;29 Suppl 1:S164-5.

116. Mea E, Savoiardo M, Chiapparini L, Casucci G, Bonavita V, Bussone G, et al. Headache and spontaneous low cerebrospinal fluid pressure syndrome. Neurol Sci. 2007;28 Suppl 2:S232-4.

117. Chung SJ, Ki CS, Lee MC, Lee JH. Fibrillin-1 gene analysis of Korean patients with spontaneous CSF hypovolemia. Headache. 2007;47(1):111-5.

© 2021 D’Antona et al. *JAMA Neurology.*

118. Farb RI, Forghani R, Lee SK, Mikulis DJ, Agid R. The venous distension sign: a diagnostic sign of intracranial hypotension at MR imaging of the brain. AJNR Am J Neuroradiol. 2007;28(8):1489-93.

119. Lai TH, Fuh JL, Lirng JF, Tsai PH, Wang SJ. Subdural haematoma in patients with spontaneous intracranial hypotension. Cephalalgia. 2007;27(2):133-8.

120. Schievink WI, Maya MM, Moser F, Tourje J, Torbati S. Frequency of spontaneous intracranial hypotension in the emergency department. J Headache Pain. 2007;8(6):325-8.

121. Schievink WI, Louy C. Precipitating factors of spontaneous spoinal CSF leaks and intracranial hypotension. Neurology. 2007;69:700-2.

122. Tsai PH, Fuh JL, Lirng JF, Wang SJ. Heavily T2-weighted MR myelography in patients with spontaneous intracranial hypotension: a case-control study. Cephalalgia. 2007;27(8):929-34.

123. Wiesemann E, Berding G, Goetz F, Windhagen A. Spontaneous intracranial hypotension: correlation of imaging findings with clinical features. Eur Neurol. 2006;56(4):204-10.

124. Chung SJ, Lee JH, Kim SJ, Kwun BD, Lee MC. Subdural hematoma in spontaneous CSF hypovolemia. Neurology. 2006;67:1088-9.

125. Hannerz J, Dahlgren G, Irestedt L, Meyerson B, Ericson K. Treatment of idiopathic intracranial hypotension: cervicothoracic and lumbar blood patch and peroral steroid treatment. Headache. 2006;46(3):508-11.

126. Cohen-Gadol AA, Mokri B, Piepgras DG, Meyer FB, Atkinson JL. Surgical anatomy of dural defects in spontaneous spinal cerebrospinal fluid leaks. Neurosurgery. 2006;58(4 Suppl 2):ONS-238-45; discussion ONS-45.

© 2021 D'Antona et al. *JAMA Neurology.*

127. Chung SJ, Lee JH, Im JH, Lee MC. Short- and long-term outcomes of spontaneous CSF hypovolemia. Eur Neurol. 2005;54(2):63-7.

128. Kong DS, Park K, Nam DH, Lee JI, Kim JS, Eoh W, et al. Clinical features and long-term results of spontaneous intracranial hypotension. Neurosurgery. 2005;57(1):91-6; discussion -6.

129. Schievink WI, Maya MM, Louy C. Cranial MRI predicts outcome of spontaneous intracranial hypotension. Neurology. 2005;64:1282-4.

130. Schievink WI, Maya MM, Moser F, Tourje J. Spectrum of subdural fluid collections in spontaneous intracranial hypotension. J Neurosurg. 2005;103:608-13.

131. Ferrante E, Wetzl R, Savino A, Citterio A, Protti A. Spontaneous cerebrospinal fluid leak syndrome: report of 18 cases. Neurol Sci. 2004;25 Suppl 3:S293-5.

132. Berroir S, Loisel B, Ducros A, Boukobza M, Tzourio C, Valade D, et al. Early epidural blood patch in spontaneous intracranial hypotension. Neurology. 2004;63:1950-1.

133. Ferrante E, Savino A, Sances G, Nappi G. Spontaneous intracranial hypotension syndrome: report of twelve cases. Headache. 2004:615-22.

134. Schievink WI, Gordon OK, Tourje J. Connective tissue disorders with spontaneous spinal cerebrospinal fluid leaks and intracranial hypotension: a prospective study. Neurosurgery. 2004;54(1):65-70; discussion -1.

135. Schievink WI, Maya MM, Tourje J. False localizing sign of C1-2 cerebrospinal fluid leak in spontaneous intracranial hypotension. J Neurosurg. 2004;100:639-44.

136. Miyazawa K, Shiga Y, Hasegawa T, Endoh M, Okita N, Higano S, et al. CSF hypovolemia vs intracranial hypotension in “spontaneous intracranial hypotension syndrome”. Neurology. 2003;60:941-7.

© 2021 D’Antona et al. *JAMA Neurology.*

137. Chen WT, Fuh JL, Lirng JF, Lu SR, Wu ZA, Wang SJ. Collapsed superior ophthalmic veins in patients with spontaneous intracranial hypotension. Neurology. 2003;61:1265-7.

138. Schievink WI. Misdiagnosis of spontaneous intracranial hypotension. Arch Neurol. 2003;60:1713-8.

139. Lin WC, Lirng JF, Fuh JL, Wang SJ, Chang FC, Ho CF, et al. MR findings of spontaneous intracranial hypotension. Acta Radiol. 2002;43:249-55.

140. Schrijver I, Schievink WI, Godfrey M, Meyer FB, Francke U. Spontaneous spinal cerebrospinal fluid leaks and minor skeletalfeatures of Marfan syndrome: a microfibrillopathy. J Neurosurg. 2002;96:483-9.

141. Chung SJ, Kim JS, Lee MC. Syndrome of cerebral spinal fluid hypovolemia. Neurology. 2000;55:1321-7.

142. Chen C-C, Luo C-L, Wang S-J, Chern C-M, Fuh J-L, Lin S-H, et al. Colour doppler imaging for diagnosis of intracranial hypotension. The Lancet. 1999;354(9181):826-9.

143. Schievink WI, Morreale V, Atkinson JL, Meyer FB, Piepgras DG, Ebersold MJ. Surgical treatment of spontaneous spinal cerebrospinal fluid leaks. 1998;88:243-6.

144. Schievink WI, Meyer FB, Atkinson JL, Mokri B. Spontaneous spinal cerebrospinal fluid leaks and intracranial hypotension. J Neurosurg. 1996;84:598-605.

© 2021 D’Antona et al. *JAMA Neurology.*
